# Supplementary material for: Stakeholders’ views of supporting asthma management in schools with a school-based asthma programme for primary school children: a qualitative study in Malaysia
Source: BMJ Open. 2022 Feb 7;12(2):e052058. doi: 10.1136/bmjopen-2021-052058 (PMC8823135; doi:10.1136/bmjopen-2021-052058)
Supplement: Supplementary data [file bmjopen-2021-052058supp001.pdf]

Borang soal selidik latar belakang
Socio-demographic questionnaire

Latar belakang
Background

Umur*(Age)* : ____________

Jantina*(Gender)* : ____________

Bangsa*(Ethnicity)* : ____________

Status perkahwinan *(Marital status)*:

Bujang*(Single)*/Bercerai *(Divorced)*/Berkahwin*(Married)*

Tahap pendidikan tertinggi *(Highest educational level achieved):*

Rendah*(Primary)*/Menengah *(Secondary)*/Pengajian Tinggi *(Tertiary)*

Pekerjaan *(Occupation)* : ______________________

Pengalaman berkerja (work experience) : ………… /tahun(year)

Anggaran pendapatan isi rumah

(Estimated family income): RM _________ /bulan(month)

Informasi mengenai asma
Information about asthma

Adakah anda menghidap asma *(Do you have asthma)?*

Ya *(yes)*/ Tidak *(No)*

Adakah ahli keluarga menghidap asma (Anyone in your family has asthma)?

Ya *(yes)*/ Tidak *(No)*

Jika Ya, sila nyatakan (If yes, (please specify):

____
