# Supplementary material for: Stakeholders’ views of supporting asthma management in schools with a school-based asthma programme for primary school children: a qualitative study in Malaysia
Source: BMJ Open. 2022 Feb 7;12(2):e052058. doi: 10.1136/bmjopen-2021-052058 (PMC8823135; doi:10.1136/bmjopen-2021-052058)
Supplement: Supplementary data [file bmjopen-2021-052058supp002.pdf]

Childhood Asthma
Participant Interview Topic Guide (school staff)

Preamble:

- **Ice-breaking and explain that there is no right or wrong answer**
- **Explain need to get consent for the interview and audio-recording. Obtain consents.**
- **Explain that the participant does not have to answer if he or she does not wish to do so**

Question	Prompts
**Asthma experience**	
Tell us your experience in dealing with children with asthma?	Your child? Your student?
What do you know about asthma?	Experience? Reading? Told by someone?
How do you know if a child's asthma is good or badly controlled?	Able to play? Easy breathing? No cough? How do you know about this?
How does a child's asthma disturb/not disturb he/her? How?<br>How does a child's asthma disturbs/not disturb you? How?	Activities affected e.g. attending school? *Pendidikan Jasmani dan kesihatan* (PJK)/sports?
In your opinion, how does having asthma makes a child feel?	Emotionally-friends isolation/bully? Shy to use medication?
**Asthma treatment**	
Do you know of any asthma treatment?	Pills? MDI? Spacer? Aerochamber?
How about complementary and alternative medicine for asthma?	
**Asthma management at school**	
What do you do when a student has an asthma attack?	Inhaler? Go to the hospital/clinic? Call parents?
How do you recognise a severe asthma attack?<br>What would you do if this happened?	Breathlessness? Unable to speak?
Is there anything else you would do?	Concern on the administration of medication eg MDI to student(s)? Rules or legislation?
Is there any rules/limitation for students with asthma at school?	Sports? Physical activity? Extracurricular activities?
Is there any asthma education or campaign at school?	How is it delivered? By whom? To who?
What do you think about asthma education at school? Is it necessary?	Feasible? How best to deliver? Parents and school staffs involvement? How often?
What do you think if school staffs are allowed to deliver/supervise the use of asthma medication e.g inhaler at school?	Good? Not good? Concerns?

Suggestions/ideas for intervention?<br><br>Any suggestion on how to improve asthma management at school?	Others? Timing – during or after school?
**Health belief about asthma**<br><br>Why do you think a child has asthma?<br><br>What do you think a child can do to prevent asthma?	 <br>Inherited? Infection? Environment?<br><br>Avoidance of food? Medication? Sports?
**Source of information**<br><br>Where and from whom did you get information about asthma?<br><br><br>Anything else that you want to share about childhood asthma?	 <br>Clinic? Family? Friends? Internet? How good is the information?

Childhood Asthma (CuT-AsthMa)
Participant Interview Topic Guide (School Health Team)

Preamble:

- **Ice-breaking and explain that there is no right or wrong answer**
- **Explain the need to get consent for the interview and audio-recording. Obtain consents.**
- **Explain that the participant does not have to answer if he or she does not wish to do so**

Question	Prompts
**Asthma experience**	
Tell us your experience about dealing with children with asthma?	A student? A patient? Your own child?
How do you know that they have asthma?	Symptoms e.g cough, wheezing? Informed by parents?
How do you know if the child's asthma is good or badly controlled?	Able to play? Easy breathing? No cough? Where do you know about this?
How does a student's asthma disturb/not disturb him/her?	Physically? Activities affected at school e.g PJK, absent from school?
How does a student's asthma disturbs/not disturb you? How?	
In your opinion, how does having asthma makes a student feel?	Emotionally-friends isolation/bully? Shy to use medication?
**Asthma treatment**	
Do you know of any asthma treatment?	Pills? MDI? Spacer? Aerochamber?
How about complementary and alternative medication for asthma?	
**Asthma management at school**	
What do you do when a student has asthma attack?	Inhaler? Go to hospital/clinic? Asthma action plan? How do you know this?
How do you recognise a severe asthma attack? What would you do if this happened?	Breathlessness? Unable to speak?
Is there anything else you would do?	
	Sports? Physical activity? Extracurricular activities?

Is there any rules/limitation for students with asthma at school?<br><br>Is there any asthma education or campaign at school?<br><br>What do you think of asthma education at school? Is it necessary? Is it feasible?<br><br>What is your opinion on teachers delivering/supervising the use of asthma medication e.g inhaler at school?<br><br>Any suggestion on how to improve asthma management at school?	How is it delivered? By whom? To who?<br><br>Feasible? How best to deliver? Parents and teachers involvement? How often?<br><br>Good? Not good? Any worries?
**Health belief about asthma**<br><br>Why do you think a child has asthma?<br><br>What do you think a child can do to prevent asthma?	Inherited? Infection? Environment?<br><br>Avoidance of food? Medication? Sports?
**Source of information**<br><br>Where and from whom did you get information about asthma?<br><br>Anything else that you want to share about asthma among children?	Clinic? Family? Friends? Internet? How good is the information?

Childhood Asthma (CuT-AsthMa)
Participant focus group topic guide (Healthcare professionals/policymakers)
(Interviews, which will be offered as an option to suit the convenience/preference of the participant, will follow the same topic guide)

Preamble:

- **Ice-breaking and explain that there is no right or wrong answer**
- **Explain the need to get consent for the focus group/interview and audio-recording. Obtain consents.**
- **Explain that the participant does not have to answer if he or she does not wish to do so**

Question	Prompts
**Asthma experience**<br><br>Tell us about your experience in dealing with children with asthma?<br><br>How do you know that they have asthma?<br><br>How do you know if the child's asthma is good or badly controlled?<br><br>How does a student's asthma disturb/not disturb him/her?<br>How does a student's asthma disturbs/not disturb you? How?<br><br>In your opinion, how does having asthma makes a student feel?	 <br>A student? A patient? Your child?<br><br>Symptoms e.g cough, wheezing? Informed by parents?<br><br>Able to play? Easy breathing? No cough? Where do you know about this?<br><br>Physically? Activities affected at school e.g PJK, absent from school?<br><br>Emotionally-friends isolation/bully? Shy to use medication?
**Asthma treatment**<br><br>Do you know of any asthma treatment?<br><br>How about complementary and alternative medicine for asthma?	 <br>Pills? MDI? Spacer? Aerochamber?
**Asthma management at school**<br><br>What do you do when a student has an asthma attack?<br><br>How do you recognise a severe asthma attack?<br>What would you do if this happened?<br><br>Is there anything else you would do?<br><br>Is there any rules/limitation for students with asthma at school?<br><br>Is there any asthma education or campaign at school?	 <br>Inhaler? Go to the hospital/clinic? Asthma action plan? How do you know this?<br><br>Breathlessness? Unable to speak?<br><br>Sports? Physical activity? Extracurricular activities?<br><br>How is it delivered? By whom? To who?
What do you think of asthma education at school? Is it necessary? Is it feasible?	Feasible? How best to deliver? Parents and teachers involvement? How often?

Ramdzan SN, *et al. BMJ Open* 2022; 12:e052058. doi: 10.1136/bmjopen-2021-052058

What is your opinion on teachers delivering/supervising the use of asthma medication e.g inhaler at school?<br><br>Any suggestion on how to improve asthma management at school?	Good? Not good? Any worries?
**Health belief about asthma**<br><br>Why do you think a child has asthma?<br><br>What do you think a child can do to prevent asthma?	 <br>Inherited? Infection? Environment?<br><br>Avoidance of food? Medication? Sports?
**Source of information**<br><br>Where and from whom did you get information about asthma?<br><br>Anything else that you want to share about asthma among children?	 <br>Clinic? Family? Friends? Internet? How good is the information?

Ramdzan SN, *et al. BMJ Open* 2022; 12:e052058. doi: 10.1136/bmjopen-2021-052058
