# Supplementary material for: Stakeholders’ views of supporting asthma management in schools with a school-based asthma programme for primary school children: a qualitative study in Malaysia
Source: BMJ Open. 2022 Feb 7;12(2):e052058. doi: 10.1136/bmjopen-2021-052058 (PMC8823135; doi:10.1136/bmjopen-2021-052058)
Supplement: Author's manuscript [file bmjopen-2021-052058.draft_revisions.pdf]

BMJ Open

BMJ Open is committed to open peer review. As part of this commitment we make the peer review history of every article we publish publicly available.

When an article is published we post the peer reviewers' comments and the authors' responses online. We also post the versions of the paper that were used during peer review. These are the versions that the peer review comments apply to.

The versions of the paper that follow are the versions that were submitted during the peer review process. They are not the versions of record or the final published versions. They should not be cited or distributed as the published version of this manuscript.

BMJ Open is an open access journal and the full, final, typeset and author-corrected version of record of the manuscript is available on our site with no access controls, subscription charges or pay-per-view fees (http://bmjopen.bmj.com).

If you have any questions on BMJ Open's open peer review process please email info.bmjopen@bmj.com

BMJ Open

Stakeholders' views of asthma and school-based asthma programme for primary school children: A qualitative study in Malaysia

Journal:	*BMJ Open*
Manuscript ID	bmjopen-2021-052058
Article Type:	Original research
Date Submitted by the Author:	06-Apr-2021
Complete List of Authors:	Ramdzan, Siti Nurkamilla; Universiti Malaya, Department of Primary Care Medicine; The University of Edinburgh Usher Institute of Population Health Sciences and Informatics, NIHR Global Health Research Unit on Respiratory Health (RESPIRE)<br>Khoo, Ee Ming; Universiti Malaya, Department of Primary Care Medicine<br>Liew, Su May; Universiti Malaya, Department of Primary Care Medicine<br>Cunningham, Steven; The University of Edinburgh Usher Institute of Population Health Sciences and Informatics, NIHR Global Health Research Unit on Respiratory Health (RESPIRE)<br>Pinnock, Hilary; The University of Edinburgh Usher Institute of Population Health Sciences and Informatics, NIHR Global Health Research Unit on Respiratory Health (RESPIRE)
Keywords:	Asthma < THORACIC MEDICINE, Paediatric thoracic medicine < PAEDIATRICS, QUALITATIVE RESEARCH, Community child health < PAEDIATRICS

SCHOLARONE™
Manuscripts

I, the Submitting Author has the right to grant and does grant on behalf of all authors of the Work (as defined in the below author licence), an exclusive licence and/or a non-exclusive licence for contributions from authors who are: i) UK Crown employees; ii) where BMJ has agreed a CC-BY licence shall apply, and/or iii) in accordance with the terms applicable for US Federal Government officers or employees acting as part of their official duties; on a worldwide, perpetual, irrevocable, royalty-free basis to BMJ Publishing Group Ltd ("BMJ") its licensees and where the relevant Journal is co-owned by BMJ to the co-owners of the Journal, to publish the Work in this journal and any other BMJ products and to exploit all rights, as set out in our licence.

The Submitting Author accepts and understands that any supply made under these terms is made by BMJ to the Submitting Author unless you are acting as an employee on behalf of your employer or a postgraduate student of an affiliated institution which is paying any applicable article publishing charge ("APC") for Open Access articles. Where the Submitting Author wishes to make the Work available on an Open Access basis (and intends to pay the relevant APC), the terms of reuse of such Open Access shall be governed by a Creative Commons licence – details of these licences and which Creative Commons licence will apply to this Work are set out in our licence referred to above.

Other than as permitted in any relevant BMJ Author's Self Archiving Policies, I confirm this Work has not been accepted for publication elsewhere, is not being considered for publication elsewhere and does not duplicate material already published. I confirm all authors consent to publication of this Work and authorise the granting of this licence.

For peer review only

Stakeholders' views of asthma and school-based asthma programme for primary school children: A qualitative study in Malaysia

Siti Nurkamilla Ramdzan, MMed[1,2], Ee Ming Khoo, MD[1], Su May Liew, DPhil[1], Steve Cunningham, PhD[2], Hilary Pinnock, MD*[2] on behalf of RESPIRE collaborators

[1]*Department of Primary Care Medicine, Faculty of Medicine, University of Malaya, Malaysia*

[2]*NIHR Global Health Research Unit on Respiratory Health (RESPIRE), Usher Institute, University of Edinburgh, United Kingdom*

Address for correspondence
*Professor Hilary Pinnock
NIHR Global Health Research Unit on Respiratory Health (RESPIRE),
Usher Institute,
The University of Edinburgh.
Doorway 3, Medical School, Teviot Place,
Edinburgh EH8 9AG, UK.
Tel: 0131 650 9474
Email: Hilary.Pinnock@ed.ac.uk

Main text word count: 3610 (excluding abstract, main headings, tables, references, and additional information, e.g. acknowledgement, funding, competing interest) Maximum: 4000 words

For peer review only

Abstract

Objective: The WHO Global School Health Initiative, which aimed to improve child and community health through health promotion programmes in schools, typically focusses on preventing communicable disease. Asthma, the commonest non-communicable disease among children potentially requiring urgent treatment during school hours, is an important exemplar for school-based programmes. We aimed to explore the views of school staff, healthcare professionals and policymakers, about asthma and school asthma programmes for primary school children.

Design: We recruited a purposive sampling of participants to obtain diverse views in a qualitative study. We conducted focus groups and individual interviews using piloted semi-structured topic guides, until we reached data saturation. The focus groups and interviews were audio-recorded, transcribed verbatim and analysed using inductive thematic analysis.

Setting: Stakeholders in education and health sectors in Malaysia.

Participants: Fifty-two participants (40 school staff, 9 healthcare professionals and 3 policymakers) contributed to nine focus groups and eleven individual interviews.

Results: School staff had limited awareness of asthma and first-aid asthma treatments. There was no guidance on asthma management in government schools, and teachers were unclear about their role in school children's health. These uncertainties led to delays in the treatment of asthma symptoms/attacks, and suggestions that an asthma education programme and a school plan would improve asthma care. Perceived challenges in conducting school health programmes included a busy school schedule and poor parental participation. A tailored asthma programme developed in partnership with schools and health services could facilitate adoption/ and implementation.

For peer review only - http://bmjopen.bmj.com/site/about/guidelines.xhtml

Conclusions: Identifying and addressing issues and challenges specific to the school and wider community would facilitate the delivery of a school asthma programme in line with the WHO School Health Initiative. Clarity over national policy on the role and responsibilities of school staff could support implementation and guide appropriate and prompt response to asthma emergencies in schools.

Word count: 298 (Maximum: 300)

Keywords: school-based, asthma, complex intervention, low-middle income countries and health behaviour

For peer review only

Strengths and limitation of this study

- One of a few studies on school asthma programmes conducted in low-middle income countries and aligned with WHO Global School Health Initiative.
- Participants from a broad range of cultural and professional backgrounds contributed to their diverse views, but we may have missed some perspectives.
- The analysis was conducted in the source language, but there may have been some loss of meaning during the translation of Malay quotes to English.
- Participants with less experience on the topic may have contributed less to the focus groups though they were provided with opportunities to provide their views.

Introduction

The Global School Health Initiative launched by the World Health Organisation in 1995 aimed to improve child, adolescent and community health through health promotion programmes in schools.[1] Globally, the compulsory school years offer an 'easy entry point' for embedding healthy behaviours at a young age and, by extension, influencing families.[2] Health promotion programmes (e.g. de-worming, vaccination, water, sanitation, hygiene) have been successfully implemented in low-middle income countries (LMICs).[2] Non-communicable diseases (NCDs) are an increasing challenge in all economies, though successful NCD programmes have typically been focussed in high-income countries.[2 3]

Asthma is the commonest NCD among children and causes significant morbidity and mortality globally.[4] Asthma symptoms and attacks can occur unexpectedly, and schools need plans for responding appropriately and promptly to asthma symptoms.[5 6] Young children can spend up to half their waking hours in schools, and school staff

For peer review only - http://bmjopen.bmj.com/site/about/guidelines.xhtml

could support the development of asthma self-management skills in the students.[7-9] A school asthma programme thus offers an important exemplar for school health NCDs programmes.[3 10]

School-based asthma self-management interventions have been shown to improve asthma control, reduce school absenteeism and asthma exacerbations[11-14], though few were conducted in LMICs.[11 14 15] These programmes are complex interventions that need to be tailored to the target population[16 17], and adapted to the context.[18] In a review of the first two decades of school health initiatives, the WHO highlighted the importance of policy and organisation-level collaboration and engagement with parents, students and teachers.[2] Building on a qualitative study of the views of primary school children with asthma and their parents[19 20], we therefore aimed to explore the views of school staff, healthcare professionals and policymakers about asthma and school-based asthma management to inform the development of a primary school asthma programme in Malaysia.

Methods

This qualitative study was conducted between May and December 2019 with ethical approval from the Malaysian National Medical Research and Ethics Committee (NMRR-18-3136-44042) and sponsorship from the Academic and Clinical Centre for Research and Development, University of Edinburgh (AC18113). We obtained permission from the schools, Malaysian Ministry of Health and Ministry of Education to conduct the study. All participants provided their written informed consent.

For peer review only

Context and sampling

About 98% of Malaysia's primary education takes place in government schools under the Ministry of Education.[21] In these schools, there are no school nurses and the healthcare of school children is provided by the school health team under the Ministry of Health, which provides general health screening and education.[22] We purposively sampled school staff (teachers and administrative staff) from five government schools (2 Malay, 2 Chinese and 1 Indian), to represent the three main ethnic groups and diverse cultures in Malaysia.[21] We also purposively invited participants from a non-governmental organisation and a private school, healthcare professionals including the school health team and policymakers to obtain views from the wider education community.

Data collection

We arranged focus group discussions (FGD) to enable peer interaction supplemented with in-depth individual interviews (IDI) to accommodate the availability and preference of participants.[23 24] We collected basic socio-demographic data (Supplementary 1) and developed piloted semi-structured topic guides (Supplementary 2) based on the biopsychosocial model and previous literature to stimulate discussion.[19 20 25 26] The core topics addressed were asthma experience and awareness, current school asthma management practice and ideas for improvement. All the interviews and focus groups were conducted either in Malay (the national language of Malaysia) or English and moderated by SNR, a researcher who is proficient in both languages. We anticipated up to 50 participants and concluded data collection when there were no new themes emerging based on our core topics. The

For peer review only

focus groups and interviews were audio-recorded, transcribed verbatim and analysed using Nvivo software version 11.

Language and translation

The transcripts were analysed in the source language to reduce loss of meaning with translation. Aided by the field notes, the Malay transcripts were translated to English by experienced bilingual translators, and SNR checked the translated transcripts for semantic and contextual equivalence. The translated transcripts were used for data sharing, e.g., the use of quotes to describe the findings in this report.

Data analysis

We used inductive thematic analysis to explore the views of the participants about asthma and school asthma management. It is a systematic but flexible approach which may be used to inform intervention development and evaluation.[23 27] SNR read the transcripts multiple times for data familiarisation and developed codes. Constant comparison of codes allowed the development of themes, and we then constructed a schema to describe the relationship between themes. Memos, field notes and discussions with the research team aided data interpretation.

Reflexivity and interpretation

SNR who undertook data collection and coding, is an academic family physician with more than 15 years of working experience in Malaysia. At the beginning of the focus groups/interviews, SNR explained the main purpose of the study, which was to improve the care of children with asthma and emphasised her position as a researcher in understanding participants' views without judgements. The analysis and results

For peer review only - http://bmjopen.bmj.com/site/about/guidelines.xhtml

were discussed with the multi-cultural team consisting of primary care physicians and a paediatrician from the UK and Malaysia, to ensure balanced interpretation. We used a pragmatic and constructivist approach but constantly reminded ourselves to be open to any possible emerging theory.

Patient and Public Involvement

Patient/parent advisors have contributed to the overall programme of work. We piloted the topic guides among teachers and health professionals when designing the study. They provided feedback on the approach and questions in the topic guides. We also shared the preliminary findings of our study with some participants and other stakeholders in stakeholder engagement meetings.

Results

We recruited 52 participants consisting of 40 school staff (including three from a private school), nine healthcare professionals (five from the school health team), two policymakers and a representative from the teachers' professional association. We conducted nine focus groups (up to six participants in each group and duration up to 40 minutes) and 11 individual interviews (duration up to 45 minutes). Table 1 shows the characteristics of the participants. The participants' age ranged from 23 to 58 years and their working experience ranged 1 to 35 years. A quarter of them (14) had a personal history of asthma or a family member with asthma.

Summary of findings

Figure 1 illustrates the themes and subthemes of the participants' views of asthma and school-based asthma programme. Four themes were constructed to inform the development of a school asthma programme; the first theme highlighted the problems

For peer review only

perceived by participants, the second reported the suggested solutions and the two following themes addressed the challenges and enablers.

Quotes describing the themes are provided in the text and additional quotes are provided in Tables 2 and 3.

Problems

Variable awareness of asthma

The awareness of asthma among the participants depended on their personal experience of having asthma or helping others to self-manage asthma, e.g., family members or students, and/or if they had received asthma training or education (Table 2). Participants with experience or training could describe symptoms and were familiar with the treatment for asthma symptoms/attacks. Other participants were less able to contribute to the discussion about asthma symptoms, treatment, and possible complications. They had several misconceptions, e.g., 'children with asthma must limit their physical activity', and 'asthma attacks can be anticipated'.

> *"JJ: I think if the child is having asthma (symptoms), it's best for the parents to bring their child to see the doctor. Can asthma attack suddenly occur when the child is well?*
> *Moderator: Yes.*
> *JJ: I don't know about that." (FGD 2, administrative staff)*

Reliance on generic medical school plans

All participants stated that there was no specific asthma school plan in government schools (Table 2). Current practice followed the general circulars and their generic school plan for any illness or medical emergency. In a situation when a child was unwell, teachers would contact the parents to fetch the child from school, and the parents decided to either treat the child on their own or to seek medical attention. In

For peer review only - http://bmjopen.bmj.com/site/about/guidelines.xhtml

205 an emergency, teachers would take the child for medical care if it was clear a child
206 needed urgent treatment, or the parent took too long to get to school.

> *"Usually what we do, first, is to call their parents. If their parents said, "okay, can bring them to the clinic". Then we will bring them to the nearest clinic. We will get a permission letter from the office." (UU, FGD 5, teacher)*

207 The teachers were unclear about their role in schoolchildren's health. The circulars
208 stated that staff members were not allowed to give medication to students, but this
209 was interpreted differently according to their personal views and school management
210 plan. Some teachers interpreted the circulars to only apply to non-prescribed
211 medication and would act *in loco parentis* and give treatment to children only if they
212 had clear instructions from their parents. A few teachers felt able to extend this to
213 supervising prescribed medication such as an inhaler (albeit not being sure of the
214 technique). Other teachers viewed medication use as the parents and children's'
215 responsibility and would not want to handle medication at all. They feared their action
216 might worsen the student's condition, e.g., giving the wrong dose, developing side
217 effects of medication, being blamed by parents as they were not trained to manage
218 medical conditions.

> *"Sometimes parents called the teachers and said, "my child has an inhaler in his beg. Please can you supervise him"... Usually children will know how to use it. We just supervise when he/she is using it." (ZZ, FGD 6, teacher)*

219 *No communicated individual asthma action plans*

220 The absence of a specific asthma school plan and lack of clarity about the health role
221 of teachers could delay treatment during asthma attacks. Teachers were unsure of
222 what to do when dealing with children with asthma symptoms, some *'panicked'* while
223 several teachers sought help from colleagues considered to have better skills in
224 managing asthma. Both the teachers and the school health team observed that many

For peer review only - http://bmjopen.bmj.com/site/about/guidelines.xhtml

225 children with asthma did not bring their reliever inhaler to school. Without a reliever
226 inhaler, teachers tried different strategies when their students had symptoms, e.g.,
227 relaxation and breathing techniques while waiting for the parents to arrive.

"I was shocked when she was breathless; I am not sure how to help her. I patted her back and massaged her a bit, she was better, but after that, she coughed, and I panicked and quickly called her mother." (AA, FGD 1, teacher)

228 Participants identified some poor asthma self-management practices on the part of
229 parents. Teachers observed that parents rarely approached the school regarding their
230 child's illness and only a few had shared their expectation/plan with teachers to
231 support self-management of their child's illness at school. Even when children had
232 brought an inhaler to school, the teachers had not been asked to supervise the use.

"The child I told you earlier, his parents did not inform us... Only two parents in this school informed us about their child's illness." (FE, IDI 5, teacher)

233 ***Suggested solutions***

234 Participants, particularly those with asthma experience and training, identified a need
235 for a school asthma programme to deliver asthma education and introduce asthma
236 school plan (Table 3). On the other hand, participants without asthma experience or
237 training initially thought a school asthma programme was unnecessary, though some
238 participants changed their views during the focus group discussions as they became
239 aware of the risk of asthma attacks occurring at school.

"Asthma is not common in school. So, I don't think it (asthma programme) is necessary. But during this discussion, I think teachers should know how to help children with asthma if a child has an attack. It's better." (YY, FGD 6, a teacher without asthma experience)

240 To improve the self-management of children with asthma, healthcare professionals
241 and some teachers suggested the school programme could target the children, their
242 parents and teachers as primary school children were not capable of self-managing

For peer review only - http://bmjopen.bmj.com/site/about/guidelines.xhtml

243 asthma independently. The children could be taught to improve their self-management
244 skills e.g., how to avoid asthma triggers, monitor their symptoms, use a reliever
245 inhaler, and get help if having asthma symptoms/attacks. To support the self-
246 management of the children, their parents and teachers could receive similar asthma
247 education.

> *"The child themselves need to know, the severity of the symptoms,... the parents should know that the child has asthma... in the school, in case of any emergency, the teachers must know the child's asthma condition and how severe the asthma is" (DC, IDI 4, health policymaker)*

248 Participants believed good communication with parents would help school staff to
249 support a child's self-management. Prior agreement on emergency asthma school
250 plans could help boost teachers' confidence in dealing with an attack and protect them
251 from being blamed. A supporting letter from a healthcare professional regarding a
252 child's asthma condition and individual asthma action plan was suggested as another
253 way to provide the information to schools. A few healthcare professionals viewed
254 schools as a platform to bridge communication with parents who did not bring their
255 child for routine reviews.

> *"We need cooperation from the parent... parents should inform a teacher that 'my child has asthma and need this treatment'. So, the teacher will tell the other teachers and take care of the child... If parents provided a black and white instruction, that is even better." (HG, IDI 6, teacher)*

256 ***Implementation challenges***

257 Teachers and members of the school health team tended to be negative about school
258 asthma programmes because of the workload (Table 3). Teachers were already
259 burdened with academic responsibilities and thought an intensive programme might
260 affect children's learning and general education. The school health team had similar

For peer review only - http://bmjopen.bmj.com/site/about/guidelines.xhtml

concerns and felt overwhelmed by their workload, having to provide vaccination, screenings, and general health education in all the schools in the district.

> *"Cannot, we have a lot of programmes. There is no time to allocate in our schedule. A programme like this, can only be done infrequently." (ZZ_FGD6, teacher)*

Teachers needed to recognise asthma symptoms/attacks and provide first aid asthma treatment, but the parents of children with asthma needed to take responsibility for self-management of their children. Parental participation in the programme was thus considered crucial but parents' attendance at school health activities was often very poor either because of work commitments or because they perceived that the health programme was not important for them.

> *"The lower education group... maybe they are not financially capable, care less about health, maybe work is a priority. So, if we invite parents to come, this group may not have time to attend." (FF, FGD 2, teacher)*

The target groups for the school asthma programme have different needs and capabilities. Primary schools covered a wide age range (7-12years) and the teachers' experience was that lessons needed to be customised to the child's educational level. Some teachers suggested the programme should be delivered in the native language of the children (including Tamil (major language spoken by Indians in Malaysia) or Chinese as opposed to just the national language) for better understanding.

> *"JK: English, Tamil can.*
> *FG: But if it is in Tamil, they will understand better, because it is their native language.*
> *HI: but some lower and upper primary can understand Malay." (FGD 7, teachers)*

Implementation enablers

Participants suggested simple, engaging, and tailored approaches catering for busy schedules, and different individual needs and capabilities (Table 3). The delivery ought to be fun and appealing to children and adults. They suggested quizzes,

For peer review only - http://bmjopen.bmj.com/site/about/guidelines.xhtml

demonstrations, and the use of digital media such as videos to assist sessions delivery.

> *"We can use visual aids, we teach first. After that, we ask them to demonstrate. Something like a simulation, if this happened, what will you do?" (JI_IDI 7, teacher)*

Healthcare professionals considered good partnerships with schools as an essential enabler to promoting uptake of school asthma programmes. The school health team had a long-term relationship with the schools and suggested various approaches to building the partnership including involvement of the school administrators at the early stage of the programme development. A good partnership with schools also helped identify and address implementation issues, for example, teachers from Chinese/Indian schools volunteered to translate the intervention to help children who were not able to understand Malay.

> *"We have to hold a meeting with the headmaster and the teacher-in-charge of health… once the administrators are interested, they will suggest to you the teacher who will be in charge of the programme… So, you will work with the teacher to develop and run the programme." (NN, FGD 3, school health team)*

All participants proposed that healthcare professionals should deliver the intervention in schools, ideally as a multidisciplinary team including pharmacist and paediatrician. To increase parents' participation, teachers suggested wider community involvement such as parent-teacher associations, community organisations, and non-governmental organisations to help promote the intervention. Policymakers highlighted the need for introducing a national asthma school plan to improve asthma care of children during attacks.

> *"I think this matter will need a clear S.O.P. (standard operating procedure)… Although we have experience with our children, (but)for us to apply the same thing to someone's children, we are worried." (LK, IDI8, education policymaker)*

For peer review only - http://bmjopen.bmj.com/site/about/guidelines.xhtml

Discussion

Summary of findings

Asthma awareness and experience of managing asthma was limited among the school staff and, with no specific asthma school plan to provide guidance, treatment during asthma symptoms/emergencies could be inappropriate or delayed. School health circulars were not clear about the teachers' health role in schools. To improve this, participants suggested a school asthma programme which focused on introducing an asthma school plan and improving awareness about asthma amongst children, their parents and teachers. Communication between healthcare professionals, parents and the school also needed improvement. The participants anticipated that heavy workload, poor parental participation and diverse individual capabilities and needs as challenges to implementing school asthma programme. Tailored approaches, good partnership with school and multi-organisation involvement were discussed as enablers to develop and deliver the programme.

Study findings in comparison with other studies

Our interviewees suggested a school asthma programme involving parents and teachers, that focussed on asthma awareness, and an asthma school plan for use in attacks. Similar community-wide programmes have been implemented in other contexts.[28 29] Education for staff and children could correct common misperceptions about asthma and asthma medication and involvement of school health teams could improve accessibility of healthcare for children with asthma.[18 30-32] School asthma programmes offer the opportunity to focus self-management education on children and involve children's social network to support their self-management skills and practices.[33 34]

For peer review only - http://bmjopen.bmj.com/site/about/guidelines.xhtml

321 The lack of awareness among school staff about which students had asthma,
322 combined with poor understanding of symptoms and first aid treatment leads to
323 hesitancy and delay in taking action risking deterioration or even death.[35 36]
324 Delineating the health roles of teachers in an asthma school plan and providing
325 adequate training for school staff could improve their confidence and prevent
326 treatment delay.[15 37] However, such strategies rely on parents providing asthma
327 reliever medication for use in school; without this, school staff will have to call parents
328 or get immediate medical help.[35 38] School asthma guidelines in Australia, Canada, the
329 United Kingdom and the United States encourage inhalers to be labelled and kept with
330 the child or the class teacher in primary schools without a school nurse.[7 39-41] These
331 guidelines also recommend that schools have an emergency asthma kit to include a
332 reliever inhaler, spacer and agreed information on when and how to use it.[7-9 40]
333 Establishing immediate access to an emergency reliever inhaler in schools required
334 high level policy changes and detailed guidance for using first aid asthma treatment.[40
335 42]
336

337 Highlighting the importance of the programmes to stakeholders particularly
338 policymakers could ensure successful implementation of school asthma programmes.
339 Policy supporting improvement in school asthma management is a powerful tool in
340 prioritising and providing resources to assist implementation. A good partnership with
341 schools throughout the development of asthma school plans and programmes is
342 crucial to identify challenges and enablers for successful implementation.[43 44] The
343 presence of school plans does not guarantee teachers are equipped with the skills to
344 manage asthma symptoms/attacks.[45] Teachers need to receive education and training
345 in use of reliever treatment to equip them to handle asthma events in school.[45 46]

For peer review only - http://bmjopen.bmj.com/site/about/guidelines.xhtml

Tailoring designs according to the needs and capabilities of individuals and schools could improve the uptake and mitigate the challenges of the programmes.[18 47] Involving the wider community, e.g. parent and teacher association, health care providers and social services may support delivery and sustainability of school asthma programmes.[18 48 49]

Study strengths and limitations

This is one of a few studies on school asthma programmes conducted in low-middle income countries. Flexible time, venue and data collection methods facilitated recruitment of a study population with various backgrounds which contributed to the diverse views of participants. Memo, field notes and team discussion aided analysis and multidisciplinary team discussions informed interpretation.

Some loss of meaning may have occurred during the translation of Malay quotes; however, we analysed the data in the source language and checked the semantic and context equivalence for translated quotes. Thematic analysis may reduce the interpretive details of each participant' as the coding process focuses on the content, and less on the processes and phenomenon within individuals. However, we used a pragmatic, inductive approach with constant comparison of data and discussions to inform our understanding of the views expressed. Participants with less experience on the topic may have contributed less to the focus groups though they were provided with opportunities to provide their views.

Conclusions

A school asthma programme could improve the care and support for children with asthma. Aligned with the recommendations of a review of WHO school health

For peer review only

372 initiative[2], the participants in our study highlighted the importance of tailored
373 approaches, a good partnership with schools and engaging the wider community at an
374 early stage of developing a school asthma programme.

For peer review only

For peer review only - http://bmjopen.bmj.com/site/about/guidelines.xhtml

Ethics approval

We obtained ethical approval from the Malaysian National Medical Research and Ethics Committee (NMRR-18-3136-44042)

Data Availability

Due to ethical concerns, supporting data cannot be made openly available and are stored under restricted access in the University of Edinburgh DataVault. Further information about the data and conditions for access are available via the University of Edinburgh Research Explorer [we are in the process of depositing the data and the DOI will be provided here]. Additional non-disclosive details relating to other aspects of the data are openly available from the University of Edinburgh DataShare repository at https://datashare.ed.ac.uk/handle/10283/3860

Acknowledgements

We would like to acknowledge all the participants who voluntarily participated in this research and the local authorities for their support during the study. The RESPIRE collaboration comprises the UK Grant holders, partners and research teams as listed on the RESPIRE website (www.ed.ac.uk/usher/respire), including Nik Sherina Hanafi and Norita Binti Hussein.

Funding

This research is funded by NIHR Global Health Research 16/136/109. This research was commissioned by the UK National Institute for Health Research (NIHR) Global Health Research Unit on Respiratory Health (RESPIRE), using UK Aid from the UK Government. The views expressed in this publication are those of the author(s) and not necessarily those of the NIHR or the UK Department of Health and Social Care.

Competing Interest

None declared.

Author contributions

All authors contributed to study conception. SNR performed the data collection, checking and coded the transcripts. All authors contributed to data analysis and interpretation of data. SNR drafted the manuscript and all authors provided critical revisions and editing of the manuscript.

For peer review only

Table 1: Demographic characteristics of participants (N=52)

Demographic	n (years (mean))
Age range: years (mean)	23-58 (42)
Female	46
Male	6
Working experience: years (mean)	1-35 (17)
Education status	
Primary/Secondary	0
Tertiary	52
Employment background	
Healthcare professional	9
School staff	40
Policymaker	2
Teacher's professional society	1
Ethnicity	
Malay	20
Chinese	18
Indian	14
Background of participants	
Personal history of asthma	8
Family history of asthma	11
Certified healthcare professional	13

Table 2: Additional quotes in themes illustrating problems

**Variable asthma awareness**
"As for me, I would observe them, cool them down since I don't have any experience. Basically, I lacked the knowledge on how to treat asthma. So, that's the only way I can help." (QQ, FGD 4, teacher)
"At night if I cough, I will take the blue inhaler and usually, if I use the inhaler and it doesn't work… I will go to the emergency department to take nebuliser." (TT, FGD 4, school staff with asthma)
**Reliance on generic medical school plans**
"If the parents contacted me and said that my child can use this and know how to use this, I will tell the parents that it is the parents' and the child's responsibility to use the medication... You brought it to school and use it on your own. If a child has a more serious condition, we will allow the parents to enter the school to give medication according to the timing." (JJ, FGD 2, administrative staff)
"We are only afraid that something worse can happen. For example, we give medication, suddenly the child's condition gets worse." (FE, IDI 5, teacher)
**No communicated individual asthma action plan**
"I have a student with asthma, her mother said she has an inhaler, but she does not bring it to school. I've never seen it." (AA, FGD 1, teacher)

For peer review only

Table 3: Additional quotes illustrating themes for solutions, challenges and enablers.

**Suggested solutions**
"For improvement, we should ask parents to provide the asthma book together with their immunisation records. So, we can monitor their asthma appointment." (LL, FGD 3, school health team)
**Implementation challenges**
"We have many programmes, like the health-promoting programme, vaccination programme, the teacher also requested us to finish as soon as possible because they had to go through their education programme." (ZA, IDI 2, school health team)
**Implementation enablers**
"It has to be concise and simple. I think half an hour to an hour is enough. More than that, they (children) won't be able to concentrate." (YY, FGD 6, teacher)
"ZZ/AB: Malay can understand.<br>YY: Even if they cannot understand, a teacher will help to explain in Tamil. No problem." (FGD 6, teachers)
"We have a parent and community organisation, and the school parent and teacher organisation who can help to promote the programme. Parents can know about the programme from them, and they can come." (HG, IDI 6, teacher)

For peer review only

References

1. World Health Organisation. Global School Health Inititative Geneva, Switzerland2020 [Available from: https://www.who.int/school_youth_health/gshi/en/.
2. World Health Organization. Global School Health Initiatives: achieving health and education outcomes. Report of a meeting, Bangkok,Thailand, 23–25 November 2015. . Geneva, Switzerland, 2017.
3. Levinson J, Kohl K, Baltag V, et al. Investigating the effectiveness of school health services delivered by a health provider: A systematic review of systematic reviews. *PLoS One* 2019;14(6):e0212603. doi: 10.1371/journal.pone.0212603 [published Online First: 2019/06/13]
4. Global Asthma Network. The Global Asthma Report. Auckland, New Zealand, 2018.
5. Global Initiative for Asthma. Global Strategy for Asthma Management and Prevention (updated 2020), 2020.
6. Scottish Intercollegiate Guidelines Network/British Thoracic Society. SIGN 158 British guideline on the management of asthma. London: Scottish Intercollegiate Guidelines Network/British Thoracic Society, 2019.
7. Asthma Australia. Asthma Guidelines for Queenlands schools Australia: Asthma Australia, 2019.
8. Healthy London Partnership. London schools' guide for the care of children and young people with asthma: Pre-school, primary and secondary school years: London, 2016.
9. U.S. Department of Health and Human Services. Managing Asthma: A guide for schools: National Institutes of Health, 2014.
10. Piot P, Caldwell A, Lamptey P, et al. Addressing the growing burden of non-communicable disease by leveraging lessons from infectious disease management. *J Glob Health* 2016;6(1):010304-04. doi: 10.7189/jogh.06.010304
11. Harris K, Kneale D, Lasserson TJ, et al. School ‐ based self ‐ management interventions for asthma in children and adolescents: a mixed methods systematic review. *Cochrane Database of Systematic Reviews* 2019(1) doi: 10.1002/14651858.CD011651.pub2
12. Walter H, Sadeque-Iqbal F, Ulysse R, et al. Effectiveness of school-based family asthma educational programs on quality of life and asthma exacerbations in asthmatic children aged five to 18: a systematic review. *JBI Database of Systematic Reviews and Implementation Reports* 2016;14(11):113-38.
13. Isik E, Fredland NM, Freysteinson WM. School and Community-based Nurse-led Asthma Interventions for School-aged Children and Their Parents: A Systematic Literature Review. *Journal of Pediatric Nursing: Nursing Care of Children and Families* 2019;44:107-14. doi: 10.1016/j.pedn.2018.11.007
14. Al Aloola NA, Naik-Panvelkar P, Nissen L, et al. Asthma interventions in primary schools – a review. *Journal of Asthma* 2014;51(8):779-98. doi: 10.3109/02770903.2014.914534
15. Kew KM, Carr R, Donovan T, et al. Asthma education for school staff. *The Cochrane database of systematic reviews* 2017;4:Cd012255. doi: 10.1002/14651858.CD012255.pub2 [published Online First: 2017/04/13]
16. Craig P, Dieppe P, Macintyre S, et al. Developing and evaluating complex interventions: the new Medical Research Council guidance. *Int J Nurs Stud* 2013;50(5):587-92. doi: 10.1016/j.ijnurstu.2012.09.010

For peer review only - http://bmjopen.bmj.com/site/about/guidelines.xhtml

17. Wight D, Wimbush E, Jepson R, et al. Six steps in quality intervention development (6SQuID). *Journal of epidemiology and community health* 2016;70(5):520-5. doi: 10.1136/jech-2015-205952 [published Online First: 2015/11/18]
18. Cicutto L, Gleason M, Haas-Howard C, et al. Building Bridges for Asthma Care Program: A School-Centered Program Connecting Schools, Families, and Community Health-Care Providers. *The Journal of school nursing : the official publication of the National Association of School Nurses* 2018:1059840518805824. doi: 10.1177/1059840518805824 [published Online First: 2018/10/20]
19. Ramdzan SN, Pinnock H, Liew SM, et al. Perceptions of complementary/alternative medicine use and influence on evidence-based asthma medicine adherence in Malaysian children. *NPJ Prim Care Respir Med* 2019;29(1):5. doi: 10.1038/s41533-019-0118-x [published Online First: 2019/02/26]
20. Ramdzan SN, Khoo EM, Liew SM, et al. How young children learn independent asthma self-management: a qualitative study in Malaysia. *Archives of Disease in Childhood* 2020;105(9):819. doi: 10.1136/archdischild-2019-318127
21. Malaysia Education Statistics. Quick Facts 2018. Malaysia: Educational Planning and Research Division, Ministry of Education Malaysia, 2018.
22. Kementerian Kesihatan Malaysia. Garis Panduan Perkhidmatan Kesihatan Sekolah. Malaysia, 2013.
23. Braun V, Clarke V. Succesful Qualitative Research: a practical guide for beginners. London: SAGE Publication Ltd 2013.
24. Silverman D. Doing Qualitative Research. 5th edition ed. London, United Kingdom: SAGE Publication Limited 2018.
25. Farre A, Wood V, McDonagh JE, et al. Health professionals' and managers' definitions of developmentally appropriate healthcare for young people: conceptual dimensions and embedded controversies. *Arch Dis Child* 2016;101(7):628. doi: 10.1136/archdischild-2015-309473
26. Borrell-Carrió F, Suchman AL, Epstein RM. The Biopsychosocial Model 25 Years Later: Principles, Practice, and Scientific Inquiry. *The Annals of Family Medicine* 2004;2(6):576-82. doi: 10.1370/afm.245
27. Grbich C. Qualitative Data Analysis: An introduction. Second ed. United Kingdom: SAGE Publication 2013.
28. Lenney W, Bush A, Fitzgerald DA, et al. Improving the global diagnosis and management of asthma in children. *Thorax* 2018;73(7):662. doi: 10.1136/thoraxjnl-2018-211626
29. Szefler SJ, Fitzgerald DA, Adachi Y, et al. A worldwide charter for all children with asthma. *Pediatric Pulmonology* 2020;55(5):1282-92. doi: 10.1002/ppul.24713
30. Suwannakeeree P, Deerojanawong J, Prapphal N. School-Based Educational Interventions Can Significantly Improve Health Outcomes in Children with Asthma. *Journal of the Medical Association of Thailand = Chotmaihet thangphaet* 2016;99(2):166-74. [published Online First: 2016/06/03]
31. Clark NM, Gong M, Kaciroti N, et al. A trial of asthma self-management in Beijing schools. *Chronic illness* 2005;1(1):31-8. doi: 10.1177/17423953050010010101 [published Online First: 2006/12/02]
32. Carvalho Coelho AC, Barretto Cardoso LS, de Souza-Machado C, et al. The Impacts of Educational Asthma Interventions in Schools: A Systematic Review of the Literature. *Can Respir J* 2016:8476206. doi: 10.1155/2016/8476206

For peer review only - http://bmjopen.bmj.com/site/about/guidelines.xhtml

33. Szefler SJ, Cloutier MM, Villarreal M, et al. Building Bridges for Asthma Care: Reducing school absence for inner-city children with health disparities. *J Allergy Clin Immunol* 2019;143(2):746-54.e2. doi: 10.1016/j.jaci.2018.05.041 [published Online First: 2018/07/29]

34. Lemanske RF, Jr., Kakumanu S, Shanovich K, et al. Creation and implementation of SAMPRO™: A school-based asthma management program. *The Journal of allergy and clinical immunology* 2016;138(3):711-23. doi: 10.1016/j.jaci.2016.06.015

35. Greiling AK, Boss LP, Wheeler LS. A preliminary investigation of asthma mortality in schools. *The Journal of school health* 2005;75(8):286-90. doi: 10.1111/j.1746-1561.2005.00039.x [published Online First: 2005/09/24]

36. Volerman A, Kim TY, Sridharan G, et al. A Mixed-methods Study Examining Inhaler Carry and Use among Children at School. *J Asthma* 2020;57(10):1071-82. doi: 10.1080/02770903.2019.1640729 [published Online First: 2019/07/06]

37. Murphy KR, Hopp RJ, Kittelson EB, et al. Life-threatening asthma and anaphylaxis in schools: a treatment model for school-based programs. *Ann Allergy Asthma Immunol* 2006;96(3):398-405. doi: 10.1016/s1081-1206(10)60906-4 [published Online First: 2006/04/07]

38. Gerald JK, Stroupe N, McClure LA, et al. Availability of Asthma Quick Relief Medication in Five Alabama School Systems. *Pediatric allergy, immunology, and pulmonology* 2012;25(1):11-16. doi: 10.1089/ped.2011.0118

39. Association ASH. Policy Statement—Guidance for the Administration of Medication in School. *Pediatrics* 2009;124(4):1244. doi: 10.1542/peds.2009-1953

40. Department of Health. Guidance on the use of emergency salbutamol inhalers in schools. Storage and care of the inhaler. United Kingdom, 2015.

41. OPHEA Health schools and Healthy Communities. Creating Asthma friendly schools. Canada, 2015.

42. Toups MM, Press VG, Volerman A. National Analysis of State Health Policies on Students' Right to Self-Carry and Self-Administer Asthma Inhalers at School. *The Journal of school health* 2018;88(10):776-84. doi: 10.1111/josh.12681

43. Cicutto L, Gleason M, Szefler SJ. Establishing school-centered asthma programs. *J Allergy Clin Immunol* 2014;134(6):1223-30. doi: 10.1016/j.jaci.2014.10.004 [published Online First: 2014/12/09]

44. Kakumanu S, Lemanske RF, Jr. Asthma in Schools: How School-Based Partnerships Improve Pediatric Asthma Care. *Immunol Allergy Clin North Am* 2019;39(2):271-81. doi: 10.1016/j.iac.2018.12.011 [published Online First: 2019/04/08]

45. Jaramillo Y, Reznik M. Do United States' teachers know and adhere to the national guidelines on asthma management in the classroom? A systematic review. *TheScientificWorldJournal* 2015;2015:624828. doi: 10.1155/2015/624828 [published Online First: 2015/03/03]

46. Cain A, Reznik M. Asthma management in New York City schools: A classroom teacher perspective. *J Asthma* 2016;53(7):744-50. doi: 10.3109/02770903.2015.1135946 [published Online First: 2016/04/01]

47. Brigden A, Parslow RM, Linney C, et al. How are behavioural interventions delivered to children (5-11 years old): a systematic mapping review. *BMJ Paediatr Open* 2019;3(1):e000543. doi: 10.1136/bmjpo-2019-000543 [published Online First: 2020/01/08]

For peer review only - http://bmjopen.bmj.com/site/about/guidelines.xhtml

48. McCabe EM, Kaskoun JR, Murphy EL, et al. An Evidence-Based Framework for Implementation of a School–Community Partnership. *NASN School Nurse* 2020:1942602X20964765. doi: 10.1177/1942602X20964765
49. Clark NM, Lachance L, Doctor LJ, et al. Policy and system change and community coalitions: outcomes from allies against asthma. *American journal of public health* 2010;100(5):904-12. doi: 10.2105/AJPH.2009.180869 [published Online First: 2010/03/18]

Figure legend

Figure 1: Themes and subthemes of participants' views of asthma and school-based asthma intervention.

For peer review only

For peer review only - http://bmjopen.bmj.com/site/about/guidelines.xhtml

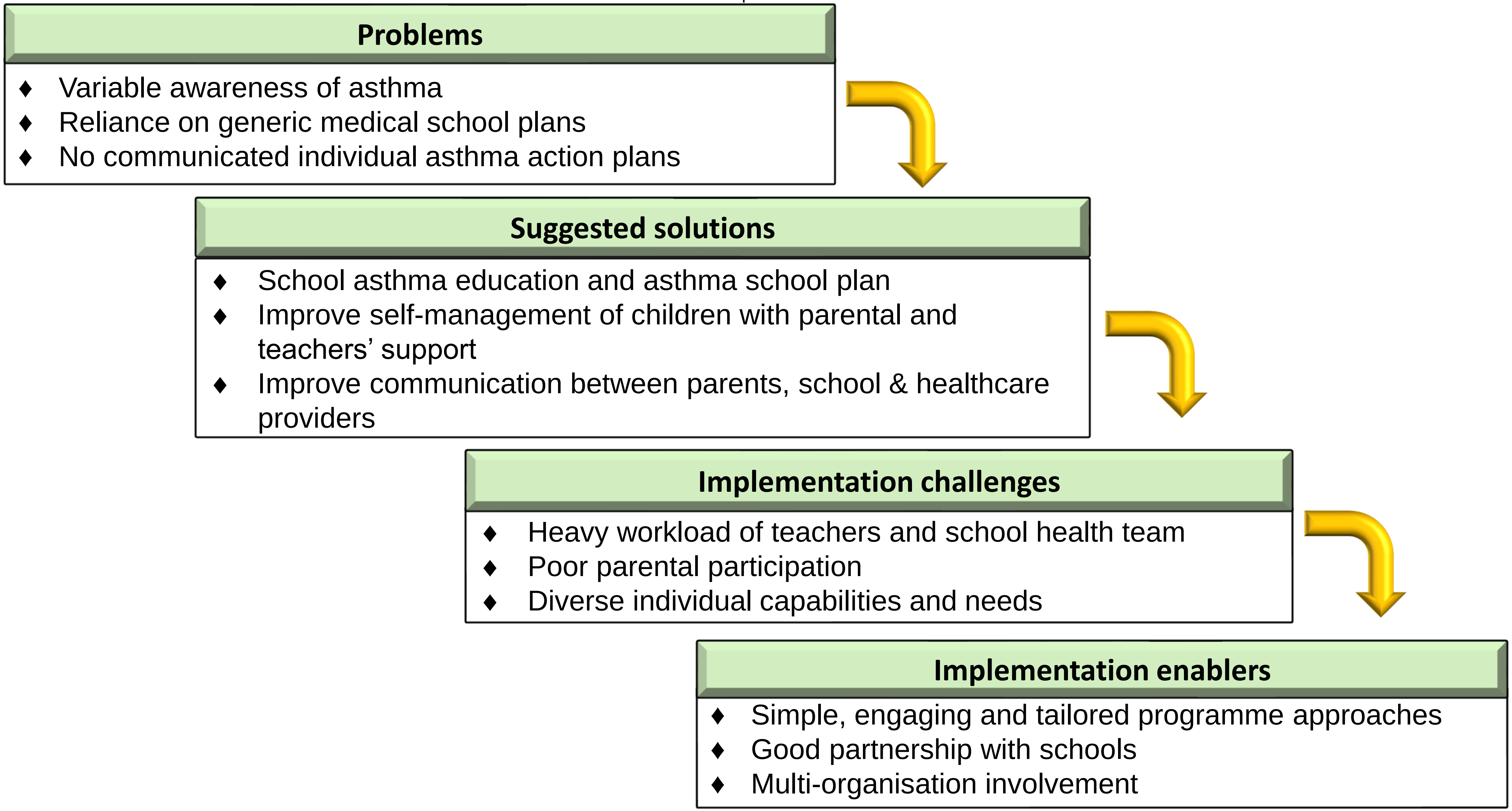

Figure 1 : Themes and subthemes of participants' views of asthma and school-based asthma intervention.

For peer review only - http://bmjopen.bmj.com/site/about/guidelines.xhtml

Borang soal selidik latar belakang
Socio-demographic questionnaire

Latar belakang
Background

Umur*(Age)* : ___________

Jantina*(Gender)* : ___________

Bangsa*(Ethnicity)* : ___________

Status perkahwinan *(Marital status)*:

Bujang*(Single)*/Bercerai *(Divorced)*/Berkahwin*(Married)*

Tahap pendidikan tertinggi *(Highest educational level achieved):*

Rendah*(Primary)*/Menengah *(Secondary)*/Pengajian Tinggi *(Tertiary)*

Pekerjaan *(Occupation)* : ____________________

Pengalaman berkerja (work experience) : ………… /tahun(year)

Anggaran pendapatan isi rumah

(Estimated family income): RM ________ /bulan(month)

Informasi mengenai asma
Information about asthma

Adakah anda menghidap asma *(Do you have asthma)?*

Ya *(yes)*/ Tidak *(No)*

Adakah ahli keluarga menghidap asma (Anyone in your family has asthma)?

Ya *(yes)*/ Tidak *(No)*

Jika Ya, sila nyatakan (If yes, (please specify):

____

For peer review only

Childhood Asthma
Participant Interview Topic Guide (school staff)

Preamble:

- **Ice-breaking and explain that there is no right or wrong answer**
- **Explain need to get consent for the interview and audio-recording. Obtain consents.**
- **Explain that the participant does not have to answer if he or she does not wish to do so**

Question	Prompts
**Asthma experience**	
Tell us your experience in dealing with children with asthma?	Your child? Your student?
What do you know about asthma?	Experience? Reading? Told by someone?
How do you know if a child's asthma is good or badly controlled?	Able to play? Easy breathing? No cough? How do you know about this?
How does a child's asthma disturb/not disturb he/her? How?<br>How does a child's asthma disturbs/not disturb you? How?	Activities affected e.g. attending school?<br>*Pendidikan Jasmani dan kesihatan* (PJK)/sports?
In your opinion, how does having asthma makes a child feel?	Emotionally-friends isolation/bully? Shy to use medication?
**Asthma treatment**	
Do you know of any asthma treatment?	Pills? MDI? Spacer? Aerochamber?
How about complementary and alternative medicine for asthma?	
**Asthma management at school**	
What do you do when a student has an asthma attack?	Inhaler? Go to the hospital/clinic? Call parents?
How do you recognise a severe asthma attack?<br>What would you do if this happened?	Breathlessness? Unable to speak?
Is there anything else you would do?	Concern on the administration of medication eg MDI to student(s)? Rules or legislation?
Is there any rules/limitation for students with asthma at school?	Sports? Physical activity? Extracurricular activities?
Is there any asthma education or campaign at school?	How is it delivered? By whom? To who?
What do you think about asthma education at school? Is it necessary?	Feasible? How best to deliver? Parents and school staffs involvement? How often?
What do you think if school staffs are allowed to deliver/supervise the use of asthma medication e.g inhaler at school?	Good? Not good? Concerns?

For peer review only

Suggestions/ideas for intervention?<br><br>Any suggestion on how to improve asthma management at school?	Others? Timing – during or after school?
**Health belief about asthma**<br><br>Why do you think a child has asthma?<br><br>What do you think a child can do to prevent asthma?	 <br>Inherited? Infection? Environment?<br><br>Avoidance of food? Medication? Sports?
**Source of information**<br><br>Where and from whom did you get information about asthma?<br><br>Anything else that you want to share about childhood asthma?	 <br>Clinic? Family? Friends? Internet? How good is the information?

For peer review only

Childhood Asthma (CuT-AsthMa)
Participant Interview Topic Guide (School Health Team)

Preamble:
- **Ice-breaking and explain that there is no right or wrong answer**
- **Explain the need to get consent for the interview and audio-recording. Obtain consents.**
- **Explain that the participant does not have to answer if he or she does not wish to do so**

Question	Prompts
**Asthma experience**	
Tell us your experience about dealing with children with asthma?	A student? A patient? Your own child?
How do you know that they have asthma?	Symptoms e.g cough, wheezing? Informed by parents?
How do you know if the child's asthma is good or badly controlled?	Able to play? Easy breathing? No cough? Where do you know about this?
How does a student's asthma disturb/not disturb him/her?	Physically? Activities affected at school e.g PJK, absent from school?
How does a student's asthma disturbs/not disturb you? How?	
In your opinion, how does having asthma makes a student feel?	Emotionally-friends isolation/bully? Shy to use medication?
**Asthma treatment**	
Do you know of any asthma treatment?	Pills? MDI? Spacer? Aerochamber?
How about complementary and alternative medication for asthma?	
**Asthma management at school**	
What do you do when a student has asthma attack?	Inhaler? Go to hospital/clinic? Asthma action plan? How do you know this?
How do you recognise a severe asthma attack? What would you do if this happened?	Breathlessness? Unable to speak?
Is there anything else you would do?	
	Sports? Physical activity? Extracurricular activities?

For peer review only

Is there any rules/limitation for students with asthma at school?<br><br>Is there any asthma education or campaign at school?<br><br>What do you think of asthma education at school? Is it necessary? Is it feasible?<br><br>What is your opinion on teachers delivering/supervising the use of asthma medication e.g inhaler at school?<br><br>Any suggestion on how to improve asthma management at school?	How is it delivered? By whom? To who?<br><br>Feasible? How best to deliver? Parents and teachers involvement? How often?<br><br>Good? Not good? Any worries?
**Health belief about asthma**<br><br>Why do you think a child has asthma?<br><br>What do you think a child can do to prevent asthma?	Inherited? Infection? Environment?<br><br>Avoidance of food? Medication? Sports?
**Source of information**<br><br>Where and from whom did you get information about asthma?<br><br>Anything else that you want to share about asthma among children?	Clinic? Family? Friends? Internet? How good is the information?

For peer review only

Childhood Asthma (CuT-AsthMa)
Participant focus group topic guide (Healthcare professionals/policymakers)
(Interviews, which will be offered as an option to suit the convenience/preference of the participant, will follow the same topic guide)

Preamble:

- **Ice-breaking and explain that there is no right or wrong answer**
- **Explain the need to get consent for the focus group/interview and audio-recording. Obtain consents.**
- **Explain that the participant does not have to answer if he or she does not wish to do so**

Question	Prompts
**Asthma experience**<br><br>Tell us about your experience in dealing with children with asthma?<br><br>How do you know that they have asthma?<br><br>How do you know if the child's asthma is good or badly controlled?<br><br>How does a student's asthma disturb/not disturb him/her? How does a student's asthma disturbs/not disturb you? How?<br><br>In your opinion, how does having asthma makes a student feel?	 <br>A student? A patient? Your child?<br><br>Symptoms e.g cough, wheezing? Informed by parents?<br><br>Able to play? Easy breathing? No cough? Where do you know about this?<br><br>Physically? Activities affected at school e.g PJK, absent from school?<br><br>Emotionally-friends isolation/bully? Shy to use medication?
**Asthma treatment**<br><br>Do you know of any asthma treatment?<br><br>How about complementary and alternative medicine for asthma?	 <br>Pills? MDI? Spacer? Aerochamber?
**Asthma management at school**<br><br>What do you do when a student has an asthma attack?<br><br>How do you recognise a severe asthma attack? What would you do if this happened?<br><br>Is there anything else you would do?<br><br>Is there any rules/limitation for students with asthma at school?<br><br>Is there any asthma education or campaign at school?	 <br>Inhaler? Go to the hospital/clinic? Asthma action plan? How do you know this?<br><br>Breathlessness? Unable to speak?<br><br>Sports? Physical activity? Extracurricular activities?<br><br>How is it delivered? By whom? To who?
What do you think of asthma education at school? Is it necessary? Is it feasible?	Feasible? How best to deliver? Parents and teachers involvement? How often?

For peer review only - http://bmjopen.bmj.com/site/about/guidelines.xhtml

What is your opinion on teachers delivering/supervising the use of asthma medication e.g inhaler at school?<br><br>Any suggestion on how to improve asthma management at school?	Good? Not good? Any worries?
**Health belief about asthma**<br><br>Why do you think a child has asthma?<br><br>What do you think a child can do to prevent asthma?	 <br>Inherited? Infection? Environment?<br><br>Avoidance of food? Medication? Sports?
**Source of information**<br><br>Where and from whom did you get information about asthma?<br><br>Anything else that you want to share about asthma among children?	 <br>Clinic? Family? Friends? Internet? How good is the information?

For peer review only

Standards for Reporting Qualitative Research (SRQR)*

http://www.equator-network.org/reporting-guidelines/srqr/

Title and abstract

	Page/line no(s).
**Title** - Concise description of the nature and topic of the study Identifying the study as qualitative or indicating the approach (e.g., ethnography, grounded theory) or data collection methods (e.g., interview, focus group) is recommended	1/1-3
**Abstract** - Summary of key elements of the study using the abstract format of the intended publication; typically includes background, purpose, methods, results, and conclusions	2-3/27-57

Introduction

**Problem formulation** - Description and significance of the problem/phenomenon studied; review of relevant theory and empirical work; problem statement	4-5/71-95
**Purpose or research question** - Purpose of the study and specific objectives or questions	5/95-99

Methods

**Qualitative approach and research paradigm** - Qualitative approach (e.g., ethnography, grounded theory, case study, phenomenology, narrative research) and guiding theory if appropriate; identifying the research paradigm (e.g., postpositivist, constructivist/ interpretivist) is also recommended; rationale**	5-6/102-120
**Researcher characteristics and reflexivity** - Researchers' characteristics that may influence the research, including personal attributes, qualifications/experience, relationship with participants, assumptions, and/or presuppositions; potential or actual interaction between researchers' characteristics and the research questions, approach, methods, results, and/or transferability	7-8/154-162
**Context** - Setting/site and salient contextual factors; rationale**	6/111-114
**Sampling strategy** - How and why research participants, documents, or events were selected; criteria for deciding when no further sampling was necessary (e.g., sampling saturation); rationale**	6/114-120
**Ethical issues pertaining to human subjects** - Documentation of approval by an appropriate ethics review board and participant consent, or explanation for lack thereof; other confidentiality and data security issues	5/102-107
**Data collection methods** - Types of data collected; details of data collection procedures including (as appropriate) start and stop dates of data collection and analysis, iterative process, triangulation of sources/methods, and modification of procedures in response to evolving study findings; rationale**	6-7/123-135

For peer review only - http://bmjopen.bmj.com/site/about/guidelines.xhtml

**Data collection instruments and technologies** - Description of instruments (e.g., interview guides, questionnaires) and devices (e.g., audio recorders) used for data collection; if/how the instrument(s) changed over the course of the study	6-7/125-135
**Units of study** - Number and relevant characteristics of participants, documents, or events included in the study; level of participation (could be reported in results)	8/172-179 (Table 1)
**Data processing** - Methods for processing data prior to and during analysis, including transcription, data entry, data management and security, verification of data integrity, data coding, and anonymization/de-identification of excerpts	7/138-142
**Data analysis** - Process by which inferences, themes, etc., were identified and developed, including the researchers involved in data analysis; usually references a specific paradigm or approach; rationale**	7/145-151
**Techniques to enhance trustworthiness** - Techniques to enhance trustworthiness and credibility of data analysis (e.g., member checking, audit trail, triangulation); rationale**	7-8/158-162

Results/findings

**Synthesis and interpretation** - Main findings (e.g., interpretations, inferences, and themes); might include development of a theory or model, or integration with prior research or theory	9-14/172-295, Figure 1
**Links to empirical data** - Evidence (e.g., quotes, field notes, text excerpts, photographs) to substantiate analytic findings	9-14/quotes and Table 2-3

Discussion

**Integration with prior work, implications, transferability, and contribution(s) to the field -** Short summary of main findings; explanation of how findings and conclusions connect to, support, elaborate on, or challenge conclusions of earlier scholarship; discussion of scope of application/generalizability; identification of unique contribution(s) to scholarship in a discipline or field	15-18 /298-374
**Limitations** - Trustworthiness and limitations of findings	17/353-367

Other

**Conflicts of interest** - Potential sources of influence or perceived influence on study conduct and conclusions; how these were managed	19/399
**Funding** - Sources of funding and other support; role of funders in data collection, interpretation, and reporting	19/393-396

*The authors created the SRQR by searching the literature to identify guidelines, reporting standards, and critical appraisal criteria for qualitative research; reviewing the reference lists of retrieved sources; and contacting experts to gain feedback. The SRQR aims to improve the transparency of all aspects of qualitative research by providing clear standards for reporting qualitative research.

For peer review only - http://bmjopen.bmj.com/site/about/guidelines.xhtml

**The rationale should briefly discuss the justification for choosing that theory, approach, method, or technique rather than other options available, the assumptions and limitations implicit in those choices, and how those choices influence study conclusions and transferability. As appropriate, the rationale for several items might be discussed together.

Reference:

O'Brien BC, Harris IB, Beckman TJ, Reed DA, Cook DA. **Standards for reporting qualitative research: a synthesis of recommendations.** *Academic Medicine*, Vol. 89, No. 9 / Sept 2014 DOI: 10.1097/ACM.0000000000000388

For peer review only

For peer review only - http://bmjopen.bmj.com/site/about/guidelines.xhtml

BMJ Open

Stakeholders' views of asthma and school-based asthma programme for primary school children: A qualitative study in Malaysia

Journal:	*BMJ Open*
Manuscript ID	bmjopen-2021-052058.R1
Article Type:	Original research
Date Submitted by the Author:	12-Oct-2021

Complete List of Authors:	Ramdzan, Siti Nurkamilla; Universiti Malaya, Department of Primary Care Medicine; The University of Edinburgh Usher Institute of Population Health Sciences and Informatics, NIHR Global Health Research Unit on Respiratory Health (RESPIRE)<br>Khoo, Ee Ming; Universiti Malaya, Department of Primary Care Medicine<br>Liew, Su May; Universiti Malaya, Department of Primary Care Medicine<br>Cunningham, Steven; The University of Edinburgh Usher Institute of Population Health Sciences and Informatics, NIHR Global Health Research Unit on Respiratory Health (RESPIRE)<br>Pinnock, Hilary; The University of Edinburgh Usher Institute of Population Health Sciences and Informatics, NIHR Global Health Research Unit on Respiratory Health (RESPIRE)

**Primary Subject Heading**:	Health services research
Secondary Subject Heading:	Paediatrics, Respiratory medicine, Public health
Keywords:	Asthma < THORACIC MEDICINE, Paediatric thoracic medicine < PAEDIATRICS, QUALITATIVE RESEARCH, Community child health < PAEDIATRICS, Change management < HEALTH SERVICES ADMINISTRATION & MANAGEMENT

SCHOLARONE™ Manuscripts

I, the Submitting Author has the right to grant and does grant on behalf of all authors of the Work (as defined in the below author licence), an exclusive licence and/or a non-exclusive licence for contributions from authors who are: i) UK Crown employees; ii) where BMJ has agreed a CC-BY licence shall apply, and/or iii) in accordance with the terms applicable for US Federal Government officers or employees acting as part of their official duties; on a worldwide, perpetual, irrevocable, royalty-free basis to BMJ Publishing Group Ltd ("BMJ") its licensees and where the relevant Journal is co-owned by BMJ to the co-owners of the Journal, to publish the Work in this journal and any other BMJ products and to exploit all rights, as set out in our licence.

The Submitting Author accepts and understands that any supply made under these terms is made by BMJ to the Submitting Author unless you are acting as an employee on behalf of your employer or a postgraduate student of an affiliated institution which is paying any applicable article publishing charge ("APC") for Open Access articles. Where the Submitting Author wishes to make the Work available on an Open Access basis (and intends to pay the relevant APC), the terms of reuse of such Open Access shall be governed by a Creative Commons licence – details of these licences and which Creative Commons licence will apply to this Work are set out in our licence referred to above.

Other than as permitted in any relevant BMJ Author's Self Archiving Policies, I confirm this Work has not been accepted for publication elsewhere, is not being considered for publication elsewhere and does not duplicate material already published. I confirm all authors consent to publication of this Work and authorise the granting of this licence.

For peer review only

Stakeholders' views of asthma and school-based asthma programme for primary school children: A qualitative study in Malaysia

Siti Nurkamilla Ramdzan, MMed[1,2], Ee Ming Khoo, MD[1], Su May Liew, DPhil[1], Steve Cunningham, PhD[2], Hilary Pinnock, MD*[2] on behalf of RESPIRE collaborators

[1]Department of Primary Care Medicine, Faculty of Medicine, University of Malaya, Malaysia

[2]NIHR Global Health Research Unit on Respiratory Health (RESPIRE), Usher Institute, University of Edinburgh, United Kingdom

Address for correspondence
*Professor Hilary Pinnock
NIHR Global Health Research Unit on Respiratory Health (RESPIRE),
Usher Institute,
The University of Edinburgh.
Doorway 3, Medical School, Teviot Place,
Edinburgh EH8 9AG, UK.
Tel: 0131 650 9474
Email: Hilary.Pinnock@ed.ac.uk

Main text word count: 3876 (excluding abstract, main headings, tables, references, and additional information, e.g. acknowledgement, funding, competing interest) Maximum: 4000 words

For peer review only

Abstract

Objective: The WHO Global School Health Initiative aimed to improve child and community health through health promotion programmes in schools, though most focus on preventing communicable disease. Despite WHO recommendations, no asthma programme is included in the Malaysian national school health service guideline. We aimed to explore the views of school staff, healthcare professionals and policymakers, about asthma and school asthma programmes for primary school children.

Design: A qualitative study using purposive sampling of participants to obtain diverse views. We conducted focus groups and individual interviews using piloted semi-structured topic guides. The focus groups and interviews were audio-recorded, transcribed verbatim and analysed using inductive thematic analysis. We completed data collection once data saturation was reached.

Setting: Stakeholders in education and health sectors in Malaysia.

Participants: Fifty-two participants (40 school staff, 9 healthcare professionals and 3 policymakers) contributed to nine focus groups and eleven individual interviews.

Results: School staff had limited awareness of asthma and first-aid asthma treatments. There was no guidance on asthma management in government schools, and teachers were unclear about their role in school children's health. These uncertainties led to delays in the treatment of asthma symptoms/attacks, and suggestions that an asthma education programme and a school plan would improve asthma care. Perceived challenges in conducting school health programmes included a busy school schedule and poor parental participation. A tailored asthma programme in partnerships with schools could facilitate the programme's adoption and implementation.

For peer review only

Conclusions: Identifying and addressing issues and challenges specific to the school and wider community could facilitate the delivery of a school asthma programme in line with the WHO School Health Initiative. Clarity over national policy on the roles and responsibilities of school staff could support implementation and guide appropriate and prompt response to asthma emergencies in schools.

Word count: 286 (Maximum: 300)

Keywords: school-based, asthma, complex intervention, low-middle income countries and health behaviour

For peer review only

Strengths and limitations of this study

- One of a few studies on school asthma programmes conducted in low-middle income countries.
- Various backgrounds contributed to the diverse views of participants.
- Some loss of meaning may have occurred during the translation of Malay quotes to English but the analysis was conducted in the source language.
- Participants with less experience on the topic may have contributed less to the focus groups though they were provided with opportunities to provide their views.

Introduction

The Global School Health Initiative launched by the World Health Organization (WHO) in 1995 aimed to improve child, adolescent and community health through health promotion programmes in schools.[1] Globally, the compulsory school years offer an 'easy entry point' for embedding healthy behaviours at a young age and, by extension, influencing families.[2] Health promotion programmes (e.g. de-worming, vaccination, water, sanitation, hygiene) have been successfully implemented in low-middle income countries (LMICs) through this initative.[2] Non-communicable diseases (NCDs) are an increasing challenge in all economies, though successful NCD programmes have typically been focussed in high-income countries.[2 3]

Asthma is the commonest NCD in children causing significant morbidity and mortality globally.[4] Asthma symptoms and attacks can occur unexpectedly, and schools need plans for responding appropriately and promptly to asthma symptoms.[5 6] Young children can spend up to half their waking hours in schools, and school staff could

For peer review only

support the development of asthma self-management skills in the students.[7-9] A school asthma programme offers an exemplar for school health NCD programmes and the WHO recommended asthma as an essential school health service in all countries.[3 10 11]

School-based asthma self-management interventions have been shown to improve asthma control, reduce school absenteeism and asthma exacerbations[12-15], though few were conducted in LMICs.[12 15 16] These programmes are complex interventions that need to be tailored to the target population[17 18], and adapted to the context.[19] In a review of the first two decades of school health initiatives, the WHO highlighted the importance of policy and organisation-level collaboration and engagement with parents, students and teachers.[2]

In Malaysia, an estimated 9% of children have asthma [20 21], at least half of whom are poorly controlled.[20 22] Despite this morbidity, the national school health service guideline does not include an asthma programme[23] , and a qualitative study conducted among primary school children with asthma and their parents revealed a perception of poor support for asthma care in schools.[24 25] Building on the findings of the qualitative study[24 25], we aimed to explore the views of school staff, healthcare professionals and policymakers about asthma and school-based asthma management to inform the development of an intervention on primary school asthma programme in Malaysia.

For peer review only - http://bmjopen.bmj.com/site/about/guidelines.xhtml

Methods

This qualitative study was conducted between May and December 2019 with ethical approval from the Malaysian National Medical Research and Ethics Committee (NMRR-18-3136-44042 (IIR)) and sponsorship from the Academic and Clinical Centre for Research and Development, University of Edinburgh (AC18113). We obtained permission from the schools, the Malaysian Ministry of Health and Ministry of Education to conduct the study. All participants provided their written informed consent.

Context and sampling

About 98% of Malaysia's primary education takes place in government schools under the Ministry of Education.[26] In these schools, there are no school nurses and the healthcare of school children is provided by the Ministry of Health in a form of a school health team comprised of family medicine specialists, medical officers and nurses, who provides general health screening and education at each district.[23] We conducted the study in the central region of Peninsular Malaysia and purposively sampled school staff (teachers and administrative staff) from five government schools (two Malay, two Chinese and one Indian), to represent the three main ethnic groups and diverse cultures in Malaysia.[26] We also purposively invited participants from a non-governmental organisation and a private school, healthcare professionals including the school health team and policymakers to obtain views from the wider education community.

For peer review only

Data collection

We arranged focus groups to enable peer interaction supplemented with individual interviews to accommodate the availability and preference of participants.[27 28] We collected basic socio-demographic data (Supplementary 1) and developed piloted semi-structured topic guides (Supplementary 2) based on the biopsychosocial model and previous literature to stimulate discussion.[24 25 29 30] The core topics addressed were asthma experience and awareness, current school asthma management practices and ideas for improvement. All the interviews and focus groups were conducted either in Malay (the national language of Malaysia) or English and moderated by SNR, a researcher who is proficient in both languages. We anticipated up to 50 participants and concluded data collection when there were no new themes emerging based on our core topics. The focus groups and interviews were audio-recorded, transcribed verbatim and analysed using Nvivo software version 11.

Language and translation

The transcripts were analysed in the source language to reduce loss of meaning with translation. Aided by the field notes, the Malay transcripts were translated to English by experienced bilingual translators, and SNR checked the translated transcripts for semantic and contextual equivalence. The translated transcripts were used for data sharing, e.g., the use of quotes to illustrate the findings in this report.

Data analysis

We used inductive thematic analysis to explore the views of the participants about asthma and school asthma management. It is a systematic but flexible approach that is often used to inform intervention development and evaluation.[27 31] SNR read the

For peer review only - http://bmjopen.bmj.com/site/about/guidelines.xhtml

transcripts multiple times for data familiarisation and developed initial codes according to the research question. Constant comparison and merging of initial codes allowed the development of sub-codes, sub-themes and themes, and we then constructed a schema to describe the relationship between themes. Memos, field notes and discussions with the research team aided data interpretation.

Reflexivity and interpretation

SNR who undertook data collection and coding, is an academic family physician with more than 15 years of working experience in Malaysia. At the beginning of the focus groups/interviews, SNR explained that the main purpose of the study was to improve the care of children with asthma. She emphasised her position as a researcher in understanding participants' views without judgements. The analysis and results were discussed with the multi-cultural team consisting of primary care physicians and a paediatrician from the UK and Malaysia, to ensure balanced interpretation. We used a pragmatic and constructivist approach but constantly reminded ourselves to be open to any possible emerging theory.

Patient and Public Involvement

We piloted the topic guides among teachers and health professionals when designing the study. They provided feedback on the approach and questions in the topic guides. We shared the preliminary findings of our study with some participants and other stakeholders in stakeholder engagement meetings.

Results

We recruited 52 participants consisting of 40 school staff (including three from a private school), nine healthcare professionals (five from the school health team), two

For peer review only

policymakers and a representative from the teachers' professional association. We conducted nine focus groups (up to six participants for each group; duration up to 40 minutes) and 11 individual interviews (duration up to 45 minutes). Table 1 shows the characteristics of the participants and Table 2 details the participants in each focus group. The participants' age ranged from 23 to 58 years and their working experience ranged 1 to 35 years. A quarter of them (14) had a personal history of asthma or a family member with asthma.

Table 1: Demographic characteristics of participants (N=52)

Demographic	n (years (mean))
Age range: years (mean)	23-58 (42)
Female	46
Male	6
Working experience: years (mean)	1-35 (17)
Education status	
Primary/Secondary	0
Tertiary	52
Employment background	
Healthcare professional	9
School staff	40
Policymaker	2
Teacher's professional society	1
Ethnicity	
Malay	20
Chinese	18
Indian	14
Background of participants	
Personal history of asthma	8
Family history of asthma	11
Certified healthcare professional	13

For peer review only

Table 2: Details of participants in each focus group.

Focus group	Participants
1	5 teachers
2	5 teachers
3	4 nurses and 1 nurse assistant
4	3 teachers and 1 clerk
5	4 teachers
6	6 teachers
7	3 teachers and 1 clerk
8	6 teachers
9	2 school nurses and 1 school administrator

For peer review only

Summary of findings

Figure 1 illustrates the inter-relationship of the themes and subthemes. Four themes were constructed to inform the development of a school asthma programme; the first highlighted the problems perceived by participants, the second reported the suggested solutions and the two following themes addressed the challenges and enablers.

Quotes describing the themes are provided in text boxes and additional quotes are provided in Tables 3 and 4.

Problems

Variable awareness of asthma

The awareness of asthma among the participants depended on their personal experience of having asthma or helping others to self-manage asthma, e.g., family members or students, and/or if they had received asthma training or education (Table

For peer review only - http://bmjopen.bmj.com/site/about/guidelines.xhtml

3). Participants with experience or training could describe symptoms and were familiar with the treatment for asthma symptoms/attacks. Other participants were less able to contribute to the discussion about asthma symptoms, treatment, and possible complications. They had several misconceptions, e.g., 'children with asthma cannot do physical activities', and ‘attacks do not occur suddenly in a well child'.

"Moderator: What happens if the kid has asthma, but he/she says that he/she can do it (physical activities)?
C2: We don’t let them do it.” (FGD2_SS, school staff)

E2: I think if the child is having asthma (symptoms), it's best for the parents to bring their child to see the doctor. Can an asthma attack suddenly occur when the child is well?
Moderator: Yes.
E2: I don't know about that.” (FGD2_SS, school staff)

Table 3: Additional quotes in themes illustrating problems

**Variable asthma awareness**
"As for me, I would observe them, cool them down since I don't have any experience. Basically, I lacked the knowledge on how to treat asthma. So, that's the only way I can help." (RO4_FGD4_SS, school staff)
"At night if I cough, I will take the blue inhaler and usually, if I use the inhaler and it doesn't work... I will go to the emergency department to take nebuliser." (A14_FGD 4_SS, school staff with asthma)
**Reliance on generic medical school plans**
"If the parents contacted me and said that my child can use this and know how to use this, I will tell the parents that it is the parents' and the child's responsibility to use the medication... You brought it to school and use it on your own. If a child has a more serious condition, we will allow the parents to enter the school to give medication according to the timing." (E2_FGD 2_SS, teacher)
"A5: Sometimes, when we give treatment to a student.<br>*C5: parents blamed the teacher.*<br>*HA5: we are only afraid that something worse can happen. For example, we give medication, suddenly the child's condition gets worse." (FGD 5_SS, teachers)*

For peer review only

No communicated individual asthma action plan

"I have a student with asthma, her mother said she has an inhaler, but she does not bring it to school. I've never seen it." (D_FGD 1, teacher)

Reliance on generic medical school plans

All participants stated that there was no specific asthma school plan in government schools (Table 3), so that staff followed the general circulars and their generic school plan for any illness or medical emergency. If a child was unwell, teachers would contact the parents to fetch them from school, and the parents decided to either treat the child themselves or to seek medical attention. In an emergency, teachers would take the child for medical care if it was clear a child needed urgent treatment, or their parent was taking too long to get to school.

"Usually what we do, first, is to call their parents. If their parents said, "okay, can bring them to the clinic". Then we will bring them to the nearest clinic. We will get a permission letter from the office." (A5_FGD 5_SS, teacher)

The teachers were unclear about their role in schoolchildren's health. The circulars stated that staff members were not allowed to give medication to students, but this was interpreted differently according to their personal views and the school management plan. Some teachers interpreted the circulars to only apply to non-prescribed medication and would act *in loco parentis* and give treatment to children only if they had clear instructions from their parents. A few teachers felt able to extend this to supervising prescribed medication such as an inhaler (albeit not being sure of the technique). Other teachers would not want to handle medication at all as they viewed medication use as the responsibility of parents and children. They feared their action might worsen the student's condition, e.g., giving the wrong dose, causing side

For peer review only

For peer review only - http://bmjopen.bmj.com/site/about/guidelines.xhtml

effects of medication, being blamed by parents as they were not trained to manage medical conditions.

> *"Sometimes parents called the teachers and said, "my child has an inhaler in his bag. Please can you supervise him"... Usually children will know how to use it. We just supervise when he is using it." (SA6_FGD 6_SS, teacher)*

No communicated individual asthma action plans

The absence of a specific asthma school plan and lack of clarity about the health role of teachers could delay treatment during asthma attacks. Teachers were unsure of what to do when dealing with children with asthma symptoms, some *'panicked'* while several teachers sought help from colleagues considered to have better skills in managing asthma. Both the teachers and the school health team observed that many children with asthma did not bring their reliever inhaler to school. Without a reliever inhaler, teachers tried different strategies when their students had symptoms, e.g., relaxation and breathing techniques while waiting for the parents to arrive.

> *"I was shocked when she was breathless; I am not sure how to help her. I patted her back and massaged her a bit, she was better, but after that, she coughed, and I panicked and quickly called her mother." (D1_FGD 1, teacher)*

Participants identified some poor asthma self-management practices on the part of parents. Teachers observed that parents rarely approached the school regarding their child's illness and few had shared their expectations and plans with teachers to support self-management of their child's illness at school (Table 3). Even when children had brought an inhaler to school, the teachers had not been asked to supervise its use.

> *"The child I told you earlier, his parents did not inform us... Only two parents in this school informed us about their child's illness." (HA5_IDI5, teacher)*

For peer review only

263 ***Suggested solutions***

264 Participants, particularly those with asthma experience and training, identified a need
265 for a school asthma programme to deliver asthma education and introduce an asthma
266 school plan (Table 4). On the other hand, participants without asthma experience or
267 training initially thought a school asthma programme was unnecessary, though some
268 changed their views during the focus group discussions as they became aware of the
269 risk of asthma attacks occurring at school.

> *"Asthma is not common in school. So, I don't think it (asthma programme) is necessary. But during this discussion, I think teachers should know how to help children with asthma if a child has an attack. It's better." (AN6_FGD 6, a teacher without asthma experience)*

270 To improve the self-management of children with asthma, healthcare professionals
271 and some teachers suggested the school programme could target the children, their
272 parents and teachers as primary school children were not capable of self-managing
273 asthma independently. The children could be taught to improve their self-management
274 skills e.g., how to avoid asthma triggers, monitor their symptoms, use a reliever
275 inhaler, and get help if having asthma symptoms/attacks. To support the self-
276 management of the children, their parents and teachers could receive similar asthma
277 education.

> *"The child themselves need to know, the severity of the symptoms,... the parents should know that the child has asthma... in the school, in case of any emergency, the teachers must know the child's asthma condition and how severe the asthma is" (SA4_IDI4, health policymaker)*

278 Participants believed good communication with parents would help school staff to
279 support a child's self-management. Prior agreement on emergency asthma school
280 plans could help boost teachers' confidence in dealing with an attack and protect them
281 from being blamed. A supporting letter from a healthcare professional regarding a
282 child's asthma condition and individual asthma action plan was suggested as another

For peer review only - http://bmjopen.bmj.com/site/about/guidelines.xhtml

way to provide the information to schools. A few healthcare professionals viewed schools as a platform to bridge communication with parents who did not bring their child for routine reviews.

> *"We need cooperation from the parent... parents should inform a teacher that 'my child has asthma and need this treatment'. So, the teacher will tell the other teachers and take care of the child... If parents provided a black and white instruction, that is even better." (T6_IDI 6, teacher)*

Table 4: Additional quotes illustrating themes for solutions, challenges and enablers.

**Suggested solutions**
"For improvement, we should ask parents to provide the asthma book together with their immunisation records. So, we can monitor their asthma appointment." (FA3_FGD 3, school health team)
**Implementation challenges**
"We have many programmes, like the health-promoting programme, vaccination programme, the teacher also requested us to finish as soon as possible because they had to go through their education programme." (K2_IDI 2, school health team)
**Implementation enablers**
"It has to be concise and simple. I think half an hour to an hour is enough. More than that, they (children) won't be able to concentrate." (AN6, F.G.D. 6, teacher)
"SA6/RA 6: Malay can understand.<br>AN6: Even if they cannot understand, a teacher will help to explain in Tamil. No problem." (F.G.D. 6, teachers)
"We have a parent and community organisation, and the school parent and teacher organisation who can help to promote the programme. Parents can know about the programme from them, and they can come." (T6_IDI 6, teacher)

For peer review only - http://bmjopen.bmj.com/site/about/guidelines.xhtml

Implementation challenges

Teachers and members of the school health team were quite negative about school asthma programmes because of the workload (Table 4). Teachers were already burdened with academic responsibilities and thought an intensive programme might affect children's learning and general education. The school health team had similar concerns and already felt overwhelmed by their workload, having to provide vaccination, screenings, and general health education in all the schools in the district.

> *"Cannot, we have a lot of programmes. There is no time to allocate in our schedule. A programme like this, can only be done infrequently." (SA6_FGD6, teacher)*

Teachers needed to recognise asthma symptoms/attacks and provide first aid asthma treatment, but the parents of children with asthma needed to take responsibility for self-management of their children. Parental participation in the programme was thus considered crucial but parents' attendance at school health activities was often very poor either because of work commitments or because they perceived that the health programme was not important for them.

> *"The lower education group… maybe they are not financially capable, care less about health, maybe work is a priority. So, if we invite parents to come, this group may not have time to attend." (A2_FGD 2, teacher)*

The target groups for the school asthma programme have different needs and capabilities. Primary schools covered a wide age range (7-12years) and the teachers' experience was that lessons needed to be customised to the child's educational level. Some teachers suggested the programme should be delivered in the native language of the children (including Tamil or Chinese as opposed to just the national language) for better understanding.

For peer review only

"HA7: English, Tamil can.
VA7: But if it is in Tamil, they will understand better, because it is their native language.
TA7: but some lower and upper primary can understand Malay." (FGD 7, teachers)

311 ***Implementation enablers***

312 Participants suggested simple, engaging, and tailored approaches catering for busy
313 schedules, and different individual needs and capabilities (Table 4). The delivery
314 ought to be fun and appealing to children and adults. They suggested quizzes,
315 demonstrations, and the use of digital media such as videos to assist sessions'
316 delivery.

"We can use visual aids, we teach first. After that, we ask them to demonstrate. Something like a simulation, if this happened, what will you do?" (NO7_IDI 7, teacher)

317 Healthcare professionals considered good partnerships with schools as an essential
318 enabler to promoting the uptake of school asthma programmes. The school health
319 team had a long-term relationship with the schools and suggested various approaches
320 to building the partnership including involvement of the school administrators at the
321 early stage of the programme development. A good partnership with schools also
322 helped identify and address implementation issues, for example, teachers from
323 Chinese/Tamil schools volunteered to translate the intervention to help children who
324 were not able to understand Malay.

"We have to hold a meeting with the headmaster and the teacher-in-charge of health... once the administrators are interested, they will suggest to you the teacher who will be in charge of the programme... So, you will work with the teacher to develop and run the programme." (RO_FGD 3, school health team)

325 All participants proposed that healthcare professionals should deliver the intervention
326 in schools, ideally as a multidisciplinary team including pharmacists and
327 paediatricians. To increase parents' participation, teachers suggested wider

For peer review only - http://bmjopen.bmj.com/site/about/guidelines.xhtml

community involvement such as parent-teacher associations, community organisations, and non-governmental organisations to help promote the intervention. Policymakers highlighted the need for introducing a national asthma school plan to improve asthma care of children during attacks.

> *"I think this matter will need a clear S.O.P. (standard operating procedure)… Although we have experience with our children, (but) for us to apply the same thing to someone's children, we are worried." (N08_IDI8, education policymaker)*

Discussion

Summary of findings

Asthma awareness and experience of managing asthma was limited among the school staff and, with no specific asthma school plan to provide guidance, treatment during asthma symptoms/emergencies could be inappropriate or delayed. School health circulars were not clear about the teachers' health role in schools. To improve this, participants suggested a school asthma programme that focused on introducing an asthma school plan and improving awareness about asthma amongst children, their parents and teachers. Communication between healthcare professionals, parents and the school also needed improvement. The participants anticipated heavy workload, poor parental participation and diverse individual capabilities and needs as challenges to implementing school asthma programmes. Tailored approaches, good partnership with schools and multi-organisation involvement were discussed as enablers to develop and deliver the programme. We will use these findings to inform the development of a school-based asthma programme in Malaysia.

Study findings in comparison with other studies

Our interviewees suggested developing a school asthma programme involving parents and teachers, that focussed on asthma awareness, and an asthma school plan for use

For peer review only

in attacks. Similar community-wide programmes have been implemented in other contexts.[32 33] Education for staff and children could correct common misperceptions about asthma and asthma medication and involvement of school health teams could improve the accessibility of healthcare for children with asthma.[19 34-36] School asthma programmes offer the opportunity to focus self-management education on children and involve children's social network to support their self-management skills and practices.[37 38]

The lack of awareness among school staff about which students had asthma, combined with poor understanding of symptoms and first aid treatment leads to hesitancy and delay in taking action risking deterioration or even death.[39 40] Delineating the health roles of teachers in an asthma school plan and providing adequate training for school staff could improve their confidence and prevent treatment delay.[16 40] However, such strategies rely on parents providing asthma reliever medication; without this, school staff will have to call parents or get immediate medical help.[39 41] School asthma guidelines in Australia, Canada, United Kingdom and United States encourage inhalers to be labelled and kept with the child or the class teacher in primary schools without a school nurse.[7 42-44] These guidelines also recommend that schools should have an emergency asthma kit to include a reliever inhaler, spacer and agreed information on when and how to use it.[7-9 43] Establishing immediate access to an emergency reliever inhaler in schools required high-level policy changes and detailed guidance for using first aid asthma treatment.[43 45] Therefore, a clear guideline to inform stakeholders e.g. policymakers, school leaders, parents and the community will aid the development and implementation of school asthma programmes.

For peer review only

Highlighting the importance of the programmes to stakeholders particularly policymakers could ensure successful implementation of school asthma programmes. Policy supporting improvement in school asthma management is a powerful tool in prioritising and providing resources to assist implementation. A good partnership with schools throughout the development of asthma school plans and programmes is crucial to identify challenges and enablers for successful implementation.[46 47] The presence of school plans does not guarantee teachers are equipped with the skills to manage asthma symptoms/attacks.[48] Teachers need to be aware of the school plan, and receive education and training in use of reliever treatment to equip them to handle asthma events in school.[48 49] Tailoring designs according to the needs and capabilities of individuals and schools could improve the uptake and mitigate the challenges of the programmes.[19 50] Healthcare providers play a crucial role in the partnership. They need to provide personalised self-management education to a child including assessing if the child can self-carry and self-administer their medication. They can also provide a personalised asthma action plan to be shared with the school.[5 45] Involving the wider community, e.g. parent and teacher association, health care providers and social services may support delivery and sustainability of school asthma programmes.[19 51 52]

Study strengths and limitations

This is one of a few studies on school asthma programmes conducted in low-middle income countries. Flexible time, venue and data collection methods facilitated recruitment of a study population with various backgrounds which contributed to the diverse views of participants. Nevertheless, the findings may not be wholly applicable in other settings, and an intervention based on our findings will need adaptation for

For peer review only

401 other contexts. Memos, field notes and multidisciplinary team discussions aided
402 analysis and informed interpretation.
403
404 Some loss of meaning may have occurred during the translation of Malay quotes;
405 however, we analysed the data in the source language and checked the semantic and
406 context equivalence for translated quotes. Thematic analysis may reduce the
407 interpretive details of each participant' as the coding process focuses on the content,
408 and less on the processes and phenomena within individuals. However, we used a
409 pragmatic, inductive approach with constant comparisons of data and discussions to
410 inform our understanding of the views expressed. We were aware of the risk of
411 researcher bias in qualitative research, and actively discussed reflexivity in the regular
412 multidisciplinary team discussions which aided analysis and informed interpretation.
413 Participants with less experience on the topic may have contributed less to the focus
414 groups though they were provided with opportunities to provide their views.
415

416 Conclusions

417 School asthma programmes could improve the care and support for children with
418 asthma. Aligned with the WHO Global School Health Initiative recommendations[1], the
419 participants in our study highlighted the importance of tailored approaches, a good
420 partnership with schools and engaging the wider community at an early stage of
421 developing a school asthma programme. There is a need for a clear asthma guideline
422 on school-based programmes to inform policymakers, school leaders, and the
423 community in Malaysia.

424 **Data Availability**
425 Due to ethical concerns, supporting data cannot be made openly available and are stored under restricted
426 access in the University of Edinburgh DataVault. Further information about the data and conditions for
427 access are available via the University of Edinburgh Research Explorer

For peer review only - http://bmjopen.bmj.com/site/about/guidelines.xhtml

[https://doi.org/10.7488/191f40bd-ace9-4c8a-a36c-43b796303166]. Additional non-disclosive details relating to other aspects of the data are openly available from the University of Edinburgh DataShare repository at [https://doi.org/10.7488/ds/3004].

Acknowledgements

We would like to acknowledge all the participants who voluntarily participated in this research and the local authorities for their support during the study. The RESPIRE collaboration comprises the UK Grant holders, Partners and research teams as listed on the RESPIRE website (www.ed.ac.uk/usher/respire), including Nik Sherina Hanafi and Norita Binti Hussein.

Funding

This research was commissioned by the UK National Institute for Health Research (NIHR) Global Health Research Unit on Respiratory Health (RESPIRE), using UK Aid from the UK Government. The views expressed in this publication are those of the author(s) and not necessarily those of the NIHR or the UK Department of Health and Social Care.

Competing Interest

None declared.

Author contributions

All authors (SNR, EMK, SML, SC and HP) contributed to the study conception. SNR performed the data collection, checking and coded the transcripts. All authors (SNR, EMK, SML, SC and HP) contributed to data analysis and interpretation of data. SNR drafted the manuscript and all authors (SNR, EMK, SML, SC and HP) provided critical revisions and editing of the manuscript.

References

1. World Health Organisation. Making every school a health-promoting school. Geneva, Switzerland, 2021.
2. World Health Organization. Global School Health Initiatives: achieving health and education outcomes. Report of a meeting, Bangkok,Thailand, 23–25 November 2015. . Geneva, Switzerland: World Health Organisation, 2017.
3. Levinson J, Kohl K, Baltag V, et al. Investigating the effectiveness of school health services delivered by a health provider: A systematic review of systematic reviews. *PLoS One* 2019;14(6):e0212603. doi: 10.1371/journal.pone.0212603 [published Online First: 2019/06/13]
4. Global Asthma Network. The Global Asthma Report. Auckland, New Zealand, 2018.
5. Global Initiative for Asthma. Global Strategy for Asthma Prevention, 2021.
6. Scottish Intercollegiate Guidelines Network/British Thoracic Society. SIGN 158 British guideline on the management of asthma. London: Scottish Intercollegiate Guidelines Network/British Thoracic Society, 2019.
7. Asthma Australia. Asthma Guidelines for Queenlands schools Australia: Asthma Australia, 2019.
8. Healthy London Partnership. London schools' guide for the care of children and young people with asthma: Pre-school, primary and secondary school years: London, 2016.
9. U.S. Department of Health and Human Services. Managing Asthma: A guide for schools: National Institutes of Health, 2014.
10. Piot P, Caldwell A, Lamptey P, et al. Addressing the growing burden of non-communicable disease by leveraging lessons from infectious disease management. *J Glob Health* 2016;6(1):010304-04. doi: 10.7189/jogh.06.010304

For peer review only

11. World Health Organisation. WHO guideline on school health services. . Geneva, 2021.
12. Harris K, Kneale D, Lasserson TJ, et al. School-based self-management interventions for asthma in children and adolescents: a mixed methods systematic review. *Cochrane Database Syst Rev,* 2019;1(1):CD011651. doi: 10.1002/14651858.CD011651.pub2 [published Online First: 2019/01/29]
13. Walter H, Sadeque-Iqbal F, Ulysse R, et al. Effectiveness of school-based family asthma educational programs in quality of life and asthma exacerbations in asthmatic children aged five to 18: a systematic review. *JBI Database System Rev Implement Rep* 2016;14(11):113-38. doi: 10.11124/JBISRIR-2016-003181 [published Online First: 2016/12/13]
14. Isik E, Fredland NM, Freysteinson WM. School and Community-based Nurse-led Asthma Interventions for School-aged Children and Their Parents: A Systematic Literature Review. *J Pediatr Nurs* 2019;44:107-14. doi: 10.1016/j.pedn.2018.11.007 [published Online First: 2019/01/27]
15. Al Aloola NA, Naik-Panvelkar P, Nissen L, et al. Asthma interventions in primary schools--a review. *J Asthma* 2014;51(8):779-98. doi: 10.3109/02770903.2014.914534 [published Online First: 2014/04/16]
16. Kew KM, Carr R, Donovan T, et al. Asthma education for school staff. *Cochrane Database Syst Rev,* 2017;4:CD012255. doi: 10.1002/14651858.CD012255.pub2 [published Online First: 2017/04/13]
17. Craig P, Dieppe P, Macintyre S, et al. Developing and evaluating complex interventions: the new Medical Research Council guidance. *Int J Nurs Stud* 2013;50(5):587-92. doi: 10.1016/j.ijnurstu.2012.09.010 [published Online First: 2012/11/20]
18. Wight D, Wimbush E, Jepson R, et al. Six steps in quality intervention development (6SQuID). *J Epidemiol Community Health* 2016;70(5):520-5. doi: 10.1136/jech-2015-205952 [published Online First: 2015/11/18]
19. Cicutto L, Gleason M, Haas-Howard C, et al. Building Bridges for Asthma Care Program: A School-Centered Program Connecting Schools, Families, and Community Health-Care Providers. *J Sch Nurs,* 2020;36(3):168-80. doi: 10.1177/1059840518805824 [published Online First: 2018/10/20]
20. Ahad A, Khoo EM. Asthma Control and Care Among Malaysian Primary School Children: A Cross-Sectional Study. *Asia Pac J Public Health* 2017;29(5):422-29. doi: 10.1177/1010539517717766 [published Online First: 2017/07/19]
21. Asher MI, Montefort S, Bjorksten B, et al. Worldwide time trends in the prevalence of symptoms of asthma, allergic rhinoconjunctivitis, and eczema in childhood: ISAAC Phases One and Three repeat multicountry cross-sectional surveys. *Lancet (London, England)* 2006;368(9537):733-43. doi: 10.1016/S0140-6736(06)69283-0
22. Wong GW, Kwon N, Hong JG, et al. Pediatric asthma control in Asia: phase 2 of the Asthma Insights and Reality in Asia-Pacific (AIRIAP 2) survey. *Allergy* 2013;68(4):524-30. doi: 10.1111/all.12117 [published Online First: 2013/02/19]
23. Kementerian Kesihatan Malaysia. Garis Panduan Perkhidmatan Kesihatan Sekolah. Malaysia, 2013.
24. Ramdzan SN, Pinnock H, Liew SM, et al. Perceptions of complementary/alternative medicine use and influence on evidence-based asthma medicine adherence in Malaysian children. *NPJ Prim Care Respir Med* 2019;29(1):5. doi: 10.1038/s41533-019-0118-x [published Online First: 2019/02/26]

For peer review only - http://bmjopen.bmj.com/site/about/guidelines.xhtml

25. Ramdzan SN, Khoo EM, Liew SM, et al. How young children learn independent asthma self-management: a qualitative study in Malaysia. *Arch Dis Child* 2020;105(9):819-24. doi: 10.1136/archdischild-2019-318127 [published Online First: 2020/07/06]

26. Malaysia Education Statistics. Quick Facts 2018. Malaysia: Educational Planning and Research Division, Ministry of Education Malaysia, 2018.

27. Braun V, Clarke V. Succesful Qualitative Research: a practical guide for beginners. London: SAGE Publication Ltd 2013.

28. Silverman D. Doing Qualitative Research. 5th edition ed. London, United Kingdom: SAGE Publication Limited 2018.

29. Farre A, Wood V, McDonagh JE, et al. Health professionals' and managers' definitions of developmentally appropriate healthcare for young people: conceptual dimensions and embedded controversies. *Arch Dis Child* 2016;101(7):628-33. doi: 10.1136/archdischild-2015-309473 [published Online First: 2016/03/06]

30. Borrell-Carrio F, Suchman AL, Epstein RM. The biopsychosocial model 25 years later: principles, practice, and scientific inquiry. *Ann Fam Med* 2004;2(6):576-82. doi: 10.1370/afm.245 [published Online First: 2004/12/04]

31. Grbich C. Qualitative Data Analysis: An introduction. Second ed. United Kingdom: SAGE Publication 2013.

32. Lenney W, Bush A, Fitzgerald DA, et al. Improving the global diagnosis and management of asthma in children. *Thorax* 2018;73(7):662-69. doi: 10.1136/thoraxjnl-2018-211626

33. Szefler SJ, Fitzgerald DA, Adachi Y, et al. A worldwide charter for all children with asthma. *Pediatr Pulmonol* 2020;55(5):1282-92. doi: 10.1002/ppul.24713 [published Online First: 2020/03/07]

34. Suwannakeeree P, Deerojanawong J, Prapphal N. School-Based Educational Interventions Can Significantly Improve Health Outcomes in Children with Asthma. *J Med Assoc Thai* 2016;99(2):166-74. [published Online First: 2016/06/03]

35. Clark NM, Gong M, Kaciroti N, et al. A trial of asthma self-management in Beijing schools. *Chronic illness* 2005;1(1):31-8. doi: 10.1177/17423953050010010101 [published Online First: 2006/12/02]

36. Carvalho Coelho AC, Barretto Cardoso LS, de Souza-Machado C, et al. The Impacts of Educational Asthma Interventions in Schools: A Systematic Review of the Literature. *Can Respir J,* 2016;2016:8476206. doi: 10.1155/2016/8476206 [published Online First: 2016/09/23]

37. Szefler SJ, Cloutier MM, Villarreal M, et al. Building Bridges for Asthma Care: Reducing school absence for inner-city children with health disparities. *J Allergy Clin Immunol* 2019;143(2):746-54 e2. doi: 10.1016/j.jaci.2018.05.041 [published Online First: 2018/07/29]

38. Lemanske RF, Jr., Kakumanu S, Shanovich K, et al. Creation and implementation of SAMPRO: A school-based asthma management program. *J Allergy Clin Immunol* 2016;138(3):711-23. doi: 10.1016/j.jaci.2016.06.015 [published Online First: 2016/09/07]

39. Greiling AK, Boss LP, Wheeler LS. A preliminary investigation of asthma mortality in schools. *J Sch Health,* 2005;75(8):286-90. doi: 10.1111/j.1746-1561.2005.00039.x [published Online First: 2005/09/24]

40. Murphy KR, Hopp RJ, Kittelson EB, et al. Life-threatening asthma and anaphylaxis in schools: a treatment model for school-based programs. *Ann Allergy Asthma*

For peer review only - http://bmjopen.bmj.com/site/about/guidelines.xhtml

Immunol 2006;96(3):398-405. doi: 10.1016/S1081-1206(10)60906-4 [published Online First: 2006/04/07]

41. Gerald JK, Stroupe N, McClure LA, et al. Availability of Asthma Quick Relief Medication in Five Alabama School Systems. *Pediatr Allergy Immunol Pulmonol* 2012;25(1):11-16. doi: 10.1089/ped.2011.0118 [published Online First: 2012/03/29]
42. Council on School H. Policy statement--guidance for the administration of medication in school. *Pediatrics* 2009;124(4):1244-51. doi: 10.1542/peds.2009-1953 [published Online First: 2009/09/30]
43. Department of Health. Guidance on the use of emergency salbutamol inhalers in schools. Storage and care of the inhaler. United Kingdom, 2015.
44. OPHEA Health schools and Healthy Communities. Creating Asthma friendly schools. Canada, 2015.
45. Toups MM, Press VG, Volerman A. National Analysis of State Health Policies on Students' Right to Self-Carry and Self-Administer Asthma Inhalers at School. *J Sch Health,* 2018;88(10):776-84. doi: 10.1111/josh.12681 [published Online First: 2018/09/12]
46. Cicutto L, Gleason M, Szefler SJ. Establishing school-centered asthma programs. *J Allergy Clin Immunol* 2014;134(6):1223-30. doi: 10.1016/j.jaci.2014.10.004 [published Online First: 2014/12/09]
47. Kakumanu S, Lemanske RF, Jr. Asthma in Schools: How School-Based Partnerships Improve Pediatric Asthma Care. *Immunol Allergy Clin North Am* 2019;39(2):271-81. doi: 10.1016/j.iac.2018.12.011 [published Online First: 2019/04/08]
48. Jaramillo Y, Reznik M. Do United States' teachers know and adhere to the national guidelines on asthma management in the classroom? A systematic review. *The Scientific World Journal* 2015;2015:624828. doi: 10.1155/2015/624828 [published Online First: 2015/03/03]
49. Cain A, Reznik M. Asthma management in New York City schools: A classroom teacher perspective. *J Asthma* 2016;53(7):744-50. doi: 10.3109/02770903.2015.1135946 [published Online First: 2016/04/01]
50. Brigden A, Parslow RM, Linney C, et al. How are behavioural interventions delivered to children (5-11 years old): a systematic mapping review. *BMJ Paediatr Open* 2019;3(1):e000543. doi: 10.1136/bmjpo-2019-000543 [published Online First: 2020/01/08]
51. McCabe EM, Kaskoun JR, Murphy EL, et al. An Evidence-Based Framework for Implementation of a School-Community Partnership. *NASN Sch Nurse* 2021;36(3):164-69. doi: 10.1177/1942602X20964765 [published Online First: 2020/10/16]
52. Clark NM, Lachance L, Doctor LJ, et al. Policy and system change and community coalitions: outcomes from allies against asthma. *American journal of public health* 2010;100(5):904-12. doi: 10.2105/AJPH.2009.180869 [published Online First: 2010/03/18]

Figure legend

Figure 1: Themes and subthemes of participants' views of asthma and school-based asthma intervention.

For peer review only - http://bmjopen.bmj.com/site/about/guidelines.xhtml

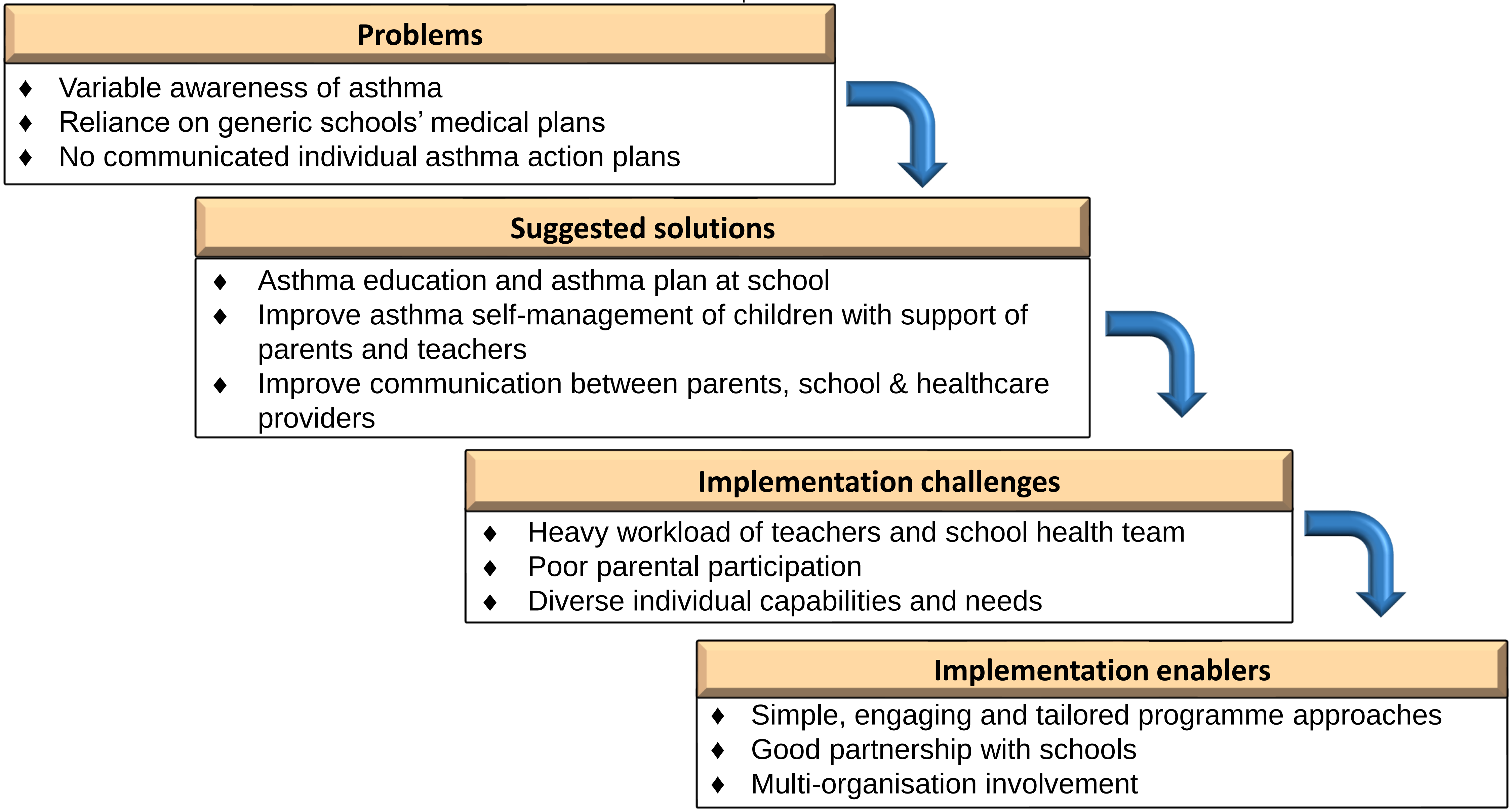

Figure 1 : Themes and subthemes of participants' views of asthma and school-based asthma intervention.

For peer review only - http://bmjopen.bmj.com/site/about/guidelines.xhtml

Borang soal selidik latar belakang
Socio-demographic questionnaire

Latar belakang
Background

Umur*(Age)* : ___________

Jantina*(Gender)* : ___________

Bangsa*(Ethnicity)* : ___________

Status perkahwinan *(Marital status)*:

Bujang*(Single)*/Bercerai *(Divorced)*/Berkahwin*(Married)*

Tahap pendidikan tertinggi *(Highest educational level achieved):*

Rendah*(Primary)*/Menengah *(Secondary)*/Pengajian Tinggi *(Tertiary)*

Pekerjaan *(Occupation)* : _____________________

Pengalaman berkerja (work experience) : ……….. /tahun(year)

Anggaran pendapatan isi rumah

(Estimated family income): RM ________ /bulan(month)

Informasi mengenai asma
Information about asthma

Adakah anda menghidap asma *(Do you have asthma)?*

Ya *(yes)*/ Tidak *(No)*

Adakah ahli keluarga menghidap asma (Anyone in your family has asthma)?

Ya *(yes)*/ Tidak *(No)*

Jika Ya, sila nyatakan (If yes, (please specify):

____

For peer review only

Childhood Asthma
Participant Interview Topic Guide (school staff)

Preamble:
- **Ice-breaking and explain that there is no right or wrong answer**
- **Explain need to get consent for the interview and audio-recording. Obtain consents.**
- **Explain that the participant does not have to answer if he or she does not wish to do so**

Question	Prompts
**Asthma experience**	
Tell us your experience in dealing with children with asthma?	Your child? Your student?
What do you know about asthma?	Experience? Reading? Told by someone?
How do you know if a child's asthma is good or badly controlled?	Able to play? Easy breathing? No cough? How do you know about this?
How does a child's asthma disturb/not disturb he/her? How? How does a child's asthma disturbs/not disturb you? How?	Activities affected e.g. attending school? *Pendidikan Jasmani dan kesihatan* (PJK)/sports?
In your opinion, how does having asthma makes a child feel?	Emotionally-friends isolation/bully? Shy to use medication?
**Asthma treatment**	
Do you know of any asthma treatment?	Pills? MDI? Spacer? Aerochamber?
How about complementary and alternative medicine for asthma?	
**Asthma management at school**	
What do you do when a student has an asthma attack?	Inhaler? Go to the hospital/clinic? Call parents?
How do you recognise a severe asthma attack? What would you do if this happened?	Breathlessness? Unable to speak?
Is there anything else you would do?	Concern on the administration of medication eg MDI to student(s)? Rules or legislation?
Is there any rules/limitation for students with asthma at school?	Sports? Physical activity? Extracurricular activities?
Is there any asthma education or campaign at school?	How is it delivered? By whom? To who?
What do you think about asthma education at school? Is it necessary?	Feasible? How best to deliver? Parents and school staffs involvement? How often?
What do you think if school staffs are allowed to deliver/supervise the use of asthma medication e.g inhaler at school?	Good? Not good? Concerns?

For peer review only

Suggestions/ideas for intervention?<br><br>Any suggestion on how to improve asthma management at school?	Others? Timing – during or after school?
**Health belief about asthma**<br><br>Why do you think a child has asthma?<br><br>What do you think a child can do to prevent asthma?	 <br>Inherited? Infection? Environment?<br><br>Avoidance of food? Medication? Sports?
**Source of information**<br><br>Where and from whom did you get information about asthma?<br><br>Anything else that you want to share about childhood asthma?	 <br>Clinic? Family? Friends? Internet? How good is the information?

For peer review only

Childhood Asthma (CuT-AsthMa)
Participant Interview Topic Guide (School Health Team)

Preamble:

- **Ice-breaking and explain that there is no right or wrong answer**
- **Explain the need to get consent for the interview and audio-recording. Obtain consents.**
- **Explain that the participant does not have to answer if he or she does not wish to do so**

Question	Prompts
**Asthma experience**	
Tell us your experience about dealing with children with asthma?	A student? A patient? Your own child?
How do you know that they have asthma?	Symptoms e.g cough, wheezing? Informed by parents?
How do you know if the child's asthma is good or badly controlled?	Able to play? Easy breathing? No cough? Where do you know about this?
How does a student's asthma disturb/not disturb him/her?	Physically? Activities affected at school e.g PJK, absent from school?
How does a student's asthma disturbs/not disturb you? How?	
In your opinion, how does having asthma makes a student feel?	Emotionally-friends isolation/bully? Shy to use medication?
**Asthma treatment**	
Do you know of any asthma treatment?	Pills? MDI? Spacer? Aerochamber?
How about complementary and alternative medication for asthma?	
**Asthma management at school**	
What do you do when a student has asthma attack?	Inhaler? Go to hospital/clinic? Asthma action plan? How do you know this?
How do you recognise a severe asthma attack? What would you do if this happened?	Breathlessness? Unable to speak?
Is there anything else you would do?	
	Sports? Physical activity? Extracurricular activities?

For peer review only

Is there any rules/limitation for students with asthma at school?<br><br>Is there any asthma education or campaign at school?<br><br>What do you think of asthma education at school? Is it necessary? Is it feasible?<br><br>What is your opinion on teachers delivering/supervising the use of asthma medication e.g inhaler at school?<br><br>Any suggestion on how to improve asthma management at school?	How is it delivered? By whom? To who?<br><br>Feasible? How best to deliver? Parents and teachers involvement? How often?<br><br>Good? Not good? Any worries?
**Health belief about asthma**<br><br>Why do you think a child has asthma?<br><br>What do you think a child can do to prevent asthma?	Inherited? Infection? Environment?<br><br>Avoidance of food? Medication? Sports?
**Source of information**<br><br>Where and from whom did you get information about asthma?<br><br>Anything else that you want to share about asthma among children?	Clinic? Family? Friends? Internet? How good is the information?

For peer review only

Childhood Asthma (CuT-AsthMa)
Participant focus group topic guide (Healthcare professionals/policymakers)
(Interviews, which will be offered as an option to suit the convenience/preference of the participant, will follow the same topic guide)

Preamble:

- **Ice-breaking and explain that there is no right or wrong answer**
- **Explain the need to get consent for the focus group/interview and audio-recording. Obtain consents.**
- **Explain that the participant does not have to answer if he or she does not wish to do so**

Question	Prompts
**Asthma experience**	
Tell us about your experience in dealing with children with asthma?	A student? A patient? Your child?
How do you know that they have asthma?	Symptoms e.g cough, wheezing? Informed by parents?
How do you know if the child's asthma is good or badly controlled?	Able to play? Easy breathing? No cough? Where do you know about this?
How does a student's asthma disturb/not disturb him/her? How does a student's asthma disturbs/not disturb you? How?	Physically? Activities affected at school e.g PJK, absent from school?
In your opinion, how does having asthma makes a student feel?	Emotionally-friends isolation/bully? Shy to use medication?
**Asthma treatment**	
Do you know of any asthma treatment?	Pills? MDI? Spacer? Aerochamber?
How about complementary and alternative medicine for asthma?	
**Asthma management at school**	
What do you do when a student has an asthma attack?	Inhaler? Go to the hospital/clinic? Asthma action plan? How do you know this?
How do you recognise a severe asthma attack? What would you do if this happened?	Breathlessness? Unable to speak?
Is there anything else you would do?	Sports? Physical activity? Extracurricular activities?
Is there any rules/limitation for students with asthma at school?	How is it delivered? By whom? To who?
Is there any asthma education or campaign at school?	
What do you think of asthma education at school? Is it necessary? Is it feasible?	Feasible? How best to deliver? Parents and teachers involvement? How often?

For peer review only - http://bmjopen.bmj.com/site/about/guidelines.xhtml

What is your opinion on teachers delivering/supervising the use of asthma medication e.g inhaler at school?<br><br>Any suggestion on how to improve asthma management at school?	Good? Not good? Any worries?
**Health belief about asthma**<br><br>Why do you think a child has asthma?<br><br>What do you think a child can do to prevent asthma?	 <br>Inherited? Infection? Environment?<br><br>Avoidance of food? Medication? Sports?
**Source of information**<br><br>Where and from whom did you get information about asthma?<br><br>Anything else that you want to share about asthma among children?	 <br>Clinic? Family? Friends? Internet? How good is the information?

For peer review only

For peer review only - http://bmjopen.bmj.com/site/about/guidelines.xhtml

Standards for Reporting Qualitative Research (SRQR)*

http://www.equator-network.org/reporting-guidelines/srqr/

Title and abstract

	Page/line no(s).
**Title** - Concise description of the nature and topic of the study Identifying the study as qualitative or indicating the approach (e.g., ethnography, grounded theory) or data collection methods (e.g., interview, focus group) is recommended	1/1-3
**Abstract** - Summary of key elements of the study using the abstract format of the intended publication; typically includes background, purpose, methods, results, and conclusions	2-3/27-54

Introduction

**Problem formulation** - Description and significance of the problem/phenomenon studied; review of relevant theory and empirical work; problem statement	4-5/70-105
**Purpose or research question** - Purpose of the study and specific objectives or questions	5/101-105

Methods

**Qualitative approach and research paradigm** - Qualitative approach (e.g., ethnography, grounded theory, case study, phenomenology, narrative research) and guiding theory if appropriate; identifying the research paradigm (e.g., postpositivist, constructivist/ interpretivist) is also recommended; rationale**	7-6/154-158
**Researcher characteristics and reflexivity** - Researchers' characteristics that may influence the research, including personal attributes, qualifications/experience, relationship with participants, assumptions, and/or presuppositions; potential or actual interaction between researchers' characteristics and the research questions, approach, methods, results, and/or transferability	8/163-171
**Context** - Setting/site and salient contextual factors; rationale**	6/116-128
**Sampling strategy** - How and why research participants, documents, or events were selected; criteria for deciding when no further sampling was necessary (e.g., sampling saturation); rationale**	1/36-37 and 7/141-143
**Ethical issues pertaining to human subjects** - Documentation of approval by an appropriate ethics review board and participant consent, or explanation for lack thereof; other confidentiality and data security issues	6/108-114
**Data collection methods** - Types of data collected; details of data collection procedures including (as appropriate) start and stop dates of data collection and analysis, iterative process, triangulation of sources/methods, and modification of procedures in response to evolving study findings; rationale**	7/133-144

For peer review only - http://bmjopen.bmj.com/site/about/guidelines.xhtml

**Data collection instruments and technologies** - Description of instruments (e.g., interview guides, questionnaires) and devices (e.g., audio recorders) used for data collection; if/how the instrument(s) changed over the course of the study	7/143-144
**Units of study** - Number and relevant characteristics of participants, documents, or events included in the study; level of participation (could be reported in results)	8-9/180-188 (Table 1)
**Data processing** - Methods for processing data prior to and during analysis, including transcription, data entry, data management and security, verification of data integrity, data coding, and anonymization/de-identification of excerpts	8/-143-151
**Data analysis** - Process by which inferences, themes, etc., were identified and developed, including the researchers involved in data analysis; usually references a specific paradigm or approach; rationale**	7-8/154-171
**Techniques to enhance trustworthiness** - Techniques to enhance trustworthiness and credibility of data analysis (e.g., member checking, audit trail, triangulation); rationale**	7-8/156-16

Results/findings

**Synthesis and interpretation** - Main findings (e.g., interpretations, inferences, and themes); might include development of a theory or model, or integration with prior research or theory	8-15/180-308, Figure 1
**Links to empirical data** - Evidence (e.g., quotes, field notes, text excerpts, photographs) to substantiate analytic findings	8-15/quotes and Table 3-4

Discussion

**Integration with prior work, implications, transferability, and contribution(s) to the field -** Short summary of main findings; explanation of how findings and conclusions connect to, support, elaborate on, or challenge conclusions of earlier scholarship; discussion of scope of application/generalizability; identification of unique contribution(s) to scholarship in a discipline or field	15-19/311-392
**Limitations** - Trustworthiness and limitations of findings	18-19/373-392

Other

**Conflicts of interest** - Potential sources of influence or perceived influence on study conduct and conclusions; how these were managed	19/422
**Funding** - Sources of funding and other support; role of funders in data collection, interpretation, and reporting	19/416-419

*The authors created the SRQR by searching the literature to identify guidelines, reporting standards, and critical appraisal criteria for qualitative research; reviewing the reference lists of retrieved sources; and contacting experts to gain feedback. The SRQR aims to improve the transparency of all aspects of qualitative research by providing clear standards for reporting qualitative research.

For peer review only

For peer review only - http://bmjopen.bmj.com/site/about/guidelines.xhtml

**The rationale should briefly discuss the justification for choosing that theory, approach, method, or technique rather than other options available, the assumptions and limitations implicit in those choices, and how those choices influence study conclusions and transferability. As appropriate, the rationale for several items might be discussed together.

Reference:

O'Brien BC, Harris IB, Beckman TJ, Reed DA, Cook DA. **Standards for reporting qualitative research: a synthesis of recommendations.** *Academic Medicine*, Vol. 89, No. 9 / Sept 2014 DOI: 10.1097/ACM.0000000000000388

For peer review only

For peer review only - http://bmjopen.bmj.com/site/about/guidelines.xhtml

Stakeholders' views of asthma and school-based asthma programme for primary school children: A qualitative study in Malaysia

Siti Nurkamilla Ramdzan, MMed[1,2], Ee Ming Khoo, MD[1], Su May Liew, DPhil[1], Steve Cunningham, PhD[2], Hilary Pinnock, MD*[2] on behalf of RESPIRE collaborators

[1]*Department of Primary Care Medicine, Faculty of Medicine, University of Malaya, Malaysia*

[2]*NIHR Global Health Research Unit on Respiratory Health (RESPIRE), Usher Institute, University of Edinburgh, United Kingdom*

Address for correspondence
*Professor Hilary Pinnock
NIHR Global Health Research Unit on Respiratory Health (RESPIRE),
Usher Institute,
The University of Edinburgh.
Doorway 3, Medical School, Teviot Place,
Edinburgh EH8 9AG, UK.
Tel: 0131 650 9474
Email: Hilary.Pinnock@ed.ac.uk

Main text word count: 3876~~604~~ (excluding abstract, main headings, tables, references, and additional information, e.g. acknowledgement, funding, competing interest) ~~-~~Maximum: 4000 words

For peer review only

Abstract

Objective: The WHO Global School Health Initiative aimed to improve child and community health through health promotion programmes in schools, though most focus~~sed~~ on preventing communicable disease. Despite WHO recommendations~~The Asthma~~ , no asthma programme is included in the Malaysian national school health service guideline. We ~~therefore aimed~~ ~~is the commonest non-communicable disease among children and has the possibility of requiring urgent treatment during school hours, an important exemplar for school-based programmes.~~ t~~The objective~~ of this study ~~was~~ to explore the views of school staff, healthcare professionals and policymakers, about asthma and school asthma programmes for primary school children.

Design: A qualitative study using purposive sampling of participants to obtain diverse views. We conducted focus groups and individual interviews using piloted semi-structured topic guides. The focus groups and interviews were audio-recorded, transcribed verbatim and analysed using inductive thematic analysis. We completed data collection once data saturation was reached.

Setting: Stakeholders in education and health sectors in Malaysia.

Participants: Fifty-two participants (40 school staff, 9 healthcare professionals and 3 policymakers) contributed to nine focus groups and eleven individual interviews.

Results: School staff had limited awareness of asthma and first-aid asthma treatments. There was no guidance on asthma management in government schools, and teachers were unclear about their role in school children's health. These uncertainties led to delays in the treatment of asthma symptoms/attacks, and suggestions that an asthma education programme and a school plan would improve asthma care. Perceived challenges in conducting school health programmes included a busy school schedule

For peer review only

and poor parental participation. A tailored asthma programme ~~with~~ in partnerships ~~from~~ with schools ~~and multi-organisation~~ could facilitate the programme's adoption and ~~/~~implementation.

Conclusions: Identifying and addressing issues and challenges specific to the school and wider community c~~w~~ould facilitate the delivery of a school asthma programme in line with the WHO School Health Initiative. Clarity over national policy on the role~~/~~s and responsibilities of school staff could support implementation and guide appropriate and prompt response to asthma emergencies in schools.

Word count: 286~~299~~ (Maximum: 300)

Keywords: school-based, asthma, complex intervention, low-middle income countries and health behaviour

For peer review only

For peer review only - http://bmjopen.bmj.com/site/about/guidelines.xhtml

Strengths and limitations of this study

- One of a few studies on school asthma programmes conducted in low-middle income countries.
- Various backgrounds contributed to the diverse views of participants.
- Some loss of meaning may have occurred during the translation of Malay quotes to English but the analysis was conducted in the source language.
- Participants with less experience on the topic may have contributed less to the focus groups though they were provided with opportunities to provide their views.

Introduction

The Global School Health Initiative launched by the World Health Organiz~~s~~ation (WHO) in 1995 aimed to improve child, adolescent and community health through health promotion programmes in schools.[1] Globally, the compulsory school years offer an 'easy entry point' for embedding healthy behaviours at a young age and, by extension, influencing families.[2] Health promotion programmes (e.g. de-worming, vaccination, water, sanitation, hygiene) have been successfully implemented in low-middle income countries (LMICs) through this initative.[2] Non-communicable diseases (NCDs) are an increasing challenge in all economies, though successful NCD programmes have typically been focussed in high-income countries.[2 3]

Asthma is the commonest NCD in children ~~among children and~~ causing~~es~~ significant morbidity and mortality globally.[4] Asthma symptoms and attacks can occur unexpectedly, and schools need plans for responding appropriately and promptly to asthma symptoms.[5 6] Young children can spend up to half their waking hours in

For peer review only

schools, and school staff could support the development of asthma self-management skills in the students.[7-9] A school asthma programme offers an exemplar for school health NCD~~s~~ programmes and the WHO recommended asthma as an essential school health service in all countries.[3 10 11]

School-based asthma self-management interventions have been shown to improve asthma control, reduce school absenteeism and asthma exacerbations[12-15], though few were conducted in LMICs.[12 15 16] These programmes are complex interventions that need to be tailored to the target population[17 18], and adapted to the context.[19] In a review of the first two decades of school health initiatives, the WHO highlighted the importance of policy and organisation-level collaboration and engagement with parents, students and teachers.[2]

In Malaysia, an estimated 9% of children have asthma [20 21], at least half of whom are poorly controlled.[20 22] Despite this morbidity, the national school health service guideline does not include an asthma programme [23] , and a qualitative study conducted among primary school children with asthma and their parents revealed a perception of poor support for asthma care in schools.[24 25]

Building on the findings of the~~a~~ qualitative study ~~of the views of primary school children with asthma and their parents~~[24 25], we ~~therefore~~ aimed to explore the views of school staff, healthcare professionals and policymakers about asthma and school-based asthma management to inform the development of an intervention on~~a~~ primary school asthma programme in Malaysia.

For peer review only

Methods

This qualitative study was conducted between May and December 2019 with ethical approval from the Malaysian National Medical Research and Ethics Committee (NMRR-18-3136-44042 (IIR)) and sponsorship from the Academic and Clinical Centre for Research and Development, University of Edinburgh (AC18113). We obtained permission from the schools, the Malaysian Ministry of Health and Ministry of Education to conduct the study. All participants provided their written informed consent.

Context and sampling

About 98% of Malaysia's primary education takes place in government schools under the Ministry of Education.[26] In these schools, there are no school nurses and the healthcare of school children is provided by ~~the school health team under~~ the Ministry of Health in a form of a school health team comprised of family medicine specialists, medical officers and nurses, who provides general health screening and education at each district.[23] We conducted the study in the central region of Peninsular Malaysia and ~~We~~ purposively sampled school staff (teachers and administrative staff) from five government schools (~~2~~ two Malay, ~~2~~ two Chinese and ~~1~~one Indian), to represent the three main ethnic groups and diverse cultures in Malaysia.[26] We also purposively invited participants from a non-governmental organisation and a private school, healthcare professionals including the school health team and policymakers to obtain views from the wider education community.

For peer review only

For peer review only - http://bmjopen.bmj.com/site/about/guidelines.xhtml

Data collection

We arranged focus groups to enable peer interaction supplemented with individual interviews to accommodate the availability and preference of participants.[27 28] We collected basic socio-demographic data (Supplementary 1) and developed piloted semi-structured topic guides (Supplementary 2) based on the biopsychosocial model and previous literature to stimulate discussion.[24 25 29 30] The core topics addressed were asthma experience and awareness, current school asthma management practices and ideas for improvement. All the interviews and focus groups were conducted either in Malay (the national language of Malaysia) or English and moderated by SNR, a researcher who is proficient in both languages. We anticipated up to 50 participants and concluded data collection when there were no new themes emerging based on our core topics. The focus groups and interviews were audio-recorded, transcribed verbatim and analysed using Nvivo software version 11.

Language and translation

The transcripts were analysed in the source language to reduce loss of meaning with translation. Aided by the field notes, the Malay transcripts were translated to English by experienced bilingual translators, and SNR checked the translated transcripts for semantic and contextual equivalence. The translated transcripts were used for data sharing, e.g., the use of quotes to ~~describe~~ illustrate the findings in this report.

Data analysis

We used inductive thematic analysis to explore the views of the participants about asthma and school asthma management. It is a systematic but flexible approach that~~which~~ is often~~may be~~ used to inform intervention development and evaluation.[27 31]

For peer review only - http://bmjopen.bmj.com/site/about/guidelines.xhtml

SNR read the transcripts multiple times for data familiarisation and developed initial codes according to the research question. Constant comparison and merging of initial codes ~~-~~allowed the development of sub-codes, sub-themes and ~~of~~ themes, and we then constructed a schema to describe the relationship between themes. Memos, field notes and discussions with the research team aided data interpretation.

Reflexivity and interpretation

SNR who undertook data collection and coding, is an academic family physician with more than 15 years of working experience in Malaysia. At the beginning of the focus groups/interviews, SNR explained that the main purpose of the study ~~, which~~ was to improve the care of children with asthma~~-~~. She~~and~~ emphasised her position as a researcher in understanding participants' views without judgements. The analysis and results were discussed with the multi-cultural team consisting of primary care physicians and a paediatrician from the UK and Malaysia, to ensure balanced interpretation. We used a pragmatic and constructivist approach but constantly reminded ourselves to be open to any possible emerging theory.

Patient and Public Involvement

We piloted the topic guides among teachers and health professionals when designing the study. They provided feedback on the approach and questions in the topic guides. We shared the preliminary findings of our study with some participants and other stakeholders in stakeholder engagement meetings.

For peer review only

Results

We recruited 52 participants consisting of 40 school staff (including three from a private school), nine healthcare professionals (five from the school health team), two policymakers and a representative from the teachers' professional association. We conducted nine focus groups (up to six participants for each group; ~~and~~ duration up to 40 minutes) and 11 individual interviews (duration up to 45 minutes). Table 1 shows the characteristics of the participants and Table 2 details the participants in each focus group.~~.~~ The participants' age ranged from 23 to 58 years and their working experience ranged 1 to 35 years. A quarter of them (14) had a personal history of asthma or a family member with asthma.

Table 1: Demographic characteristics of participants (N=52)

Demographic	n (years (mean))
Age range: years (mean)	23-58 (42)
Female<br>Male	46<br>6
Working experience: years (mean)	1-35 (17)
Education status<br>Primary/Secondary<br>Tertiary	 0<br>52
Employment background<br>Healthcare professional<br>School staff<br>Policymaker<br>Teacher's professional society	 9<br>40<br>2<br>1
Ethnicity<br>Malay<br>Chinese<br>Indian	 20<br>18<br>14

For peer review only

For peer review only - http://bmjopen.bmj.com/site/about/guidelines.xhtml

Focus group	Participants
1	5 teachers
2	5 teachers
3	4 nurses and 1 nurse assistant
4	3 teachers and 1 clerk
5	4 teachers
6	6 teachers
7	3 teachers and 1 clerk
8	6 teachers
9	2 school nurses and 1 school administrator

Background of participants	
Personal history of asthma	8
Family history of asthma	11
Certified healthcare professional	13

Table 2: Details of participants in each focus group.

For peer review only

Summary of findings

Figure 1 illustrates the inter-relationship of the themes and subthemes~~ of the participants' views of asthma and school-based asthma programme~~. Four themes were constructed to inform the development of a school asthma programme; the first ~~theme~~ highlighted the problems perceived by participants, the second reported the suggested solutions and the two following themes addressed the challenges and enablers.

Quotes describing the themes are provided in text boxes and additional quotes are provided in Tables 3~~2~~ and 4~~3~~.

Problems

Variable awareness of asthma

The awareness of asthma among the participants depended on their personal experience of having asthma or helping others to self-manage asthma, e.g., family members or students, and/or if they had received asthma training or education (Table 3~~2~~). Participants with experience or training could describe symptoms and were familiar with the treatment for asthma symptoms/attacks. Other participants were less able to contribute to the discussion about asthma symptoms, treatment, and possible complications. They had several misconceptions, e.g., 'children with asthma cannot do~~must limit their~~ physical activities~~y~~', and ‘attacks do not occur suddenly in a well child'~~asthma attacks can be anticipated~~'.

> *"Moderator: What happens if the kid has asthma, but he/she says that he/she can do it (physical activities)?*
> *C2: We don’t let them do it.” (FGD2_SS, school staff)*

For peer review only - http://bmjopen.bmj.com/site/about/guidelines.xhtml

E2: I think if the child is having asthma (symptoms), it's best for the parents to bring their child to see the doctor. Can an asthma attack suddenly occur when the child is well?
Moderator: Yes.
E2: I don't know about that." (FGD2_SS, school staff)

Table 3: Additional quotes in themes illustrating problems

**Variable asthma awareness**
"As for me, I would observe them, cool them down since I don't have any experience. Basically, I lacked the knowledge on how to treat asthma. So, that's the only way I can help." (RO4_FGD4_SS, school staff)
"At night if I cough, I will take the blue inhaler and usually, if I use the inhaler and it doesn't work... I will go to the emergency department to take nebuliser." (A14_FGD 4_SS, school staff with asthma)
**Reliance on generic medical school plans**
"If the parents contacted me and said that my child can use this and know how to use this, I will tell the parents that it is the parents' and the child's responsibility to use the medication... You brought it to school and use it on your own. If a child has a more serious condition, we will allow the parents to enter the school to give medication according to the timing." (E2_FGD 2_SS, teacher)
"A5: Sometimes, when we give treatment to a student.<br>*C5: parents blamed the teacher.*<br>*HA5: we are only afraid that something worse can happen. For example, we give medication, suddenly the child's condition gets worse." (FGD 5_SS, teachers)*
**No communicated individual asthma action plan**
"I have a student with asthma, her mother said she has an inhaler, but she does not bring it to school. I've never seen it." (D_FGD 1, teacher)

Reliance on generic medical school plans

For peer review only - http://bmjopen.bmj.com/site/about/guidelines.xhtml

All participants stated that there was no specific asthma school plan in government schools (Table 3~~2~~), so that staff ~~. The c~~Current practice ~~followed~~ followed the general circulars and their generic school plan for any illness or medical emergency. ~~In a situation when~~If a child was unwell, teachers would contact the parents to fetch them~~ child~~ from school, and the parents decided to either treat the child ~~on their own~~themselves or to seek medical attention. In an emergency, teachers would take the child for medical care if it was clear a child needed urgent treatment, or their parent was taking too long to get to school.

> *"Usually what we do, first, is to call their parents. If their parents said, "okay, can bring them to the clinic". Then we will bring them to the nearest clinic. We will get a permission letter from the office." (A5_FGD 5_SS, teacher)*

For peer review only

The teachers were unclear about their role in schoolchildren's health. The circulars stated that staff members were not allowed to give medication to students, but this was interpreted differently according to their personal views and the school management plan. Some teachers interpreted the circulars to only apply to non-prescribed medication and would act *in loco parentis* and give treatment to children only if they had clear instructions from their parents. A few teachers felt able to extend this to supervising prescribed medication such as an inhaler (albeit not being sure of the technique). Other teachers would not want to handle medication at all as they viewed medication use as the responsibility of ~~as the~~ parents~~als~~ and children~~'s'~~ ~~responsibility and would not want to handle medication at all~~. They feared their action might worsen the student's condition, e.g., giving the wrong dose, ~~developing~~ causing side effects of medication, being blamed by parents as they were not trained to manage medical conditions.

For peer review only - http://bmjopen.bmj.com/site/about/guidelines.xhtml

> *"Sometimes parents called the teachers and said, "my child has an inhaler in his baeg. Please can you supervise him"... Usually children will know how to use it. We just supervise when he is using it." (SA6_FGD 6_SS, teacher)*

No communicated individual asthma action plans

The absence of a specific asthma school plan and lack of clarity about the health role of teachers could delay treatment during asthma attacks. Teachers were unsure of what to do when dealing with children with asthma symptoms, some *'panicked'* while several teachers sought help from colleagues considered to have better skills in managing asthma. Both the teachers and the school health team observed that many children with asthma did not bring their reliever inhaler to school. Without a reliever inhaler, teachers tried different strategies when their students had symptoms, e.g., relaxation and breathing techniques while waiting for the parents to arrive.

> *"I was shocked when she was breathless; I am not sure how to help her. I patted her back and massaged her a bit, she was better, but after that, she coughed, and I panicked and quickly called her mother." (D1_FGD 1, teacher)*

Participants identified some poor asthma self-management practices on the part of parents. Teachers observed that parents rarely approached the school regarding their child's illness and ~~only a~~ few had shared their expectations~~/~~ and plans with teachers to support self-management of their child's illness at school (Table 3). Even when children had brought an inhaler to school, the teachers had not been asked to supervise ~~the~~ its use.

> *"The child I told you earlier, his parents did not inform us... Only two parents in this school informed us about their child's illness." (HA5_IDI5, teacher)*

Suggested solutions

For peer review only - http://bmjopen.bmj.com/site/about/guidelines.xhtml

Participants, particularly those with asthma experience and training, identified a need for a school asthma programme to deliver asthma education and introduce an asthma school plan (Table 4~~3~~). On the other hand, participants without asthma experience or training initially thought a school asthma programme was unnecessary, though some ~~participants~~ changed their views during the focus group discussions as they became aware of the risk of asthma attacks occurring at school.

> *"Asthma is not common in school. So, I don't think it (asthma programme) is necessary. But during this discussion, I think teachers should know how to help children with asthma if a child has an attack. It's better." (AN6_FGD 6, a teacher without asthma experience)*

To improve the self-management of children with asthma, healthcare professionals and some teachers suggested the school programme could target the children, their parents and teachers as primary school children were not capable of self-managing asthma independently. The children could be taught to improve their self-management skills e.g., how to avoid asthma triggers, monitor their symptoms, use a reliever inhaler, and get help if having asthma symptoms/attacks. To support the self-management of the children, their parents and teachers could receive similar asthma education.

> *"The child themselves need to know, the severity of the symptoms,... the parents should know that the child has asthma... in the school, in case of any emergency, the teachers must know the child's asthma condition and how severe the asthma is" (SA4_IDI4, health policymaker)*

Participants believed good communication with parents would help school staff to support a child's self-management. Prior agreement on emergency asthma school plans could help boost teachers' confidence in dealing with an attack and protect them from being blamed. A supporting letter from a healthcare professional regarding a child's asthma condition and individual asthma action plan was suggested as another way to provide the information to schools. A few healthcare professionals viewed

For peer review only

schools as a platform to bridge communication with parents who did not bring their child for routine reviews.

> *"We need cooperation from the parent... parents should inform a teacher that 'my child has asthma and need this treatment'. So, the teacher will tell the other teachers and take care of the child... If parents provided a black and white instruction, that is even better." (T6_IDI 6, teacher)*

Table 4: Additional quotes illustrating themes for solutions, challenges and enablers.

**Suggested solutions**
"For improvement, we should ask parents to provide the asthma book together with their immunisation records. So, we can monitor their asthma appointment." (FA3_FGD 3, school health team)
**Implementation challenges**
"We have many programmes, like the health-promoting programme, vaccination programme, the teacher also requested us to finish as soon as possible because they had to go through their education programme." (K2_IDI 2, school health team)
**Implementation enablers**
"It has to be concise and simple. I think half an hour to an hour is enough. More than that, they (children) won't be able to concentrate." (AN6, F.G.D. 6, teacher)
"SA6/RA 6: Malay can understand.<br>AN6: Even if they cannot understand, a teacher will help to explain in Tamil. No problem." (F.G.D. 6, teachers)
"We have a parent and community organisation, and the school parent and teacher organisation who can help to promote the programme. Parents can know about the programme from them, and they can come." (T6_IDI 6, teacher)

Implementation challenges

For peer review only - http://bmjopen.bmj.com/site/about/guidelines.xhtml

Teachers and members of the school health team were quite negative about school asthma programmes because of the workload (Table 43). Teachers were already burdened with academic responsibilities and thought an intensive programme might affect children's learning and general education. The school health team had similar concerns and already felt overwhelmed by their workload, having to provide vaccination, screenings, and general health education in all the schools in the district.

> *"Cannot, we have a lot of programmes. There is no time to allocate in our schedule. A programme like this, can only be done infrequently." (SA6_FGD6, teacher)*

Teachers needed to recognise asthma symptoms/attacks and provide first aid asthma treatment, but the parents of children with asthma needed to take responsibility for self-management of their children. Parental participation in the programme was thus considered crucial but parents' attendance at school health activities was often very poor either because of work commitments or because they perceived that the health programme was not important for them.

> *"The lower education group… maybe they are not financially capable, care less about health, maybe work is a priority. So, if we invite parents to come, this group may not have time to attend." (A2_FGD 2, teacher)*

The target groups for the school asthma programme have different needs and capabilities. Primary schools covered a wide age range (7-12years) and the teachers' experience was that lessons needed to be customised to the child's educational level. Some teachers suggested the programme should be delivered in the native language of the children (including Tamil or Chinese as opposed to just the national language) for better understanding.

> *"HA7: English, Tamil can.*

For peer review only

For peer review only - http://bmjopen.bmj.com/site/about/guidelines.xhtml

> *VA7: But if it is in Tamil, they will understand better, because it is their native language. TA7: but some lower and upper primary can understand Malay." (F.G.D. 7, teachers)*

Implementation enablers

Participants suggested simple, engaging, and tailored approaches catering for busy schedules, and different individual needs and capabilities (Table 43). The delivery ought to be fun and appealing to children and adults. They suggested quizzes, demonstrations, and the use of digital media such as videos to assist sessions' delivery.

> *"We can use visual aids, we teach first. After that, we ask them to demonstrate. Something like a simulation, if this happened, what will you do?" (NO7_IDI 7, teacher)*

Healthcare professionals considered good partnerships with schools as an essential enabler to promoting the uptake of school asthma programmes. The school health team had a long-term relationship with the schools and suggested various approaches to building the partnership including involvement of the school administrators at the early stage of the programme development. A good partnership with schools also helped identify and address implementation issues, for example, teachers from Chinese/Tamil schools volunteered to translate the intervention to help children who were not able to understand Malay.

> *"We have to hold a meeting with the headmaster and the teacher-in-charge of health... once the administrators are interested, they will suggest to you the teacher who will be in charge of the programme... So, you will work with the teacher to develop and run the programme." (RO_FGD 3, school health team)*

All participants proposed that healthcare professionals should deliver the intervention in schools, ideally as a multidisciplinary team including pharmacists and paediatricians. To increase parents' participation, teachers suggested wider community involvement such as parent-teacher associations, community

For peer review only

organisations, and non-governmental organisations to help promote the intervention. Policymakers highlighted the need for introducing a national asthma school plan to improve asthma care of children during attacks.

> *"I think this matter will need a clear S.O.P. (standard operating procedure)... Although we have experience with our children, (but) for us to apply the same thing to someone's children, we are worried." (N08_IDI8, education policymaker)*

Discussion

Summary of findings

Asthma awareness and experience of managing asthma was limited among the school staff and, with no specific asthma school plan to provide guidance, treatment during asthma symptoms/emergencies could be inappropriate or delayed. School health circulars were not clear about the teachers' health role in schools. To improve this, participants suggested a school asthma programme that~~which~~ focused on introducing an asthma school plan and improving awareness about asthma amongst children, their parents and teachers. Communication between healthcare professionals, parents and the school also needed improvement. The participants anticipated heavy workload, poor parental participation and diverse individual capabilities and needs as challenges to implementing school asthma programmes. Tailored approaches, good partnership with schools and multi-organisation involvement were discussed as enablers to develop and deliver the programme. We will use these findings to inform the development of a school-based asthma programme in Malaysia.

For peer review only

For peer review only - http://bmjopen.bmj.com/site/about/guidelines.xhtml

Study findings in comparison with other studies

Our interviewees suggested developing a school asthma programme involving parents and teachers, that focussed on asthma awareness, and an asthma school plan for use in attacks. Similar community-wide programmes have been implemented in other contexts.[32 33] Education for staff and children could correct common misperceptions about asthma and asthma medication and involvement of school health teams could improve the accessibility of healthcare for children with asthma.[19 34-36] School asthma programmes offer the opportunity to focus self-management education on children and involve children's social network to support their self-management skills and practices.[37 38]

The lack of awareness among school staff about which students had asthma, combined with poor understanding of symptoms and first aid treatment leads to hesitancy and delay in taking action risking deterioration or even death.[39 40] Delineating the health roles of teachers in an asthma school plan and providing adequate training for school staff could improve their confidence and prevent treatment delay.[16 40] However, such strategies rely on parents providing asthma reliever medication; without this, school staff will have to call parents or get immediate medical help.[39 41] School asthma guidelines in Australia, Canada, ~~the~~ United Kingdom and ~~the~~ United States encourage inhalers to be labelled and kept with the child or the class teacher in primary schools without a school nurse.[7 42-44] These guidelines also recommend that schools should have an emergency asthma kit to include a reliever inhaler, spacer and agreed information on when and how to use it.[7-9 43] Establishing immediate access to an emergency reliever inhaler in schools required high-level policy changes and detailed guidance for using first aid asthma treatment.[43 45]

For peer review only

Therefore, a clear guideline to inform stakeholders e.g. policymakers, school leaders, parents and the community will aid the development and implementation of school asthma programmes.

Highlighting the importance of the programmes to stakeholders particularly policymakers could ensure successful implementation of school asthma programmes. Policy supporting improvement in school asthma management is a powerful tool in prioritising and providing resources to assist implementation. A good partnership with schools throughout the development of asthma school plans and programmes is crucial to identify challenges and enablers for successful implementation.[46,47] The presence of school plans does not guarantee teachers are equipped with the skills to manage asthma symptoms/attacks.[48] Teachers need to be aware of the school plan, and receive education and training in use of reliever treatment to equip them to handle asthma events in school.[48,49] Tailoring designs according to the needs and capabilities of individuals and schools could improve the uptake and mitigate the challenges of the programmes.[19,50] Healthcare providers play a crucial role in the partnership. They need to provide personalised self-management education to a child including assessing if the child can self-carry and self-administer their medication. They can also provide a personalised asthma action plan to be shared with the school.[5,45] Involving the wider community, e.g. parent and teacher association, health care providers and social services may support delivery and sustainability of school asthma programmes.[19,51,52]

For peer review only

Study strengths and limitations

This is one of a few studies on school asthma programmes conducted in low-middle income countries. Flexible time, venue and data collection methods facilitated recruitment of a study population with various backgrounds which contributed to the diverse views of participants. Nevertheless, the findings may not be wholly applicable in other settings, and an intervention based on our findings will need adaptation for other contexts. Memos, field notes and ~~team~~ multidisciplinary team discussions aided analysis and ~~multidisciplinary team discussions~~ informed interpretation.

Some loss of meaning may have occurred during the translation of Malay quotes; however, we analysed the data in the source language and checked the semantic and context equivalence for translated quotes. Thematic analysis may reduce the interpretive details of each participant' as the coding process focuses on the content, and less on the processes and phenomena~~on~~ within individuals. However, we used a pragmatic, inductive approach with constant comparisons of data and discussions to inform our understanding of the views expressed. We were aware of the risk of researcher bias in qualitative research, and actively discussed reflexivity~~,~~ in the regular multidisciplinary team discussions which aided analysis and informed interpretation. Participants with less experience on the topic may have contributed less to the focus groups though they were provided with opportunities to provide their views.

For peer review only

For peer review only - http://bmjopen.bmj.com/site/about/guidelines.xhtml

Conclusions

School asthma programmes could improve the care and support for children with asthma. -Aligned with the WHO recommendations of Global -Sschool Health Initiative recommendationsa review of WHO school health initiative[1], the participants in our study highlighted the importance of tailored approaches, a good partnership with schools and engaging the wider community at an early stage of developing a school asthma programme. There is a need for a clear asthma guideline on school-based programmes to inform policymakers, school leaders, and the community in Malaysia.

For peer review only

Data Availability

Due to ethical concerns, supporting data cannot be made openly available and are stored under restricted access in the University of Edinburgh DataVault. Further information about the data and conditions for access are available via the University of Edinburgh Research Explorer [https://doi.org/10.7488/191f40bd-ace9-4c8a-a36c-43b796303166~~we are in the process of depositing the data and the DOI will be provided here~~]. Additional non-disclosive details relating to other aspects of the data are openly available from the University of Edinburgh DataShare repository at [https://doi.org/10.7488/ds/3004~~we are in the process of depositing the data and the DOI will be provided here~~].

Acknowledgements

We would like to acknowledge all the participants who voluntarily participated in this research and the local authorities for their support during the study. The RESPIRE collaboration comprises the UK Grant holders, Partners and research teams as listed on the RESPIRE website (www.ed.ac.uk/usher/respire), including Nik Sherina Hanafi and Norita Binti Hussein.

Funding

This research was commissioned by the UK National Institute for Health Research (NIHR) Global Health Research Unit on Respiratory Health (RESPIRE), using UK Aid from the UK Government. The views expressed in this publication are those of the author(s) and not necessarily those of the NIHR or the UK Department of Health and Social Care.

Competing Interest

None declared.

Author contributions

All authors (SNR, EMK, SML, SC and HP) contributed to the study conception. SNR performed the data collection, checking and coded the transcripts. All authors (SNR, EMK, SML, SC and HP) -contributed to data analysis and interpretation of data. SNR drafted the manuscript and all authors (SNR, EMK, SML, SC and HP) provided critical revisions and editing of the manuscript.

References

1. World Health Organisation. Making every school a health--promoting school. Geneva, Switzerland, 2021.
2. World Health Organization. Global School Health Initiatives: achieving health and education outcomes. Report of a meeting, Bangkok,Thailand, 23–25 November 2015. . Geneva, Switzerland: World Health Organisation, 2017.
3. Levinson J, Kohl K, Baltag V, et al. Investigating the effectiveness of school health services delivered by a health provider: A systematic review of systematic reviews. *PLoS One* 2019;14(6):e0212603. doi: 10.1371/journal.pone.0212603 [published Online First: 2019/06/13]
4. Global Asthma Network. The Global Asthma Report. Auckland, New Zealand, 2018.
5. Global Initiative for Asthma. Global Strategy for Asthma Prevention, 2021.
6. Scottish Intercollegiate Guidelines Network/British Thoracic Society. SIGN 158 British guideline on the management of asthma. London: Scottish Intercollegiate Guidelines Network/British Thoracic Society, 2019.
7. Asthma Australia. Asthma Guidelines for Queenlands schools Australia: Asthma Australia, 2019.
8. Healthy London Partnership. London schools' guide for the care of children and young people with asthma: Pre-school, primary and secondary school years: London, 2016.

For peer review only

9. U.S. Department of Health and Human Services. Managing Asthma: A guide for schools: National Institutes of Health, 2014.

10. Piot P, Caldwell A, Lamptey P, et al. Addressing the growing burden of non-communicable disease by leveraging lessons from infectious disease management. *J Glob Health* 2016;6(1):010304-04. doi: 10.7189/jogh.06.010304

11. World Health Organisation. WHO guideline on school health services. . Geneva, 2021.

12. Harris K, Kneale D, Lasserson TJ, et al. School-based self-management interventions for asthma in children and adolescents: a mixed methods systematic review. *Cochrane Database Syst Rev,* 2019;1(1):CD011651. doi: 10.1002/14651858.CD011651.pub2 [published Online First: 2019/01/29]

13. Walter H, Sadeque-Iqbal F, Ulysse R, et al. Effectiveness of school-based family asthma educational programs in quality of life and asthma exacerbations in asthmatic children aged five to 18: a systematic review. *JBI Database System Rev Implement Rep* 2016;14(11):113-38. doi: 10.11124/JBISRIR-2016-003181 [published Online First: 2016/12/13]

14. Isik E, Fredland NM, Freysteinson WM. School and Community-based Nurse-led Asthma Interventions for School-aged Children and Their Parents: A Systematic Literature Review. *J Pediatr Nurs* 2019;44:107-14. doi: 10.1016/j.pedn.2018.11.007 [published Online First: 2019/01/27]

15. Al Aloola NA, Naik-Panvelkar P, Nissen L, et al. Asthma interventions in primary schools--a review. *J Asthma* 2014;51(8):779-98. doi: 10.3109/02770903.2014.914534 [published Online First: 2014/04/16]

16. Kew KM, Carr R, Donovan T, et al. Asthma education for school staff. *Cochrane Database Syst Rev,* 2017;4:CD012255. doi: 10.1002/14651858.CD012255.pub2 [published Online First: 2017/04/13]

17. Craig P, Dieppe P, Macintyre S, et al. Developing and evaluating complex interventions: the new Medical Research Council guidance. *Int J Nurs Stud* 2013;50(5):587-92. doi: 10.1016/j.ijnurstu.2012.09.010 [published Online First: 2012/11/20]

18. Wight D, Wimbush E, Jepson R, et al. Six steps in quality intervention development (6SQuID). *J Epidemiol Community Health* 2016;70(5):520-5. doi: 10.1136/jech-2015-205952 [published Online First: 2015/11/18]

19. Cicutto L, Gleason M, Haas-Howard C, et al. Building Bridges for Asthma Care Program: A School-Centered Program Connecting Schools, Families, and Community Health-Care Providers. *J Sch Nurs,* 2020;36(3):168-80. doi: 10.1177/1059840518805824 [published Online First: 2018/10/20]

20. Ahad A, Khoo EM. Asthma Control and Care Among Malaysian Primary School Children: A Cross-Sectional Study. *Asia Pac J Public Health* 2017;29(5):422-29. doi: 10.1177/1010539517717766 [published Online First: 2017/07/19]

21. Asher MI, Montefort S, Bjorksten B, et al. Worldwide time trends in the prevalence of symptoms of asthma, allergic rhinoconjunctivitis, and eczema in childhood: ISAAC Phases One and Three repeat multicountry cross-sectional surveys. *Lancet (London, England)* 2006;368(9537):733-43. doi: 10.1016/S0140-6736(06)69283-0

22. Wong GW, Kwon N, Hong JG, et al. Pediatric asthma control in Asia: phase 2 of the Asthma Insights and Reality in Asia-Pacific (AIRIAP 2) survey. *Allergy* 2013;68(4):524-30. doi: 10.1111/all.12117 [published Online First: 2013/02/19]

For peer review only - http://bmjopen.bmj.com/site/about/guidelines.xhtml

23. Kementerian Kesihatan Malaysia. Garis Panduan Perkhidmatan Kesihatan Sekolah. Malaysia, 2013.
24. Ramdzan SN, Pinnock H, Liew SM, et al. Perceptions of complementary/alternative medicine use and influence on evidence-based asthma medicine adherence in Malaysian children. *NPJ Prim Care Respir Med* 2019;29(1):5. doi: 10.1038/s41533-019-0118-x [published Online First: 2019/02/26]
25. Ramdzan SN, Khoo EM, Liew SM, et al. How young children learn independent asthma self-management: a qualitative study in Malaysia. *Arch Dis Child* 2020;105(9):819-24. doi: 10.1136/archdischild-2019-318127 [published Online First: 2020/07/06]
26. Malaysia Education Statistics. Quick Facts 2018. Malaysia: Educational Planning and Research Division, Ministry of Education Malaysia, 2018.
27. Braun V, Clarke V. Succesful Qualitative Research: a practical guide for beginners. London: SAGE Publication Ltd 2013.
28. Silverman D. Doing Qualitative Research. 5th edition ed. London, United Kingdom: SAGE Publication Limited 2018.
29. Farre A, Wood V, McDonagh JE, et al. Health professionals' and managers' definitions of developmentally appropriate healthcare for young people: conceptual dimensions and embedded controversies. *Arch Dis Child* 2016;101(7):628-33. doi: 10.1136/archdischild-2015-309473 [published Online First: 2016/03/06]
30. Borrell-Carrio F, Suchman AL, Epstein RM. The biopsychosocial model 25 years later: principles, practice, and scientific inquiry. *Ann Fam Med* 2004;2(6):576-82. doi: 10.1370/afm.245 [published Online First: 2004/12/04]
31. Grbich C. Qualitative Data Analysis: An introduction. Second ed. United Kingdom: SAGE Publication 2013.
32. Lenney W, Bush A, Fitzgerald DA, et al. Improving the global diagnosis and management of asthma in children. *Thorax* 2018;73(7):662-69. doi: 10.1136/thoraxjnl-2018-211626
33. Szefler SJ, Fitzgerald DA, Adachi Y, et al. A worldwide charter for all children with asthma. *Pediatr Pulmonol* 2020;55(5):1282-92. doi: 10.1002/ppul.24713 [published Online First: 2020/03/07]
34. Suwannakeeree P, Deerojanawong J, Prapphal N. School-Based Educational Interventions Can Significantly Improve Health Outcomes in Children with Asthma. *J Med Assoc Thai* 2016;99(2):166-74. [published Online First: 2016/06/03]
35. Clark NM, Gong M, Kaciroti N, et al. A trial of asthma self-management in Beijing schools. *Chronic illness* 2005;1(1):31-8. doi: 10.1177/17423953050010010101 [published Online First: 2006/12/02]
36. Carvalho Coelho AC, Barretto Cardoso LS, de Souza-Machado C, et al. The Impacts of Educational Asthma Interventions in Schools: A Systematic Review of the Literature. *Can Respir J,* 2016;2016:8476206. doi: 10.1155/2016/8476206 [published Online First: 2016/09/23]
37. Szefler SJ, Cloutier MM, Villarreal M, et al. Building Bridges for Asthma Care: Reducing school absence for inner-city children with health disparities. *J Allergy Clin Immunol* 2019;143(2):746-54 e2. doi: 10.1016/j.jaci.2018.05.041 [published Online First: 2018/07/29]
38. Lemanske RF, Jr., Kakumanu S, Shanovich K, et al. Creation and implementation of SAMPRO: A school-based asthma management program. *J Allergy Clin*

For peer review only - http://bmjopen.bmj.com/site/about/guidelines.xhtml

Immunol 2016;138(3):711-23. doi: 10.1016/j.jaci.2016.06.015 [published Online First: 2016/09/07]
39. Greiling AK, Boss LP, Wheeler LS. A preliminary investigation of asthma mortality in schools. *J Sch Health,* 2005;75(8):286-90. doi: 10.1111/j.1746-1561.2005.00039.x [published Online First: 2005/09/24]
40. Murphy KR, Hopp RJ, Kittelson EB, et al. Life-threatening asthma and anaphylaxis in schools: a treatment model for school-based programs. *Ann Allergy Asthma Immunol* 2006;96(3):398-405. doi: 10.1016/S1081-1206(10)60906-4 [published Online First: 2006/04/07]
41. Gerald JK, Stroupe N, McClure LA, et al. Availability of Asthma Quick Relief Medication in Five Alabama School Systems. *Pediatr Allergy Immunol Pulmonol* 2012;25(1):11-16. doi: 10.1089/ped.2011.0118 [published Online First: 2012/03/29]
42. Council on School H. Policy statement--guidance for the administration of medication in school. *Pediatrics* 2009;124(4):1244-51. doi: 10.1542/peds.2009-1953 [published Online First: 2009/09/30]
43. Department of Health. Guidance on the use of emergency salbutamol inhalers in schools. Storage and care of the inhaler. United Kingdom, 2015.
44. OPHEA Health schools and Healthy Communities. Creating Asthma friendly schools. Canada, 2015.
45. Toups MM, Press VG, Volerman A. National Analysis of State Health Policies on Students' Right to Self-Carry and Self-Administer Asthma Inhalers at School. *J Sch Health,* 2018;88(10):776-84. doi: 10.1111/josh.12681 [published Online First: 2018/09/12]
46. Cicutto L, Gleason M, Szefler SJ. Establishing school-centered asthma programs. *J Allergy Clin Immunol* 2014;134(6):1223-30. doi: 10.1016/j.jaci.2014.10.004 [published Online First: 2014/12/09]
47. Kakumanu S, Lemanske RF, Jr. Asthma in Schools: How School-Based Partnerships Improve Pediatric Asthma Care. *Immunol Allergy Clin North Am* 2019;39(2):271-81. doi: 10.1016/j.iac.2018.12.011 [published Online First: 2019/04/08]
48. Jaramillo Y, Reznik M. Do United States' teachers know and adhere to the national guidelines on asthma management in the classroom? A systematic review. *The Scientific World Journal* 2015;2015:624828. doi: 10.1155/2015/624828 [published Online First: 2015/03/03]
49. Cain A, Reznik M. Asthma management in New York City schools: A classroom teacher perspective. *J Asthma* 2016;53(7):744-50. doi: 10.3109/02770903.2015.1135946 [published Online First: 2016/04/01]
50. Brigden A, Parslow RM, Linney C, et al. How are behavioural interventions delivered to children (5-11 years old): a systematic mapping review. *BMJ Paediatr Open* 2019;3(1):e000543. doi: 10.1136/bmjpo-2019-000543 [published Online First: 2020/01/08]
51. McCabe EM, Kaskoun JR, Murphy EL, et al. An Evidence-Based Framework for Implementation of a School-Community Partnership. *NASN Sch Nurse* 2021;36(3):164-69. doi: 10.1177/1942602X20964765 [published Online First: 2020/10/16]
52. Clark NM, Lachance L, Doctor LJ, et al. Policy and system change and community coalitions: outcomes from allies against asthma. *American journal of public health* 2010;100(5):904-12. doi: 10.2105/AJPH.2009.180869 [published Online First: 2010/03/18]

For peer review only - http://bmjopen.bmj.com/site/about/guidelines.xhtml

Figure legend

Figure 1: Themes and subthemes of participants' views of asthma and school-based asthma intervention.

For peer review only

Stakeholders' views of supporting asthma management in schools with a school-based asthma programme for primary school children: A qualitative study in Malaysia

Journal:	*BMJ Open*
Manuscript ID	bmjopen-2021-052058.R2
Article Type:	Original research
Date Submitted by the Author:	04-Jan-2022
Complete List of Authors:	Ramdzan, Siti Nurkamilla; Universiti Malaya, Department of Primary Care Medicine; The University of Edinburgh Usher Institute of Population Health Sciences and Informatics, NIHR Global Health Research Unit on Respiratory Health (RESPIRE)<br>Khoo, Ee Ming; Universiti Malaya, Department of Primary Care Medicine<br>Liew, Su May; Universiti Malaya, Department of Primary Care Medicine<br>Cunningham, Steven; The University of Edinburgh Usher Institute of Population Health Sciences and Informatics, NIHR Global Health Research Unit on Respiratory Health (RESPIRE)<br>Pinnock, Hilary; The University of Edinburgh Usher Institute of Population Health Sciences and Informatics, NIHR Global Health Research Unit on Respiratory Health (RESPIRE)
**Primary Subject Heading**:	Health services research
Secondary Subject Heading:	Paediatrics, Respiratory medicine, Public health
Keywords:	Asthma < THORACIC MEDICINE, Paediatric thoracic medicine < PAEDIATRICS, QUALITATIVE RESEARCH, Community child health < PAEDIATRICS, Change management < HEALTH SERVICES ADMINISTRATION & MANAGEMENT

I, the Submitting Author has the right to grant and does grant on behalf of all authors of the Work (as defined in the below author licence), an exclusive licence and/or a non-exclusive licence for contributions from authors who are: i) UK Crown employees; ii) where BMJ has agreed a CC-BY licence shall apply, and/or iii) in accordance with the terms applicable for US Federal Government officers or employees acting as part of their official duties; on a worldwide, perpetual, irrevocable, royalty-free basis to BMJ Publishing Group Ltd ("BMJ") its licensees and where the relevant Journal is co-owned by BMJ to the co-owners of the Journal, to publish the Work in this journal and any other BMJ products and to exploit all rights, as set out in our licence.

The Submitting Author accepts and understands that any supply made under these terms is made by BMJ to the Submitting Author unless you are acting as an employee on behalf of your employer or a postgraduate student of an affiliated institution which is paying any applicable article publishing charge ("APC") for Open Access articles. Where the Submitting Author wishes to make the Work available on an Open Access basis (and intends to pay the relevant APC), the terms of reuse of such Open Access shall be governed by a Creative Commons licence – details of these licences and which Creative Commons licence will apply to this Work are set out in our licence referred to above.

Other than as permitted in any relevant BMJ Author's Self Archiving Policies, I confirm this Work has not been accepted for publication elsewhere, is not being considered for publication elsewhere and does not duplicate material already published. I confirm all authors consent to publication of this Work and authorise the granting of this licence.

For peer review only

Stakeholders' views of supporting asthma management in schools with a school-based asthma programme for primary school children: A qualitative study in Malaysia

Siti Nurkamilla Ramdzan, MMed[1,2], Ee Ming Khoo, MD[1], Su May Liew, DPhil[1], Steve Cunningham, PhD[2], Hilary Pinnock, MD*[2] on behalf of RESPIRE collaborators

[1]*Department of Primary Care Medicine, Faculty of Medicine, University of Malaya, Malaysia*

[2]*NIHR Global Health Research Unit on Respiratory Health (RESPIRE), Usher Institute, University of Edinburgh, United Kingdom*

Address for correspondence
*Professor Hilary Pinnock
NIHR Global Health Research Unit on Respiratory Health (RESPIRE),
Usher Institute,
The University of Edinburgh.
Doorway 3, Medical School, Teviot Place,
Edinburgh EH8 9AG, UK.
Tel: 0131 650 9474
Email: Hilary.Pinnock@ed.ac.uk

Main text word count: 4023 (excluding abstract, main headings, tables, references, and additional information, e.g. acknowledgement, funding, competing interest) Maximum: 4000 words

For peer review only

Abstract

Objective: The WHO Global School Health Initiative aimed to improve child and community health through health promotion programmes in schools, though most focus on preventing communicable disease. Despite WHO recommendations, no asthma programme is included in the Malaysian national school health service guideline. Therefore, we aimed to explore the views of school staff, healthcare professionals and policymakers about the challenges of managing asthma in schools and the potential of a school asthma programme for primary school children.

Design: A focus group and individual interview qualitative study using purposive sampling of participants to obtain diverse views. Data collection was guided by piloted semi-structured topic guides. The focus groups and interviews were audio-recorded, transcribed verbatim and analysed using inductive thematic analysis. We completed data collection once data saturation was reached.

Setting: Stakeholders in education and health sectors in Malaysia.

Participants: Fifty-two participants (40 school staff, 9 healthcare professionals and 3 policymakers) contributed to nine focus groups and eleven individual interviews.

Results: School staff had limited awareness of asthma and what to do in emergencies. There was no guidance on asthma management in government schools, and teachers were unclear about their role in school children's health. These uncertainties led to delays in the treatment of asthma symptoms/attacks, and suggestions that an asthma education programme and a school plan would improve asthma care. Perceived challenges in conducting school health programmes included a busy school schedule and poor parental participation. A tailored asthma programme in partnerships with schools could facilitate the programme's adoption and implementation.

For peer review only

For peer review only - http://bmjopen.bmj.com/site/about/guidelines.xhtml

Conclusions: Identifying and addressing issues and challenges specific to the school and wider community could facilitate the delivery of a school asthma programme in line with the WHO School Health Initiative. Clarity over national policy on the roles and responsibilities of school staff could support implementation and guide appropriate and prompt response to asthma emergencies in schools.

Word count: 301 (Maximum: 300)

Keywords: school-based, asthma, complex intervention, low-middle income countries and health behaviour

For peer review only

Strengths and limitations of this study

- One of a few studies on school asthma programmes conducted in low-middle income countries.
- Various backgrounds contributed to the diverse views of participants.
- Some loss of meaning may have occurred during the translation of Malay quotes to English, but the analysis was conducted in the source language.
- Participants with less experience on the topic may have contributed less to the focus groups though they were provided with opportunities to provide their views.

Introduction

The Global School Health Initiative launched by the World Health Organization (WHO) in 1995 aimed to improve child, adolescent and community health through health promotion programmes in schools.[1] Globally, the compulsory school years offer an 'easy entry point' for embedding healthy behaviours at a young age and, by extension, influencing families.[2] Health promotion programmes (e.g. de-worming, vaccination, water, sanitation, hygiene) have been successfully implemented in low-middle income countries (LMICs) through this initiative.[2] Non-communicable diseases (NCDs) are an increasing challenge in all economies, though successful NCD programmes have typically been focussed in high-income countries.[2 3]

Asthma is the commonest NCD in children with a global prevalence of 11.6%.[4] Children with poor asthma control have symptoms of wheeze, cough and difficulty breathing that affect their daily activities.[5] Good self-management of asthma improves symptoms and reduces the risk of attacks,[6] but for primary school children, self-

For peer review only

management of asthma requires support from their social network including their parents, school and teachers.[7] Young children can spend up to half their waking hours in schools, and school staff could help students develop asthma self-management skills.[8-10] Furthermore, attacks can occur unexpectedly, and schools need plans for responding appropriately and promptly to asthma symptoms.[5 11]

The WHO endorses the role of school in supporting children with asthma and recommends that asthma should be an essential school health service in all countries.[3 12 13] School-based asthma self-management educational interventions that were theory-driven, involved parent, included children's satisfaction and done outside children's free time have been shown to improve asthma control, reduce school absenteeism and asthma attacks[14-16], though few were conducted in LMICs.[14 15] These programmes are complex interventions that can be successful if tailored to the target population[17 18], and adapted to the context.[19] The WHO has also highlighted the importance of policy and organisation-level collaboration and engagement with parents, students and teachers.[2]

In Malaysia, an estimated 9% of children have asthma [20 21], at least half of whom are poorly controlled.[20 22] Despite this morbidity, the national school health service guideline does not include an asthma programme[23], and prior qualitative study conducted among primary school children with asthma and their parents revealed a perception of poor support for asthma care in schools.[7 24]

For peer review only - http://bmjopen.bmj.com/site/about/guidelines.xhtml

We therefore aimed to explore the views of school staff, healthcare professionals and policymakers about the challenges of managing asthma in schools and the potential of a school asthma programme for primary school children to improve care.

Methods

This mixed focus group and individual interview qualitative study was conducted between May and December 2019 with ethical approval from the Malaysian National Medical Research and Ethics Committee (NMRR-18-3136-44042 (IIR)) and sponsorship from the Academic and Clinical Centre for Research and Development, University of Edinburgh (AC18113). We obtained permission from the schools, the Malaysian Ministry of Health and the Ministry of Education to conduct the study. All participants provided their written informed consent.

Context and sampling

About 98% of Malaysia's primary education takes place in government schools under the Ministry of Education.[25] In these schools, there are no school nurses and the healthcare of school children is provided by the Ministry of Health in a form of a school health team comprised of family medicine specialists, medical officers and nurses, who provides general health screening and education at each district.[23] We conducted the study in the central region of Peninsular Malaysia and purposively sampled school staff (teachers and administrative staff) from five government schools (two Malay, two Chinese and one Indian), to represent the three main ethnic groups and diverse cultures in Malaysia.[25] We also purposively invited participants from a non-governmental organisation and a private school, healthcare professionals including the school health team and policymakers to obtain views from the wider education

For peer review only

community. Potential participants were invited individually or via their organisation by providing them with a participant information sheet.

Data collection

We arranged focus groups to enable peer interaction supplemented with individual interviews to accommodate the availability and preference of participants.[26 27] We collected basic socio-demographic data, e.g. age, gender, profession and asthma experience (Supplementary 1, with variables reported in table 1), and developed piloted semi-structured topic guides (Supplementary 2) based on the biopsychosocial model and previous literature to stimulate discussion.[7 24 28 29] Different topic guides containing specific questions relevant to different roles were used for different professions. The core topics addressed were asthma experience and awareness, current school asthma management practices and ideas for improvement. All the interviews and focus groups were conducted either in Malay (the national language of Malaysia) or English and moderated by SNR, a researcher who is proficient in both languages. We anticipated up to 50 participants and concluded data collection when there were no new themes emerging based on our core topics. The focus groups and interviews were audio-recorded, transcribed verbatim and analysed using Nvivo software version 11.

Language and translation

The transcripts were analysed in the source language to reduce loss of meaning with translation. Aided by the field notes, the Malay transcripts were translated to English by experienced bilingual translators, and SNR checked the translated transcripts for

For peer review only - http://bmjopen.bmj.com/site/about/guidelines.xhtml

semantic and contextual equivalence. The translated transcripts were used for data sharing, e.g., the use of quotes to illustrate the findings in this report.

Data analysis

We used inductive thematic analysis, a systematic but flexible approach that is often used to inform intervention development and evaluation.[26 30] SNR read the transcripts multiple times for data familiarisation and developed initial codes according to the research question. Constant comparison and merging of initial codes allowed the development of sub-codes, sub-themes and themes, and we then constructed a schema to describe the relationship between themes. Memos, field notes and discussions with the research team aided data interpretation.

Reflexivity and interpretation

SNR who undertook data collection and coding, is an academic family physician with more than 15 years of working experience in Malaysia. At the beginning of the focus groups/interviews, SNR explained that the main purpose of the study was to improve the care of children with asthma. She emphasised her position as a researcher in understanding participants' views without judgements. The analysis and results were discussed with the multi-cultural team consisting of primary care physicians and a paediatrician from the UK and Malaysia, to ensure balanced interpretation. We used a pragmatic and constructivist approach but constantly reminded ourselves to be open to any possible emerging theory.

Patient and Public Involvement

We piloted the topic guides among teachers and health professionals when designing the study. They provided feedback on the approach and questions in the topic guides.

For peer review only

We shared the preliminary findings of our study with some participants and other stakeholders in stakeholder engagement meetings.

Results

We recruited 52 participants consisting of 40 school staff (including three from a private school), nine healthcare professionals (five from the school health team), three policymakers including a representative from the teachers' professional association. We conducted nine focus groups (up to six participants for each group; duration up to 40 minutes) and 11 individual interviews (duration up to 45 minutes). Table 1 shows the characteristics of the participants and Table 2 details the participants in each focus group. The participants' age ranged from 23 to 58 years and their working experience ranged 1 to 35 years. A quarter of them (14) had a personal history of asthma or a family member with asthma.

Table 1: Demographic characteristics of participants (N=52)

Demographic	n (years (mean))
Age range: years (mean)	23-58 (42)
Female	46
Male	6
Working experience: years (mean)	1-35 (17)
Education status	
Primary/Secondary	0
Tertiary	52
Employment background	
Healthcare professional	9
School staff	40
Policymaker	3

For peer review only - http://bmjopen.bmj.com/site/about/guidelines.xhtml

Ethnicity	
Malay	20
Chinese	18
Indian	14
Background of participants	
Personal history of asthma	8
Family history of asthma	11
Certified healthcare professional	13

Table 2: Details of participants in each focus group.

Focus group	Participants
1	5 teachers
2	5 teachers
3	4 nurses and 1 nurse assistant
4	3 teachers and 1 clerk
5	4 teachers
6	6 teachers
7	3 teachers and 1 clerk
8	6 teachers
9	2 school nurses and 1 school administrator

Summary of findings

Figure 1 illustrates the inter-relationship of the themes and subthemes. Four themes were constructed to inform the development of a school asthma programme; the first highlighted the problems perceived by participants, the second reported the suggested solutions and the two following themes addressed the challenges and enablers.

For peer review only

Quotes describing the themes are provided in text boxes and additional quotes are provided in Tables 3 and 4.

Problems

Variable awareness of asthma

The awareness of asthma among the participants depended on their personal experience of having asthma or helping others to self-manage asthma, e.g., family members or students, and/or if they had received asthma training or education (Table 3). Participants with experience or training could describe symptoms and were familiar with the treatment for asthma symptoms/attacks. Other participants were less able to contribute to the discussion about asthma symptoms, treatment, and possible complications. They had several misconceptions, e.g., 'children with asthma cannot do physical activities', and ‘attacks do not occur suddenly in a well child'.

"Moderator: What happens if the kid has asthma, but he/she says that he/she can do it (physical activities)?
C2: We don’t let them do it.” (FGD2_SS, school staff)

E2: I think if the child is having asthma (symptoms), it's best for the parents to bring their child to see the doctor. Can an asthma attack suddenly occur when the child is well?
Moderator: Yes.
E2: I don't know about that.” (FGD2_SS, school staff)

Table 3: Additional quotes in themes illustrating problems

**Variable asthma awareness**
"As for me, I would observe them, cool them down since I don't have any experience. Basically, I lacked the knowledge on how to treat asthma. So, that's the only way I can help." (RO4_FGD4_SS, school staff)
"At night if I cough, I will take the blue inhaler and usually, if I use the inhaler and it doesn't

For peer review only

work... I will go to the emergency department to take nebuliser." (A14_FGD 4_SS, school staff with asthma)
**Reliance on generic medical school plans**
"If the parents contacted me and said that my child can use this and know how to use this, I will tell the parents that it is the parents' and the child's responsibility to use the medication... You brought it to school and use it on your own. If a child has a more serious condition, we will allow the parents to enter the school to give medication according to the timing." (E2_FGD 2_SS, teacher)
"A5: Sometimes, when we give treatment to a student.<br>C5: parents blamed the teacher.<br>HA5: we are only afraid that something worse can happen. For example, we give medication, suddenly the child's condition gets worse." (FGD 5_SS, teachers)
**No communicated individual asthma action plan**
"I have a student with asthma, her mother said she has an inhaler, but she does not bring it to school. I've never seen it." (D_FGD 1, teacher)

Reliance on generic medical school plans

All participants stated that there was no specific asthma school plan in government schools (Table 3), so that staff followed the general advice (e.g. in circular letters from authorities) and their generic school plan for any illness or medical emergency. If a child was unwell, teachers would contact the parents to fetch them from school, and the parents decided to either treat the child themselves or to seek medical attention. In an emergency, teachers would take the child for medical care if it was clear a child needed urgent treatment, or their parent was taking too long to get to school.

> *"Usually what we do, first, is to call their parents. If their parents said, "okay, can bring them to the clinic". Then we will bring them to the nearest clinic. We will get a permission letter from the office." (A5_FGD 5_SS, teacher)*

The teachers were unclear about their role in schoolchildren's health. The circular letters stated that staff members were not allowed to give medication to students, but

For peer review only

this was interpreted differently according to their personal views and the school management plan. Some teachers interpreted the circular letters to only apply to non-prescribed medication and would act *in loco parentis* and give treatment to children only if they had clear instructions from their parents. A few teachers felt able to extend this to supervising prescribed medication such as an inhaler (albeit not being sure of the technique). Other teachers would not want to handle medication at all as they viewed medication use as the responsibility of parents and children. They feared their action might worsen the student's condition, e.g., giving the wrong dose, causing side effects of medication, being blamed by parents as they were not trained to manage medical conditions.

"Sometimes parents called the teachers and said, "my child has an inhaler in his bag. Please can you supervise him"... Usually children will know how to use it. We just supervise when he is using it." (SA6_FGD 6_SS, teacher)

No communicated individual asthma action plans

The absence of a specific asthma school plan and lack of clarity about the health role of teachers could delay treatment during asthma attacks. Teachers were unsure of what to do when dealing with children with asthma symptoms, some *'panicked'* while several teachers sought help from colleagues considered to have better skills in managing asthma. Both the teachers and the school health team observed that many children with asthma did not bring their reliever inhalers to school. Without a reliever inhaler, teachers tried different strategies when their students had symptoms, e.g., relaxation and breathing techniques while waiting for the parents to arrive.

"I was shocked when she was breathless; I am not sure how to help her. I patted her back and massaged her a bit, she was better, but after that, she coughed, and I panicked and quickly called her mother." (D1_FGD 1, teacher)

For peer review only - http://bmjopen.bmj.com/site/about/guidelines.xhtml

Participants identified some poor asthma self-management practices on the part of parents. Teachers observed that parents rarely approached the school regarding their child's illness and few had shared their expectations and plans with teachers to support self-management of their child's illness at school (Table 3). Even when children had brought an inhaler to school, the teachers had not been asked to supervise its use.

> *"The child I told you earlier, his parents did not inform us... Only two parents in this school informed us about their child's illness." (HA5_IDI5, teacher)*

Suggested solutions

Participants, particularly those with asthma experience and training, identified a need for a school asthma programme to deliver asthma education and introduce an asthma school plan (Table 4). On the other hand, participants without asthma experience or training initially thought a school asthma programme was unnecessary, though some changed their views during the focus group discussions as they became aware of the risk of asthma attacks occurring at school.

> *"Asthma is not common in school. So, I don't think it (asthma programme) is necessary. But during this discussion, I think teachers should know how to help children with asthma if a child has an attack. It's better." (AN6_FGD 6, a teacher without asthma experience)*

To improve the self-management of children with asthma, healthcare professionals and some teachers suggested the school programme could target the children, their parents and teachers as primary school children were not capable of self-managing asthma independently. The children could be taught to improve their self-management skills e.g., how to avoid asthma triggers, monitor their symptoms, use a reliever inhaler, and get help if having asthma symptoms/attacks. To support the self-

For peer review only - http://bmjopen.bmj.com/site/about/guidelines.xhtml

management of the children, their parents and teachers could receive similar asthma education.

"The child themselves need to know, the severity of the symptoms,... the parents should know that the child has asthma... in the school, in case of any emergency, the teachers must know the child's asthma condition and how severe the asthma is" (SA4_IDI4, health policymaker)

Participants believed good communication with parents would help school staff to support a child's self-management. Prior agreement on emergency asthma school plans could help boost teachers' confidence in dealing with an attack and protect them from being blamed. A supporting letter from a healthcare professional regarding a child's asthma condition and individual asthma action plan was suggested as another way to provide the information to schools. A few healthcare professionals viewed schools as a platform to bridge communication with parents who did not bring their children for routine reviews.

"We need cooperation from the parent... parents should inform a teacher that 'my child has asthma and need this treatment'. So, the teacher will tell the other teachers and take care of the child... If parents provided a black and white instruction, that is even better." (T6_IDI 6, teacher)

For peer review only

284 **Table 4: Additional quotes illustrating themes for solutions, challenges and enablers.**

**Suggested solutions**
"For improvement, we should ask parents to provide the asthma book together with their immunisation records. So, we can monitor their asthma appointment." (FA3_FGD 3, school health team)
**Implementation challenges**
"We have many programmes, like the health-promoting programme, vaccination programme, the teacher also requested us to finish as soon as possible because they had to go through their education programme." (K2_IDI 2, school health team)
**Implementation enablers**
"It has to be concise and simple. I think half an hour to an hour is enough. More than that, they (children) won't be able to concentrate." (AN6, F.G.D. 6, teacher)
"SA6/RA 6: Malay can understand.<br>AN6: Even if they cannot understand, a teacher will help to explain in Tamil. No problem." (F.G.D. 6, teachers)
"We have a parent and community organisation, and the school parent and teacher organisation who can help to promote the programme. Parents can know about the programme from them, and they can come." (T6_IDI 6, teacher)

285

286 ***Implementation challenges***

287 Teachers and members of the school health team were quite negative about school
288 asthma programmes because of the workload (Table 4). Teachers were already
289 burdened with academic responsibilities and thought an intensive programme might
290 affect children's learning and general education. The school health team had similar
291 concerns and already felt overwhelmed by their workload, having to provide
292 vaccination, screenings, and general health education in all the schools in the district.

"Cannot, we have a lot of programmes. There is no time to allocate in our schedule. A programme like this, can only be done infrequently." (SA6_FGD6, teacher)

For peer review only - http://bmjopen.bmj.com/site/about/guidelines.xhtml

Teachers needed to recognise asthma symptoms/attacks and provide first aid asthma treatment, but the parents of children with asthma needed to take responsibility for self-management of their children. Parental participation in the programme was thus considered crucial but parents' attendance at school health activities was often very poor either because of work commitments or because they perceived that the health programme was not important for them.

> *"The lower education group… maybe they are not financially capable, care less about health, maybe work is a priority. So, if we invite parents to come, this group may not have time to attend." (A2_FGD 2, teacher)*

For peer review only

The target groups for the school asthma programme have different needs and capabilities. Primary schools covered a wide age range (7-12years) and the teachers' experience was that lessons needed to be customised to the child's educational level. Some teachers suggested the programme should be delivered in the native language of the children (including Tamil or Chinese as opposed to just the national language) for better understanding.

> *"HA7: English, Tamil can.*
> *VA7: But if it is in Tamil, they will understand better, because it is their native language.*
> *TA7: but some lower and upper primary can understand Malay." (FGD 7, teachers)*

Implementation enablers

Participants suggested simple, engaging, and tailored approaches catering for busy schedules, and different individual needs and capabilities (Table 4). The delivery ought to be fun and appealing to children and adults. They suggested quizzes, demonstrations, and the use of digital media such as videos to assist sessions' delivery.

> *"We can use visual aids, we teach first. After that, we ask them to demonstrate. Something like a simulation, if this happened, what will you do?" (NO7_IDI 7, teacher)*

Healthcare professionals considered good partnerships with schools as an essential enabler to promoting the uptake of school asthma programmes. The school health team had a long-term relationship with the schools and suggested various approaches to building the partnership including involvement of the school administrators at the early stage of the programme development. A good partnership with schools also helped identify and address implementation issues, for example, teachers from Chinese/Tamil schools volunteered to translate the intervention to help children who were not able to understand Malay.

> *"We have to hold a meeting with the headmaster and the teacher-in-charge of health... once the administrators are interested, they will suggest to you the teacher who will be in charge of the programme... So, you will work with the teacher to develop and run the programme." (RO_FGD 3, school health team)*

All participants proposed that healthcare professionals should deliver the intervention in schools, ideally as a multidisciplinary team including pharmacists and paediatricians. To increase parents' participation, teachers suggested wider community involvement such as parent-teacher associations, community organisations, and non-governmental organisations to help promote the intervention. Policymakers highlighted the need for introducing a national asthma school plan to improve asthma care of children during attacks.

> *"I think this matter will need a clear S.O.P. (standard operating procedure)... Although we have experience with our children, (but) for us to apply the same thing to someone's children, we are worried." (NO8_IDI8, education policymaker)*

Discussion

Summary of findings

Asthma awareness and experience of managing asthma was limited among the school staff and, with no specific asthma school plan to provide guidance, treatment

For peer review only

during asthma symptoms/emergencies could be inappropriate or delayed. School health circular letters were not clear about the teachers' health role in schools. To improve this, participants suggested a school asthma programme that focused on introducing an asthma school plan and improving awareness about asthma amongst children, their parents and teachers. Communication between healthcare professionals, parents and the school also needed improvement. The participants anticipated heavy workload, poor parental participation and diverse individual capabilities and needs as challenges to implementing school asthma programmes. Tailored approaches, good partnership with schools and multi-organisation involvement were discussed as enablers to develop and deliver the programme. We will use these findings to inform the development of a school-based asthma programme in Malaysia.

Study findings in comparison with other studies

Our interviewees suggested developing a school asthma programme involving parents and teachers, that focussed on asthma awareness, and an asthma school plan for use in attacks. Similar community-wide programmes have been implemented in other contexts.[31 32] Education for staff and children is a common component,[14 15] and could correct common misperceptions about asthma and asthma medication. Involvement of school health teams could improve the accessibility of healthcare for children with asthma.[19 33-35] School asthma programmes offer the opportunity to focus self-management education on children and involve children's social network to support their self-management skills and practices.[36 37]

For peer review only - http://bmjopen.bmj.com/site/about/guidelines.xhtml

The lack of awareness among school staff about which students had asthma, combined with poor understanding of symptoms and first aid treatment leads to hesitancy and delay in taking action risking deterioration or even death.[38 39] Delineating the health roles of teachers in an asthma school plan and providing adequate training for school staff could improve their confidence and prevent treatment delay.[39 40] However, such strategies rely on parents providing asthma reliever medication; without this, school staff will have to call parents or get immediate medical help.[38 41] School asthma guidelines in Australia, Canada, United Kingdom and United States encourage inhalers to be labelled and kept with the child or the class teacher in primary schools without a school nurse.[8 42-44] These guidelines also recommend that schools should have an emergency asthma kit to include a reliever inhaler, spacer and agreed information on when and how to use it.[8-10 43] Establishing immediate access to an emergency reliever inhaler in schools required high-level policy changes and detailed guidance for using first aid asthma treatment.[43 45] Therefore, a clear guideline to inform stakeholders e.g. policymakers, school leaders, parents and the community will aid the development and implementation of school asthma programmes.

Highlighting the importance of the programmes to stakeholders particularly policymakers could ensure successful implementation of school asthma programmes. Policy supporting improvement in school asthma management is a powerful tool in prioritising and providing resources to assist implementation. A good partnership with schools throughout the development of asthma school plans and programmes is crucial to identify challenges and enablers for successful implementation.[46 47] The presence of school plans does not guarantee teachers are equipped with the skills to manage asthma symptoms/attacks.[48] Teachers need to be aware of the school plan,

For peer review only

For peer review only - http://bmjopen.bmj.com/site/about/guidelines.xhtml

and receive education and training in the use of reliever treatment to equip them to handle asthma events in school.[48 49] Tailoring designs according to the needs and capabilities of individuals and schools could improve the uptake and mitigate the challenges of the programmes.[19 50] Healthcare providers play a crucial role in the partnership. They need to provide personalised self-management education to a child including assessing if the child can self-carry and self-administer their medication. They can also provide a personalised asthma action plan to be shared with the school.[5 45] Involving the wider community, e.g. parent and teacher association, health care providers and social services may support delivery and sustainability of school asthma programmes.[19 51 52]

Study strengths and limitations

This is one of a few studies on school asthma programmes conducted in low-middle income countries. Flexible time, venue and data collection methods facilitated recruitment of a study population with various backgrounds which contributed to the diverse views of participants. Although only three policymakers were involved in this study, we reached data saturation after the second policymaker and stopped data collection after the third when no new themes emerged. Participants with less experience on the topic may have contributed less to the focus groups though they were provided with opportunities to provide their views. Nevertheless, the findings may not be wholly applicable in other settings, and an intervention based on our findings will need adaptation for other contexts. Memos, field notes and multidisciplinary team discussions aided analysis and informed interpretation.

For peer review only

We addressed the trustworthiness of our data and analysis. Some loss of meaning may have occurred during the translation of Malay quotes; however, we analysed the data in the source language and checked the semantic and context equivalence for translated quotes. We did not conduct member checks but shared the preliminary analysis with some participants. Thematic analysis may reduce the interpretive details of each participant as the coding process focuses on the content, and less on the processes and phenomena within individuals. However, we used a pragmatic, inductive approach with constant comparisons of data and discussions to inform our understanding of the views expressed. We were aware of the risk of researcher bias in qualitative research, and actively discussed reflexivity in the regular multidisciplinary team discussions which aided analysis and informed interpretation.

For peer review only

Conclusions

School asthma programmes could improve the care and support for children with asthma. Aligned with the WHO Global School Health Initiative recommendations[1], the participants in our study highlighted the importance of tailored approaches, a good partnership with schools and engaging the wider community at an early stage of developing a school asthma programme. There is a need for a clear asthma guideline on school-based programmes to inform policymakers, school leaders, and the community in Malaysia.

Data Availability

Due to ethical concerns, supporting data cannot be made openly available and are stored under restricted access in the University of Edinburgh DataVault. Further information about the data and conditions for access are available via the University of Edinburgh Research Explorer [https://doi.org/10.7488/191f40bd-ace9-4c8a-a36c-43b796303166]. Additional non-disclosive details relating to other aspects of the data are openly available from the University of Edinburgh DataShare repository at [https://doi.org/10.7488/ds/3004].

Acknowledgements

We would like to acknowledge all the participants who voluntarily participated in this research and the local authorities for their support during the study. The RESPIRE collaboration comprises the UK Grant

holders, Partners and research teams as listed on the RESPIRE website (www.ed.ac.uk/usher/respire), including Nik Sherina Hanafi and Norita Binti Hussein.

Funding

This research was commissioned by the UK National Institute for Health Research (NIHR) Global Health Research Unit on Respiratory Health (RESPIRE), using UK Aid from the UK Government. The views expressed in this publication are those of the author(s) and not necessarily those of the NIHR or the UK Department of Health and Social Care.

Ethics Approval

Ethical approval was granted by the Malaysian National Medical Research and Ethics Committee (NMRR-18-3136-44042 (IIR)). The study conforms to the principles of the Declaration of Helsinki and ICH Guidelines for Good Clinical Practice.

Competing Interest

None declared.

Author contributions

All authors (SNR, EMK, SML, SC and HP) contributed to the study conception. SNR performed the data collection, checking and coding the transcripts. All authors (SNR, EMK, SML, SC and HP) contributed to data analysis and interpretation of data. SNR drafted the manuscript and all authors (SNR, EMK, SML, SC and HP) provided critical revisions and editing of the manuscript. SML deceased in December 2021 before submission of the second revision of the manuscript.

References

1. World Health Organisation. Making every school a health-promoting school. Geneva, Switzerland, 2021.
2. World Health Organization. Global School Health Initiatives: achieving health and education outcomes. Report of a meeting, Bangkok,Thailand, 23–25 November 2015. . Geneva, Switzerland: World Health Organisation, 2017.
3. Levinson J, Kohl K, Baltag V, et al. Investigating the effectiveness of school health services delivered by a health provider: A systematic review of systematic reviews. *PLoS One* 2019;14(6):e0212603. doi: 10.1371/journal.pone.0212603 [published Online First: 2019/06/13]
4. Global Asthma Network. The Global Asthma Report. Auckland, New Zealand, 2018.
5. Global Initiative for Asthma. Global Strategy for Asthma Prevention, 2021.
6. Pinnock H, Parke HL, Panagioti M, et al. Systematic meta-review of supported self-management for asthma: a healthcare perspective. *BMC Med* 2017;15(1):64. doi: 10.1186/s12916-017-0823-7 [published Online First: 2017/03/18]
7. Ramdzan SN, Khoo EM, Liew SM, et al. How young children learn independent asthma self-management: a qualitative study in Malaysia. *Arch Dis Child* 2020;105(9):819-24. doi: 10.1136/archdischild-2019-318127 [published Online First: 2020/07/06]
8. Asthma Australia. Asthma Guidelines for Queensland schools Australia: Asthma Australia, 2019.
9. Healthy London Partnership. London schools' guide for the care of children and young people with asthma: Pre-school, primary and secondary school years: London, 2016.
10. U.S. Department of Health and Human Services. Managing Asthma: A guide for schools: National Institutes of Health, 2014.

For peer review only - http://bmjopen.bmj.com/site/about/guidelines.xhtml

11. Scottish Intercollegiate Guidelines Network/British Thoracic Society. SIGN 158 British guideline on the management of asthma. London: Scottish Intercollegiate Guidelines Network/British Thoracic Society, 2019.
12. Piot P, Caldwell A, Lamptey P, et al. Addressing the growing burden of non-communicable disease by leveraging lessons from infectious disease management. *J Glob Health* 2016;6(1):010304-04. doi: 10.7189/jogh.06.010304
13. World Health Organisation. WHO guideline on school health services. . Geneva, 2021.
14. Harris K, Kneale D, Lasserson TJ, et al. School-based self-management interventions for asthma in children and adolescents: a mixed methods systematic review. *Cochrane Database Syst Rev,* 2019;1(1):CD011651. doi: 10.1002/14651858.CD011651.pub2 [published Online First: 2019/01/29]
15. Ramdzan SN, Suhaimi J, Harris KM, et al. School-based self-management interventions for asthma among primary school children: a systematic review. *NPJ Prim Care Respir Med* 2021;31(1):18. doi: 10.1038/s41533-021-00230-2 [published Online First: 2021/04/03]
16. Walter H, Sadeque-Iqbal F, Ulysse R, et al. Effectiveness of school-based family asthma educational programs in quality of life and asthma exacerbations in asthmatic children aged five to 18: a systematic review. *JBI Database System Rev Implement Rep* 2016;14(11):113-38. doi: 10.11124/JBISRIR-2016-003181 [published Online First: 2016/12/13]
17. Craig P, Dieppe P, Macintyre S, et al. Developing and evaluating complex interventions: the new Medical Research Council guidance. *Int J Nurs Stud* 2013;50(5):587-92. doi: 10.1016/j.ijnurstu.2012.09.010 [published Online First: 2012/11/20]
18. Wight D, Wimbush E, Jepson R, et al. Six steps in quality intervention development (6SQuID). *J Epidemiol Community Health* 2016;70(5):520-5. doi: 10.1136/jech-2015-205952 [published Online First: 2015/11/18]
19. Cicutto L, Gleason M, Haas-Howard C, et al. Building Bridges for Asthma Care Program: A School-Centered Program Connecting Schools, Families, and Community Health-Care Providers. *J Sch Nurs,* 2020;36(3):168-80. doi: 10.1177/1059840518805824 [published Online First: 2018/10/20]
20. Ahad A, Khoo EM. Asthma Control and Care Among Malaysian Primary School Children: A Cross-Sectional Study. *Asia Pac J Public Health* 2017;29(5):422-29. doi: 10.1177/1010539517717766 [published Online First: 2017/07/19]
21. Asher MI, Montefort S, Bjorksten B, et al. Worldwide time trends in the prevalence of symptoms of asthma, allergic rhinoconjunctivitis, and eczema in childhood: ISAAC Phases One and Three repeat multicountry cross-sectional surveys. *Lancet (London, England)* 2006;368(9537):733-43. doi: 10.1016/S0140-6736(06)69283-0
22. Wong GW, Kwon N, Hong JG, et al. Pediatric asthma control in Asia: phase 2 of the Asthma Insights and Reality in Asia-Pacific (AIRIAP 2) survey. *Allergy* 2013;68(4):524-30. doi: 10.1111/all.12117 [published Online First: 2013/02/19]
23. Kementerian Kesihatan Malaysia. Garis Panduan Perkhidmatan Kesihatan Sekolah. Malaysia, 2013.
24. Ramdzan SN, Pinnock H, Liew SM, et al. Perceptions of complementary/alternative medicine use and influence on evidence-based asthma medicine adherence in Malaysian children. *NPJ Prim Care Respir Med*

For peer review only

For peer review only - http://bmjopen.bmj.com/site/about/guidelines.xhtml

2019;29(1):5. doi: 10.1038/s41533-019-0118-x [published Online First: 2019/02/26]

25. Malaysia Education Statistics. Quick Facts 2018. Malaysia: Educational Planning and Research Division, Ministry of Education Malaysia, 2018.

26. Braun V, Clarke V. Succesful Qualitative Research: a practical guide for beginners. London: SAGE Publication Ltd 2013.

27. Silverman D. Doing Qualitative Research. 5th edition ed. London, United Kingdom: SAGE Publication Limited 2018.

28. Farre A, Wood V, McDonagh JE, et al. Health professionals' and managers' definitions of developmentally appropriate healthcare for young people: conceptual dimensions and embedded controversies. *Arch Dis Child* 2016;101(7):628-33. doi: 10.1136/archdischild-2015-309473 [published Online First: 2016/03/06]

29. Borrell-Carrio F, Suchman AL, Epstein RM. The biopsychosocial model 25 years later: principles, practice, and scientific inquiry. *Ann Fam Med* 2004;2(6):576-82. doi: 10.1370/afm.245 [published Online First: 2004/12/04]

30. Grbich C. Qualitative Data Analysis: An introduction. Second ed. United Kingdom: SAGE Publication 2013.

31. Lenney W, Bush A, Fitzgerald DA, et al. Improving the global diagnosis and management of asthma in children. *Thorax* 2018;73(7):662-69. doi: 10.1136/thoraxjnl-2018-211626

32. Szefler SJ, Fitzgerald DA, Adachi Y, et al. A worldwide charter for all children with asthma. *Pediatr Pulmonol* 2020;55(5):1282-92. doi: 10.1002/ppul.24713 [published Online First: 2020/03/07]

33. Suwannakeeree P, Deerojanawong J, Prapphal N. School-Based Educational Interventions Can Significantly Improve Health Outcomes in Children with Asthma. *J Med Assoc Thai* 2016;99(2):166-74. [published Online First: 2016/06/03]

34. Clark NM, Gong M, Kaciroti N, et al. A trial of asthma self-management in Beijing schools. *Chronic illness* 2005;1(1):31-8. doi: 10.1177/17423953050010010101 [published Online First: 2006/12/02]

35. Carvalho Coelho AC, Barretto Cardoso LS, de Souza-Machado C, et al. The Impacts of Educational Asthma Interventions in Schools: A Systematic Review of the Literature. *Can Respir J,* 2016;2016:8476206. doi: 10.1155/2016/8476206 [published Online First: 2016/09/23]

36. Szefler SJ, Cloutier MM, Villarreal M, et al. Building Bridges for Asthma Care: Reducing school absence for inner-city children with health disparities. *J Allergy Clin Immunol* 2019;143(2):746-54 e2. doi: 10.1016/j.jaci.2018.05.041 [published Online First: 2018/07/29]

37. Lemanske RF, Jr., Kakumanu S, Shanovich K, et al. Creation and implementation of SAMPRO: A school-based asthma management program. *J Allergy Clin Immunol* 2016;138(3):711-23. doi: 10.1016/j.jaci.2016.06.015 [published Online First: 2016/09/07]

38. Greiling AK, Boss LP, Wheeler LS. A preliminary investigation of asthma mortality in schools. *J Sch Health,* 2005;75(8):286-90. doi: 10.1111/j.1746-1561.2005.00039.x [published Online First: 2005/09/24]

39. Murphy KR, Hopp RJ, Kittelson EB, et al. Life-threatening asthma and anaphylaxis in schools: a treatment model for school-based programs. *Ann Allergy Asthma Immunol* 2006;96(3):398-405. doi: 10.1016/S1081-1206(10)60906-4 [published Online First: 2006/04/07]

For peer review only - http://bmjopen.bmj.com/site/about/guidelines.xhtml

40. Kew KM, Carr R, Donovan T, et al. Asthma education for school staff. *Cochrane Database Syst Rev,* 2017;4:CD012255. doi: 10.1002/14651858.CD012255.pub2 [published Online First: 2017/04/13]

41. Gerald JK, Stroupe N, McClure LA, et al. Availability of Asthma Quick Relief Medication in Five Alabama School Systems. *Pediatr Allergy Immunol Pulmonol* 2012;25(1):11-16. doi: 10.1089/ped.2011.0118 [published Online First: 2012/03/29]

42. Council on School H. Policy statement--guidance for the administration of medication in school. *Pediatrics* 2009;124(4):1244-51. doi: 10.1542/peds.2009-1953 [published Online First: 2009/09/30]

43. Department of Health. Guidance on the use of emergency salbutamol inhalers in schools. Storage and care of the inhaler. United Kingdom, 2015.

44. OPHEA Health schools and Healthy Communities. Creating Asthma friendly schools. Canada, 2015.

45. Toups MM, Press VG, Volerman A. National Analysis of State Health Policies on Students' Right to Self-Carry and Self-Administer Asthma Inhalers at School. *J Sch Health,* 2018;88(10):776-84. doi: 10.1111/josh.12681 [published Online First: 2018/09/12]

46. Cicutto L, Gleason M, Szefler SJ. Establishing school-centered asthma programs. *J Allergy Clin Immunol* 2014;134(6):1223-30. doi: 10.1016/j.jaci.2014.10.004 [published Online First: 2014/12/09]

47. Kakumanu S, Lemanske RF, Jr. Asthma in Schools: How School-Based Partnerships Improve Pediatric Asthma Care. *Immunol Allergy Clin North Am* 2019;39(2):271-81. doi: 10.1016/j.iac.2018.12.011 [published Online First: 2019/04/08]

48. Jaramillo Y, Reznik M. Do United States' teachers know and adhere to the national guidelines on asthma management in the classroom? A systematic review. *The Scientific World Journal* 2015;2015:624828. doi: 10.1155/2015/624828 [published Online First: 2015/03/03]

49. Cain A, Reznik M. Asthma management in New York City schools: A classroom teacher perspective. *J Asthma* 2016;53(7):744-50. doi: 10.3109/02770903.2015.1135946 [published Online First: 2016/04/01]

50. Brigden A, Parslow RM, Linney C, et al. How are behavioural interventions delivered to children (5-11 years old): a systematic mapping review. *BMJ Paediatr Open* 2019;3(1):e000543. doi: 10.1136/bmjpo-2019-000543 [published Online First: 2020/01/08]

51. McCabe EM, Kaskoun JR, Murphy EL, et al. An Evidence-Based Framework for Implementation of a School-Community Partnership. *NASN Sch Nurse* 2021;36(3):164-69. doi: 10.1177/1942602X20964765 [published Online First: 2020/10/16]

52. Clark NM, Lachance L, Doctor LJ, et al. Policy and system change and community coalitions: outcomes from allies against asthma. *American journal of public health* 2010;100(5):904-12. doi: 10.2105/AJPH.2009.180869 [published Online First: 2010/03/18]

Figure legend

Figure 1: Themes and subthemes of participants' views of asthma and school-based asthma intervention.

For peer review only - http://bmjopen.bmj.com/site/about/guidelines.xhtml

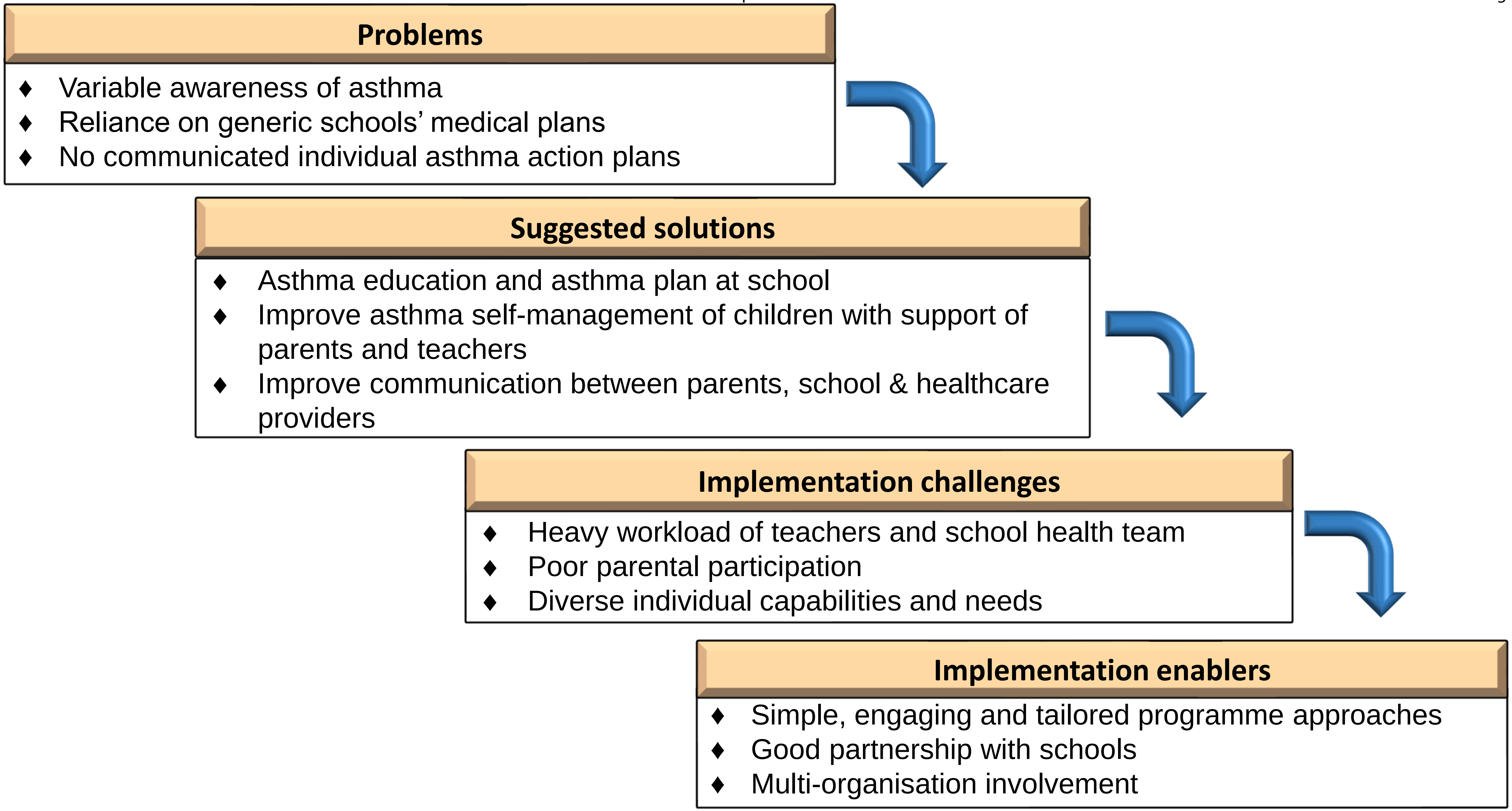

Figure 1 : Themes and subthemes of participants' views of asthma and school-based asthma intervention.

For peer review only - http://bmjopen.bmj.com/site/about/guidelines.xhtml

Borang soal selidik latar belakang
Socio-demographic questionnaire

Latar belakang
Background

Umur*(Age)* : ___________

Jantina*(Gender)* : ___________

Bangsa*(Ethnicity)* : ___________

Status perkahwinan *(Marital status)*:

Bujang*(Single)*/Bercerai *(Divorced)*/Berkahwin*(Married)*

Tahap pendidikan tertinggi *(Highest educational level achieved):*

Rendah*(Primary)*/Menengah *(Secondary)*/Pengajian Tinggi *(Tertiary)*

Pekerjaan *(Occupation)* : ____________________

Pengalaman berkerja (work experience) : ………… /tahun(year)

Anggaran pendapatan isi rumah

(Estimated family income): RM ________ /bulan(month)

Informasi mengenai asma
Information about asthma

Adakah anda menghidap asma *(Do you have asthma)?*

Ya *(yes)*/ Tidak *(No)*

Adakah ahli keluarga menghidap asma (Anyone in your family has asthma)?

Ya *(yes)*/ Tidak *(No)*

Jika Ya, sila nyatakan (If yes, (please specify):

____

For peer review only

Childhood Asthma
Participant Interview Topic Guide (school staff)

Preamble:

- **Ice-breaking and explain that there is no right or wrong answer**
- **Explain need to get consent for the interview and audio-recording. Obtain consents.**
- **Explain that the participant does not have to answer if he or she does not wish to do so**

Question	Prompts
**Asthma experience**	
Tell us your experience in dealing with children with asthma?	Your child? Your student?
What do you know about asthma?	Experience? Reading? Told by someone?
How do you know if a child's asthma is good or badly controlled?	Able to play? Easy breathing? No cough? How do you know about this?
How does a child's asthma disturb/not disturb he/her? How?<br>How does a child's asthma disturbs/not disturb you? How?	Activities affected e.g. attending school?<br>*Pendidikan Jasmani dan kesihatan* (PJK)/sports?
In your opinion, how does having asthma makes a child feel?	Emotionally-friends isolation/bully? Shy to use medication?
**Asthma treatment**	
Do you know of any asthma treatment?	Pills? MDI? Spacer? Aerochamber?
How about complementary and alternative medicine for asthma?	
**Asthma management at school**	
What do you do when a student has an asthma attack?	Inhaler? Go to the hospital/clinic? Call parents?
How do you recognise a severe asthma attack?<br>What would you do if this happened?	Breathlessness? Unable to speak?
Is there anything else you would do?	Concern on the administration of medication eg MDI to student(s)? Rules or legislation?
Is there any rules/limitation for students with asthma at school?	Sports? Physical activity? Extracurricular activities?
Is there any asthma education or campaign at school?	How is it delivered? By whom? To who?
What do you think about asthma education at school? Is it necessary?	Feasible? How best to deliver? Parents and school staffs involvement? How often?
What do you think if school staffs are allowed to deliver/supervise the use of asthma medication e.g inhaler at school?	Good? Not good? Concerns?

For peer review only

Suggestions/ideas for intervention?<br><br>Any suggestion on how to improve asthma management at school?	Others? Timing – during or after school?
**Health belief about asthma**<br><br>Why do you think a child has asthma?<br><br>What do you think a child can do to prevent asthma?	 <br>Inherited? Infection? Environment?<br><br>Avoidance of food? Medication? Sports?
**Source of information**<br><br>Where and from whom did you get information about asthma?<br><br>Anything else that you want to share about childhood asthma?	 <br>Clinic? Family? Friends? Internet? How good is the information?

For peer review only

Childhood Asthma (CuT-AsthMa)
Participant Interview Topic Guide (School Health Team)

Preamble:
- **Ice-breaking and explain that there is no right or wrong answer**
- **Explain the need to get consent for the interview and audio-recording. Obtain consents.**
- **Explain that the participant does not have to answer if he or she does not wish to do so**

Question	Prompts
**Asthma experience**	
Tell us your experience about dealing with children with asthma?	A student? A patient? Your own child?
How do you know that they have asthma?	Symptoms e.g cough, wheezing? Informed by parents?
How do you know if the child's asthma is good or badly controlled?	Able to play? Easy breathing? No cough? Where do you know about this?
How does a student's asthma disturb/not disturb him/her?	Physically? Activities affected at school e.g PJK, absent from school?
How does a student's asthma disturbs/not disturb you? How?	
In your opinion, how does having asthma makes a student feel?	Emotionally-friends isolation/bully? Shy to use medication?
**Asthma treatment**	
Do you know of any asthma treatment?	Pills? MDI? Spacer? Aerochamber?
How about complementary and alternative medication for asthma?	
**Asthma management at school**	
What do you do when a student has asthma attack?	Inhaler? Go to hospital/clinic? Asthma action plan? How do you know this?
How do you recognise a severe asthma attack? What would you do if this happened?	Breathlessness? Unable to speak?
Is there anything else you would do?	
	Sports? Physical activity? Extracurricular activities?

For peer review only

Is there any rules/limitation for students with asthma at school?<br><br>Is there any asthma education or campaign at school?<br><br>What do you think of asthma education at school? Is it necessary? Is it feasible?<br><br>What is your opinion on teachers delivering/supervising the use of asthma medication e.g inhaler at school?<br><br>Any suggestion on how to improve asthma management at school?	How is it delivered? By whom? To who?<br><br>Feasible? How best to deliver? Parents and teachers involvement? How often?<br><br>Good? Not good? Any worries?
**Health belief about asthma**<br><br>Why do you think a child has asthma?<br><br>What do you think a child can do to prevent asthma?	Inherited? Infection? Environment?<br><br>Avoidance of food? Medication? Sports?
**Source of information**<br><br>Where and from whom did you get information about asthma?<br><br>Anything else that you want to share about asthma among children?	Clinic? Family? Friends? Internet? How good is the information?

For peer review only

Childhood Asthma (CuT-AsthMa)
Participant focus group topic guide (Healthcare professionals/policymakers)
(Interviews, which will be offered as an option to suit the convenience/preference of the participant, will follow the same topic guide)

Preamble:

- **Ice-breaking and explain that there is no right or wrong answer**
- **Explain the need to get consent for the focus group/interview and audio-recording. Obtain consents.**
- **Explain that the participant does not have to answer if he or she does not wish to do so**

Question	Prompts
**Asthma experience**<br><br>Tell us about your experience in dealing with children with asthma?<br><br>How do you know that they have asthma?<br><br>How do you know if the child's asthma is good or badly controlled?<br><br>How does a student's asthma disturb/not disturb him/her? How does a student's asthma disturbs/not disturb you? How?<br><br>In your opinion, how does having asthma makes a student feel?	A student? A patient? Your child?<br><br>Symptoms e.g cough, wheezing? Informed by parents?<br><br>Able to play? Easy breathing? No cough? Where do you know about this?<br><br>Physically? Activities affected at school e.g PJK, absent from school?<br><br>Emotionally-friends isolation/bully? Shy to use medication?
**Asthma treatment**<br><br>Do you know of any asthma treatment?<br><br>How about complementary and alternative medicine for asthma?	Pills? MDI? Spacer? Aerochamber?
**Asthma management at school**<br><br>What do you do when a student has an asthma attack?<br><br>How do you recognise a severe asthma attack? What would you do if this happened?<br><br>Is there anything else you would do?<br><br>Is there any rules/limitation for students with asthma at school?<br><br>Is there any asthma education or campaign at school?	Inhaler? Go to the hospital/clinic? Asthma action plan? How do you know this?<br><br>Breathlessness? Unable to speak?<br><br>Sports? Physical activity? Extracurricular activities?<br><br>How is it delivered? By whom? To who?
What do you think of asthma education at school? Is it necessary? Is it feasible?	Feasible? How best to deliver? Parents and teachers involvement? How often?

For peer review only - http://bmjopen.bmj.com/site/about/guidelines.xhtml

What is your opinion on teachers delivering/supervising the use of asthma medication e.g inhaler at school?<br><br>Any suggestion on how to improve asthma management at school?	Good? Not good? Any worries?
**Health belief about asthma**<br><br>Why do you think a child has asthma?<br><br>What do you think a child can do to prevent asthma?	 <br>Inherited? Infection? Environment?<br><br>Avoidance of food? Medication? Sports?
**Source of information**<br><br>Where and from whom did you get information about asthma?<br><br>Anything else that you want to share about asthma among children?	 <br>Clinic? Family? Friends? Internet? How good is the information?

For peer review only - http://bmjopen.bmj.com/site/about/guidelines.xhtml

Standards for Reporting Qualitative Research (SRQR)*

http://www.equator-network.org/reporting-guidelines/srqr/

Title and abstract

	Page/line no(s).
**Title** - Concise description of the nature and topic of the study Identifying the study as qualitative or indicating the approach (e.g., ethnography, grounded theory) or data collection methods (e.g., interview, focus group) is recommended	1/1-3
**Abstract** - Summary of key elements of the study using the abstract format of the intended publication; typically includes background, purpose, methods, results, and conclusions	2-3/27-54

Introduction

**Problem formulation** - Description and significance of the problem/phenomenon studied; review of relevant theory and empirical work; problem statement	4-5/70-105
**Purpose or research question** - Purpose of the study and specific objectives or questions	5/101-105

Methods

**Qualitative approach and research paradigm** - Qualitative approach (e.g., ethnography, grounded theory, case study, phenomenology, narrative research) and guiding theory if appropriate; identifying the research paradigm (e.g., postpositivist, constructivist/ interpretivist) is also recommended; rationale**	7-6/154-158
**Researcher characteristics and reflexivity** - Researchers' characteristics that may influence the research, including personal attributes, qualifications/experience, relationship with participants, assumptions, and/or presuppositions; potential or actual interaction between researchers' characteristics and the research questions, approach, methods, results, and/or transferability	8/163-171
**Context** - Setting/site and salient contextual factors; rationale**	6/116-128
**Sampling strategy** - How and why research participants, documents, or events were selected; criteria for deciding when no further sampling was necessary (e.g., sampling saturation); rationale**	1/36-37 and 7/141-143
**Ethical issues pertaining to human subjects** - Documentation of approval by an appropriate ethics review board and participant consent, or explanation for lack thereof; other confidentiality and data security issues	6/108-114
**Data collection methods** - Types of data collected; details of data collection procedures including (as appropriate) start and stop dates of data collection and analysis, iterative process, triangulation of sources/methods, and modification of procedures in response to evolving study findings; rationale**	7/133-144

For peer review only

For peer review only - http://bmjopen.bmj.com/site/about/guidelines.xhtml

**Data collection instruments and technologies** - Description of instruments (e.g., interview guides, questionnaires) and devices (e.g., audio recorders) used for data collection; if/how the instrument(s) changed over the course of the study	7/143-144
**Units of study** - Number and relevant characteristics of participants, documents, or events included in the study; level of participation (could be reported in results)	8-9/180-188 (Table 1)
**Data processing** - Methods for processing data prior to and during analysis, including transcription, data entry, data management and security, verification of data integrity, data coding, and anonymization/de-identification of excerpts	8/-143-151
**Data analysis** - Process by which inferences, themes, etc., were identified and developed, including the researchers involved in data analysis; usually references a specific paradigm or approach; rationale**	7-8/154-171
**Techniques to enhance trustworthiness** - Techniques to enhance trustworthiness and credibility of data analysis (e.g., member checking, audit trail, triangulation); rationale**	7-8/156-16

Results/findings

**Synthesis and interpretation** - Main findings (e.g., interpretations, inferences, and themes); might include development of a theory or model, or integration with prior research or theory	8-15/180-308, Figure 1
**Links to empirical data** - Evidence (e.g., quotes, field notes, text excerpts, photographs) to substantiate analytic findings	8-15/quotes and Table 3-4

Discussion

**Integration with prior work, implications, transferability, and contribution(s) to the field -** Short summary of main findings; explanation of how findings and conclusions connect to, support, elaborate on, or challenge conclusions of earlier scholarship; discussion of scope of application/generalizability; identification of unique contribution(s) to scholarship in a discipline or field	15-19/311-392
**Limitations** - Trustworthiness and limitations of findings	18-19/373-392

Other

**Conflicts of interest** - Potential sources of influence or perceived influence on study conduct and conclusions; how these were managed	19/422
**Funding** - Sources of funding and other support; role of funders in data collection, interpretation, and reporting	19/416-419

*The authors created the SRQR by searching the literature to identify guidelines, reporting standards, and critical appraisal criteria for qualitative research; reviewing the reference lists of retrieved sources; and contacting experts to gain feedback. The SRQR aims to improve the transparency of all aspects of qualitative research by providing clear standards for reporting qualitative research.

For peer review only

For peer review only - http://bmjopen.bmj.com/site/about/guidelines.xhtml

**The rationale should briefly discuss the justification for choosing that theory, approach, method, or technique rather than other options available, the assumptions and limitations implicit in those choices, and how those choices influence study conclusions and transferability. As appropriate, the rationale for several items might be discussed together.

Reference:

O'Brien BC, Harris IB, Beckman TJ, Reed DA, Cook DA. **Standards for reporting qualitative research: a synthesis of recommendations.** *Academic Medicine*, Vol. 89, No. 9 / Sept 2014 DOI: 10.1097/ACM.0000000000000388

For peer review only
